# Automating Middle-Down Mass Spectrometry Analysis for Extensive Antibody Characterization

**DOI:** 10.1021/acs.analchem.5c06408

**Published:** 2026-04-13

**Authors:** Amy K. Carfagno, Linda B. Lieu, Jake T. Kline, Luca Fornelli, Kenneth R. Durbin

**Affiliations:** † School of Biological Sciences, 6187 University of Oklahoma, Norman, Oklahoma 73019, United States; ‡ Department of Chemistry and Biochemistry, 6187 University of Oklahoma, Norman, Oklahoma 73019, United States; § Proteinaceous, Inc., Evanston, Illinois 60204, United States

## Abstract

Workflows and software tools for peptide mapping have been extensively optimized and simplified for widespread use. However, top-down and middle-down workflows, despite offering valuable proteoform information not obtainable by peptide mapping, remain demanding in terms of user expertise as well as instrument setup, data analysis, and result validation. Current software offerings have also not been tailored to address targeted protein characterization workflows, often necessitating multiple software tools to accomplish data analysis. To move toward routine intact protein characterization workflows with a single software tool, an automated platform named Proteoform Studio is introduced here. Proteoform Studio features a complete analysis pipeline, allowing a user to set up instrument runs, modify acquisition methods, rapidly deconvolute data to define intact mass features, search results to identify proteoforms, and obtain the highest quality proteoform characterization through automated aggregation of fragmentation results. Additionally, fragment ion matching settings for signal-to-noise ratio and isotope fit score were identified that produced automated matching results well-aligned with manual validation results, thereby reducing the need for time-consuming manual verification. With Proteoform Studio analysis, >80% sequence coverage of light chain (Lc) and the N-terminal portion of heavy chain (Fd) antibody subunits was achieved in an automated fashion using a middle-down approach. These sequence coverage results utilized aggregation of spectral results from different fragmentation methods to take advantage of complementary fragment ion formation. Overall, introducing software to automate antibody subunit analysis will lower barriers to entry and eliminate data analysis bottlenecks, driving adoption in biopharmaceutical assays and ultimately leading to more precise characterization of therapeutic proteoform landscapes.

## Graphical abstract

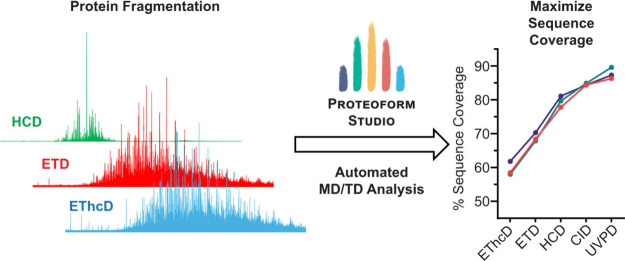

## Introduction

By specifying all the different protein forms found in systems as discrete proteoforms, a precise accounting can be obtained of all structural differences in gene products, from variation at the genetic level to co- and post-translational modifications.^1–3^ Recent studies support the idea that accounting for such differences, particularly within the same protein family, allows for more accurate modeling of differences in disease phenotypes.^4–6^ However, bottom-up approaches are not well suited to proteoform identification due to the challenge of unambiguously mapping proteolytic peptides to intact proteoforms.^7^ Top-down approaches are valuable in this regard,^2^ though a number of analytical and technical hurdles have resulted in more limited application of these approaches as compared to bottom-up workflows. For example, the trypsin digestion commonly employed in bottom-up workflows yields a relatively structurally uniform set of peptides in terms of size (generally 0.6–2 kDa) and gas-phase charge (generally +2–3).^8^ Sequencing of tryptic peptides via mass spectrometry (MS) may be accomplished fairly consistently by applying standard collisional dissociation methods. However, for top-down workflows, structural variability of proteins is more pronounced than that of peptides from protein digests, requiring more work-intensive optimization of fragmentation parameters.^9–11^ In addition, given the challenge of comprehensively fragmenting large biomolecules in the gas-phase, combining complementary fragmentation techniques may be necessary for thorough proteoform characterization.^12,13^ Though several software tools for top-down data analysis have been developed,^11,14–18^ in general, options are more limited and often more demanding in terms of user expertise as compared to those available for bottom-up. Further, top-down mass spectrometry software tools are often oriented toward proteomics applications and have not been tailored to provide deep sequencing for more routine or targeted proteoform characterization studies. For instance, complementary fragment information is often not incorporated or the tool is too reliant on user input for automated analysis. While different software tools can perform one or more pieces that are necessary for the full workflow, no single software has put all of the pieces together.

Conceptually, top-down data analysis workflows involve the deconvolution of mass spectral data to determine the mass species present in a sample, followed by defining the time range that mass species are detectable and then finally performing proteoform quantitation and identification. Deconvolution transforms mass spectral data in the *m*/*z* domain to yield a zero-charge spectrum.^19^ Results from deconvolution of experimental data in the *m*/*z* domain allow for the determination of proteoform mass features. By incorporating sliding window deconvolution to define an elution profile for each proteoform, features can be resolved in both mass and time; these features are referred to hereafter as mass components. In general, detecting mass components involves merging signals with similar mass and retention time within a defined tolerance into a single mass component. Deconvolution and mass component detection settings may be tuned to balance sensitivity of component detection with accuracy.^20,21^ Intact masses, determined from deconvolution and mass component identification, may be paired with fragmentation data for proteoform identification.

For low resolution data, the charge state may be determined based on matching observed *m*/*z* signal patterns to theoretical charge state distributions. Existing algorithms for charge state deconvolution can mostly be categorized as variations on the theme of the maximum entropy algorithm, MaxEnt, and can be found in different instrument vendor software packages. There also exist several algorithms with substantial advances to the MaxEnt algorithm, such as PMI Intact and UniDec.^22–25^ In this “forward” approach, rather than working backward from the experimental spectrum to generate a set of neutral masses, a theoretical spectrum is generated and compared to the experimental data, accounting for expected effects of noise on peak broadening. Another type of charge state deconvolution algorithm, kDecon, takes an iterative approach to charge state assignments, resulting in a much less computationally intensive, yet still accurate, deconvolution.^26^ At sufficiently high resolution, the spacing between consecutive isotopologue peaks can be used to directly calculate the charge. Options for processing high-resolution data include Xtract,^27^ MS-Deconv,^28^ and THRASH.^29^ Another approach to charge state assignment using universal spacing patterns in log­(*m*/*z*) space was implemented in FLASHDeconv and can process both isotopically resolved and unresolved data.^30^

Multiple types of software for proteoform identification from top-down proteomics data exist. MS-Align+ and its successor TopPIC implement a spectral alignment approach that considers mass shifts between observed monoisotopic masses and theoretical fragments of a candidate proteoform to account for both commonly observed off-by-one deconvolution errors as well as presence of unknown post-translational modifications (PTMs) before calculating the statistical significance of a proteoform-spectrum match.^28,31,32^ Efficient PTM identification is also supported by MetaMorpheus, which can process both top-down and bottom-up data sets via a multinotch search approach (analogous to the isotope error option in other software) that considers a set of predefined PTMs.^33^ ProSightPD, which is implemented as nodes in Proteome Discoverer, also supports proteoform identification via a database search to look for a variety of different proteoforms including full-length, truncated, modified with a known annotated PTM, or modified with an unknown mass shift that corresponds to the difference in mass between theoretical and observed precursors.^34^

For experiments in which only a handful of candidates are being considered, more targeted approaches can be taken. For instance, TDValidator implements a different approach to proteoform identification. Rather than determining neutral fragment masses from matching observed experimental data to an averagine model,^27^ TDValidator starts from a defined candidate proteoform sequence and calculates theoretical fragment ions. Isotope distributions are calculated based on the fragment ion’s chemical formula and matched to observed signal in the experimental data with consistency of agreement between theoretical and observed signal quantified by a fitter score on the scale of 0 to 1.^12^

Overall, a number of tools supporting different aspects of the top-down data analysis workflow have been developed, yet the need remains for an integrated platform supporting all aspects of a targeted top-down-middle-down protein characterization workflow. For example, though ProSightPD provides deconvolution and search, it is not designed to make use of multiple spectra to increase the sequence coverage of a proteoform. Conversely, BioPharma Finder does incorporate multiple spectral results together but does not provide precursor mass deconvolution in its top-down workflow. Additionally, in BioPharma Finder, all spectral fragment matches are pooled together at the end without any additional burden of evidence (e.g., multiple observations are not required for each fragment ion). Other tools, such as MASH Native and ProSight Native represent solutions incorporating multiple tools for mass deconvolution and proteoform identification. However, the primary focus of both platforms for top-down data is targeted analysis that is user driven and therefore not suited for high-throughput or automated analyses.^18,35^ In the context of monoclonal antibody subunit characterization, previous middle-down approaches have required multiple separate informatics tools and extensive manual validation to be combined. Below, we highlight several multitool workflows that were put together to accomplish data analysis. Oates et al. applied proton transfer charge reduction (PTCR) to characterize subunits generated from IdeS digestion of three monoclonal antibody standards. BioPharma Finder was used for sliding window deconvolution of MS1 data, and MS2 and PTCR MS3 spectra were batch-processed with TDValidator in ProSight Native to optimize fragmentation parameters. Fragment ion assignments from spectra acquired using optimized parameters were manually validated in TDValidator. In an additional step, composite coverage was determined by overlaying fragment maps from separate fragmentation techniques and manually counting observed cleavage sites.^36^ Dhenin et al. characterized the light and heavy chains generated from reduction of two commercial monoclonal antibodies via several fragmentation techniques employing a range of collision energies for collisional dissociation techniques and reaction time and supplemental activation energy settings for electron transfer dissociation. Time-resolved deconvolution and MaxEnt were used for intact mass profiling from MS1 data in Genedata Expressionist, and Xtract in FreeStyle (Thermo Scientific) was used for MS2 deconvolution. Monoisotopic mass lists were used as input to ProSight Lite for fragment ion assignment with the results further evaluated using TDFragMapper. A custom script was developed for aggregation of results from multiple spectra and fragmentation techniques.^10^ For subunit analysis using data from a SCIEX ZenoTOF 7600 instrument, the following multistep data analysis process was implemented: .wiff files were converted with MSConvert before manually deconvoluting regions of interest in MASH Explorer, exporting the fragment ion masses to Excel, and then processing in ProSightPC.^37^

Here, we present Proteoform Studio, an end-to-end software platform that allows users to process middle- and top-down data in a seamless and automated manner without the feature gaps of previous software platforms. Proteoform Studio is tailored to maximize the usefulness of different complementary fragmentation techniques present on modern mass spectrometers. As such, one of the novel features introduced by Proteoform Studio is the ability to aggregate results from multiple spectra such that the complementary ions can be combined to more thoroughly characterize an individual proteoform. These additional fragmentation data can be especially valuable for improving sequence coverage in middle- and top-down experiments, as larger molecules often need complementary fragmentation information to sufficiently resolve proteoform species. Furthermore, Proteoform Studio allows for sliding window deconvolution of fragmentation data to increase sensitivity in much the same way that sliding window deconvolution has for intact mass analysis.^20^ Here, the integrated data processing workflow of Proteoform Studio is applied to the middle-down characterization of two ∼25 kDa subunits, namely, the light chain (Lc) and N-terminal portion of the heavy chain (Fd), of monoclonal antibody standards, analyzed both individually and in a mixture (Figure 1).^38^ Proteoform Studio was used to inform target selection from the intact mass results and improve subunit characterization via aggregation of multiple fragmentation modes, especially for relatively lower injection amounts in the presence of a background matrix. Overall, the middle-down antibody subunit characterization workflow presented here demonstrates how Proteoform Studio can process and annotate fragmentation ion data in an automated manner that closely tracks manually validated results while simultaneously utilizing all available fragmentation data to improve proteoform sequence coverage.

**Figure 1:**
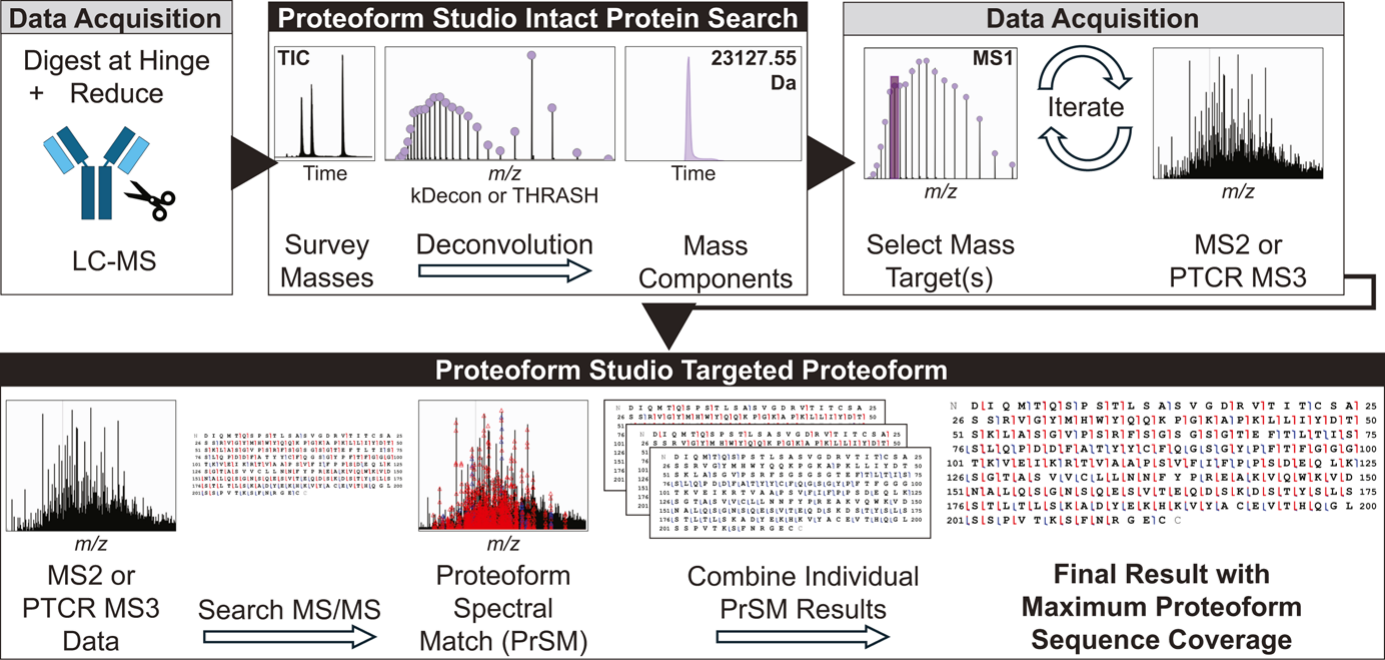
Proteoform Studio workflow for middle-down antibody subunit analysis. Subunits of the NIST mAb standard were analyzed alone and in mixtures of varying complexity by applying the integrated data processing workflow of Proteoform Studio to define targets and improve sequence coverage by automated aggregation of fragment ion assignments from multiple fragmentation techniques. Monoclonal antibodies were digested using a middle-down enzyme into large subunits (i.e., Fd, Fc, and Lc) and then analyzed by LC-MS. Survey subunit masses were deconvoluted into time-resolved mass components. Mass targets were then selected from the deconvoluted charge state distribution for subsequent fragmentation analysis using MS2 or PTCR MS3. Multiple rounds of fragmentation were performed with different fragmentation techniques. All fragmentation data were input into Proteoform Studio, which processed each spectrum using search and isotopic fitting routines to generate individual Proteoform Spectral Matches (PrSMs). These PrSM results were automatically compiled into an aggregate history to maximize the sequence coverage of each targeted mass component.

## Materials and Methods

### Software Methodology

Proteoform Studio is a Windows Presentation Format (WPF) application developed in .NET 8 with C# that can process Thermo .raw files as well as .mzml files. The two main workflows in the software are the intact protein workflow and the targeted protein workflow. The intact protein workflow incorporates deconvolution of precursor spectra to determine the mass components. Proteoform Studio uses a sliding window deconvolution approach to deconvolute intact mass spectra. The kDecon and THRASH algorithms allow for the deconvolution of isotopically unresolved and resolved spectra, respectively. Fragment scans from all files are associated with mass components that have a charge state falling within the tolerance of the isolation target from the fragmentation spectra. Fragment scans are searched against a database of candidate proteins and putative modifications. Search hits with at least the minimum required number of fragment ion matches will have all possible theoretical ions generated from the chemical formula of the matched proteoform for subsequent isotopic fitter analysis of future mass-component-associated fragment scans. The noise level in the spectrum is defined by the sampled noise array in the .raw file. The targeted proteoform workflow directly matches theoretical fragment ions of proteoforms specified by the user via .fastp file. A .fastp file is similar to .fasta format but with all proteoform features expressed in ProForma notation.^39^ The peak mode that is part of the targeted proteoform mode allows a user to set ranges of spectra that are associated with one or more proteoforms.

### Antibody Sample Preparation and LC-MS/MS Analysis

The NIST SRM 8671 monoclonal antibody standard (National Institutes of Standards and Technology) was subjected to limited proteolysis and reduction and analyzed via LC-MS/MS both alone and spiked into a simple antibody standard mixture as well as into a plasma IgG background; details are provided in the Supplementary Methods.^36,40,41^

### Data Analysis

All mass spectrometry data were analyzed with Proteoform Studio v1.0.25206.1 (Proteinaceous, Inc., Evanston, IL). Intact mass data were obtained using the intact protein analysis workflow, accounting for N-terminal pyroglutamic acid modification of the NIST Fd subunit. Target selection was based on the set of most intense charge states identified by Proteoform Studio for the Lc and Fd subunits with lower, intermediate, and higher charge states targeted for collisional dissociation, UVPD, and electron-based fragmentation methods, respectively (Figure S1). Mass target scheduling was refined based on review of raw chromatogram data (Figure S2). Sequence coverage for each fragmentation method was evaluated by using the targeted proteoform data analysis workflow in Proteoform Studio. To inform the optimization of fragmentation settings, composite coverage for combinations of fragmentation techniques was evaluated by using Proteoform Studio to aggregate fragment match results from multiple data files. Fragmentation settings were further adjusted based on checking residual precursor signal and fragment ion size in the raw data. Final targeted data analysis results presented herein used fragment signal-to-noise ratio and minimum isotope fit score (10 and 0.68, respectively) defined based on settings providing consistency with sequence coverage results from manual validation of fragment ion assignment. Specifically, a subset of files from the preliminary data set was manually validated using TDValidator in ProSight Native (Proteinaceous, Inc. v1.0.25108; minimum score 0.50, terminal fragments; S/N cutoff 10, Max PPM Tolerance 10.00). Additional Proteoform Studio setting adjustments included decreasing the window width for fragmentation spectral averaging to 0.10 min (time to slide, 0.05 min). Analysis was performed in peak mode (i.e., peak picking in Proteoform Studio was performed to associate observed chromatogram peaks with target proteoform sequences). Only N- and C-terminal fragment ions were considered. The effect of increased stringency on sequence coverage was evaluated for composite spectra from multiple files by increasing the minimum number of observations of fragment ions from 1 to 3.^42^ For proton transfer charge reduction (PTCR) MS3 data, manual validation was performed using TDValidator in ProSight Native (Proteinaceous, Inc., v1.0.25254; minimum score 0.50, terminal fragments; S/N cutoff 10, Max PPM Tolerance 10.00), and automated data processing was performed in Proteoform Studio (v1.0.25206.1). Analysis in Proteoform Studio was performed in peak mode using fragment signal-to-noise ratio of 10. The effect of deconvolution settings on sequence coverage was evaluated for window mode “all”, sliding window mode with a window width of 0.10 min (time to slide of 0.05 min), and sliding window mode with a window width of 0.25 min (time to slide of 0.10 min). The effect of the minimum isotope fit score on sequence coverage was also evaluated for the three different deconvolution settings.

### Manual Validation

FreeStyle (Thermo Fisher) was used to average spectra across the base of each target (NIST Lc or NIST Fd) peak to maximize the number of averaged spectra and thus S/N. Fragments matched by TDValidator with S/N greater than or equal to 10 and isotope fit score greater than or equal to 0.5 were considered during the manual validation. These cutoffs were empirically established based on previous in-house analyses that indicated that fragment ions with lower S/N or lower fit scores were unlikely to be accepted. Multiple factors were considered for judging fragment ion assignments, including:

i. presence of abundant isotopologue peaks (in general requiring ≥3 for 3+ and higher charged ions and ≥2 for *z* = +1 or +2)
ii. mass error within 5 ppm of the average error for all assigned fragments
iii. qualitative visual consistency in *m*/*z* position of observed versus theoretical isotopologues
iv. qualitative visual distinction between matched peaks and surrounding noise signal, regardless of the level of the software-determined noise band.

In cases in which multiple fragments were assigned to the same set of peaks, only one match was retained. Previous in-house analyses also indicated that a score threshold of approximately 0.62 or 0.65 generally separates assignments more likely to be false versus assignments more likely to be acceptable for MS2 data, and in a number of cases, the fit score combined with visual comparison of observed versus theoretical isotopologue distributions and level of surrounding noise signal allows for clear determination of poor versus high-quality fragment assignments. Deviation from Gaussian peak distribution shape was not generally taken as the reason for rejecting a match as long as isotopologue peaks were present. For cases in which the validity of the match was more difficult to discern, the presence of other charge states of the given fragment was taken as evidence for the validity of the match, whereas an S/N closer to the minimum threshold was taken as evidence against the match.

## Results and Discussion

### Automating Subunit Characterization

With the goal of creating a single software solution to facilitate routine top-down and middle-down proteoform analyses, we present Proteoform Studio as a platform providing an integrated workflow for mass deconvolution, mass component identification, proteoform characterization, and fragmentation target selection for subsequent acquisition (Figure 1). Sliding window deconvolution for mass component creation was used to accurately determine the mass and retention times for the Lc and Fd subunits from NIST, trastuzumab, and SILu Lite antibodies (Table S1). From the mass components, acquisition charge state targets were readily determined from detected charge state distributions (Figure S1). Charge states from the precursor masses were ranked by intensity in Proteoform Studio and selected based on the approach of Kline et al. in which higher charge states were selected for ETD and EThcD fragmentation and lower charge states were selected for CID and HCD fragmentation. These selections were made on the basis that higher charge density would afford increased sequence coverage in electron-based fragmentation methods, whereas lower charge states would afford increased sequence coverage for the collisional dissociation methods.^43–45^ As a general rule of thumb, charge states with intensity of at least 75% of the most intense charge state were considered. For UVPD fragmentation, the most intense charge state <2000 *m*/*z* was selected, since sequence coverage has been reported to exhibit less variability with precursor charge density for UVPD fragmentation compared to other techniques.^45,46^ Proteoform Studio’s interface with the Thermo method editor allows charge state targets to be directly deployed to methods.

### Increasing Confidence in Automated Fragment Assignments

An important part of top- and middle-down MS data analysis is validation of detected fragment ions to reduce false positives. High confidence in fragment ion annotations is important for modification localization, especially when there is only a small number of fragment ions present to localize a modification. Ensuring that all fragment annotations are high quality is, therefore, vital for confident proteoform assignments. This situation becomes more likely as both the site of modification gets closer to the middle of the protein and the size of the molecule increases. Another important case exists when proteoforms of similar mass exhibit incomplete chromatographic separation, which increases the probability that different precursors will be co-isolated for fragmentation. As an example, for the simple mixture of 3 antibody standards used here, it was possible to target charge states for NIST subunits that did not overlap with charge states of either trastuzumab or SILu. However, even for this simple mixture, the elution profiles of NIST, trastuzumab, and SILu Lc subunits were observed to be overlapping (Figure S3). Accordingly, if a significantly more complex mixture was to be analyzed, such as an entire serum antibody repertoire, targeting subunit charge states that were completely distinct in retention time and *m*/*z* from any other species is expected to be exceedingly difficult. We therefore set out to find appropriate settings for automated analysis in Proteoform Studio that provide consistency with those results obtained from careful manual curation and validation. We initially determined sequence coverage for NIST Lc and Fd subunits both alone and in a simple antibody mixture for the five fragmentation techniques based on manually validated matching fragment ions in TDValidator.

Our criteria for manual validation are outlined in the Materials and Methods section. The criteria provide general guidelines for relatively conservative manual validation, though it is acknowledged that manual validation requires experience and expertise, which may lead to variability between different analysts and across laboratories. Validation results that consistently apply similar criteria and levels of stringency, however, are expected to agree within approximately 5% overall sequence coverage. Furthermore, experimental objectives can dictate the appropriate level of stringency for manual validation. For example, a discovery-type experiment may use more relaxed criteria, whereas high-throughput, quality control, and quantitative experiments will likely use much more conservative criteria. In Figure S4, we provide several examples of fragment assignments with high fit scores that were rejected during manual validation, along with the rationale for the rejection. Differences in sequence coverage before manual validation (i.e., accepting all fragment assignments with S/N at least 10 and fit score at least 0.5) versus after manual validation for the data shown in Figure 2A are summarized in Figure S5.

**Figure 2:**
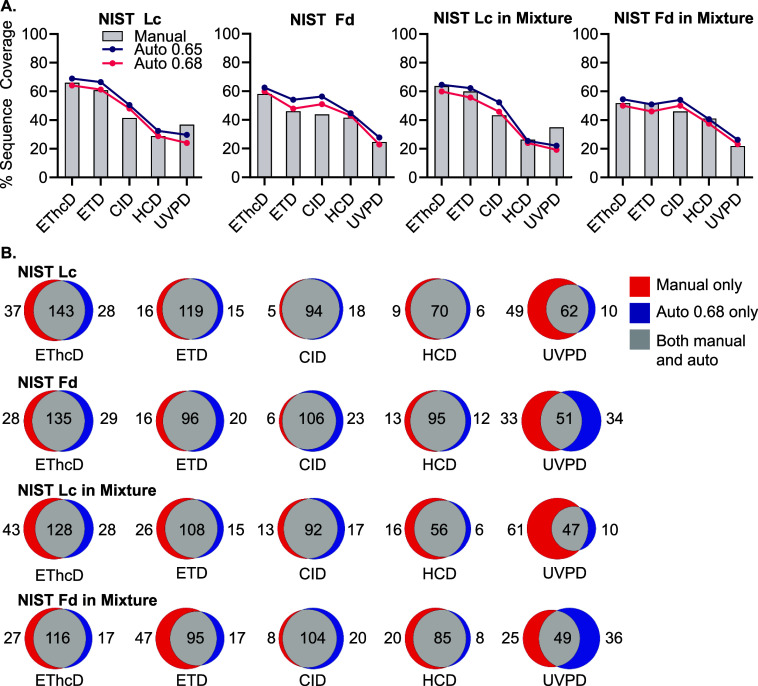
Sequence coverage comparison, automated analysis in Proteoform Studio versus manual validation. Subunits from above-hinge digestion and reduction of the NIST mAb standard were analyzed in a sample of NIST mAb alone as well as after being spiked into a simple mixture of NIST and two other antibody standards. Targeted MS2 with five different fragmentation techniques was used for each subunit. Sequence coverage was evaluated manually using TDValidator (“Manual”) and compared to results from automated processing in Proteoform Studio (“Auto”) using a window width of 0.1 min and time to slide of 0.05 min for fragmentation spectra averaging and minimum S/N = 10.0 for fragment ions. Minimum isotope fit score (here, 0.65 or 0.68) was adjusted to evaluate the setting providing consistency with manual validation results. (A) NIST Lc and Fd from NIST subunits alone and spiked into simple antibody mixture; (B) overlap in fragment ion assignment between manual validation results and automated processing in Proteoform Studio with minimum fit score of 0.68. Overlap in ion assignment considered ion name (i.e., backbone cleavage location without regard to charge and, in the case of UVPD, without regard to H transfer).

In Proteoform Studio, the minimum fragment signal-to-noise ratio (S/N) was increased to 10 and then different isotope fit score cutoffs were assessed for fragment assignment. A setting of 0.68 provided sequence coverage results that were generally consistent with sequence coverage determined from manual validation (Figure 2; Tables S2 and S3). Further workflow details are provided in Figure S6. With the exception of UVPD, fragment ion assignment was also relatively consistent for both manual and automated approaches (Figure 2B). UVPD has previously been shown to achieve high sequence coverage in middle-down analyses.^13,45–49^ However, the extremely dense spectra comprising many different types of fragment ions posed challenges during manual validation and were therefore not included in subsequent analyses in this study. It is also anticipated that optimization of the experimental settings would improve the UVPD sequence coverages observed here.

### Increasing Coverage through Aggregation of Fragmentation Results

After establishing targeted data analysis settings, we assessed the ability of Proteoform Studio to automatically aggregate fragmentation results from multiple files. We systematically evaluated how aggregation of results from multiple fragmentation techniques led to increases in the NIST Lc and Fd sequence coverage. Results based on 3 replicate MS2 injections of NIST subunits in a simple antibody mixture for five fragmentation modes are summarized in Figure 3 and Table S4. Further workflow details are provided in Figure S7. Combining results from EThcD and ETD with the complementary technique of HCD increased composite coverage to ≥77% for the Lc and ≥70% for the Fd for each of the 3 replicates. Combining all five fragmentation techniques yielded composite coverages exceeding 85% for the Lc and 80% or higher for the Fd. Next, the effect of increased stringency in fragment ion assignment was evaluated by increasing the minimum number of fragment observations to 3. For the purposes of this analysis, extensive optimization of fragmentation parameters was not performed; rather, the emphasis was on applying the automated aggregation capabilities of Proteoform Studio for rapid assessment of proteoform characterization results. Nonetheless, for the present data set, aggregation of results from five different fragmentation techniques increased coverage to more than 70% with one exception, even when increasing stringency of fragment ion assignment by setting the minimum number of observations to 3 (Figure 3B). With the exception of the first MS2 replicate for the Lc subunit when setting minimum observations to 3, the consistency of the trendlines in Figure 3A,B suggest that the workflow yielded reproducible results in terms of aggregate sequence coverage for 3 replicates acquired across 2 different dates of analysis (i.e., replicates 2 and 3 for ETD, HCD, UVPD, and CID were acquired on a different date from the other injections shown). The outlier MS2 replicate for the Lc subunit had noticeably lower TIC values, which likely contributed to the lower observed sequence coverage due to less signal for lower intensity fragment ions to rise above the S/N cutoff (Table S5).

**Figure 3:**
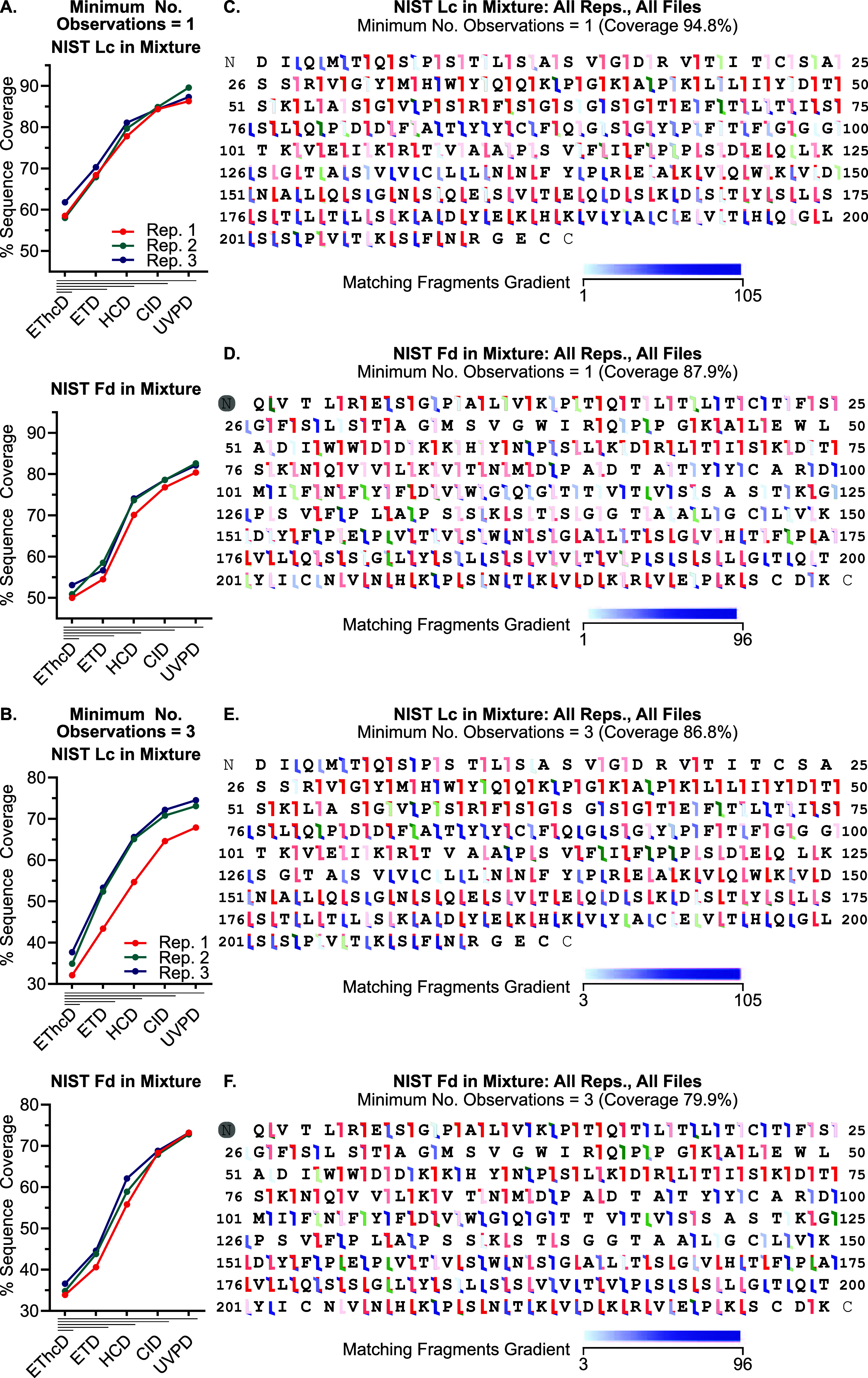
Increase in composite sequence coverage with different fragmentation modes across additional files. Sequence coverage observed from Proteoform Studio targeted analysis workflow using a sliding window width of 0.1 min and a minimum isotope fit score of 0.68. First, analysis was performed of an EThcD file individually, followed by the composite coverage from combining results with ETD and then combining HCD, CID, and UVPD. The lines under the *X* axis indicate the different fragmentation techniques for which results were aggregated for each sequence coverage output. (A) Minimum number of fragment observations set to 1. (B) Minimum number of observations increased to 3 for increased stringency. Also shown are composite fragment maps for all five files from all three replicates for (C) NIST Lc, minimum number of observations set to 1; (D) NIST Fd, minimum number of observations set to 1; (E) NIST Lc, minimum number of observations set to 3; (F) NIST Fd, minimum number of observations set to 3. In (C–F), fragment gradient color intensity scales as the log_2_ of the number of fragment observations.

### Optimizing Fragmentation Parameters and Charge State Targets

The preliminary data set above confirmed that combining multiple fragmentation techniques improves proteoform characterization by increasing sequence coverage. Next, EThcD, ETD, and HCD fragmentation settings were optimized for the analysis of NIST Lc and Fd subunits in the simple antibody mixture. Review of the fragmentation spectra and results in Proteoform Studio indicated substantial residual precursor signal in the HCD fragmentation data. Generally, an optimal HCD fragmentation energy leaves very little remaining precursor. Conversely, EThcD and ETD spectra and results indicated overdepletion of precursor signal and limited detection of higher mass fragment ions. With the goal of increasing sequence coverage via detection of larger fragment ions, HCD collision energy was increased and ETD and EThcD reaction time decreased. Further, higher charge states were targeted for ETD and EThcD given the higher efficiency of these techniques for higher-charge density precursors.^50^ Charge states were selected that were higher in charge while maintaining a relatively high intensity (>75%) compared to the most abundant charge state. It should also be noted that estimated on-column load (not accounting for loss during sample preparation and desalting) was increased from ∼0.75 μg to ∼1 μg for the optimized experiments as an additional means of improving proteoform characterization by increasing initial precursor signal (Amicon filters rather than Zeba columns were used for desalting in the optimized experiments, allowing for sample concentration). Characterization results were thus expected to reflect combined effects from optimization of fragmentation parameters and estimated increases in amount of analyte injected. Fragment maps comparing composite sequence coverage for EThcD, ETD, and HCD fragmentation for the preliminary versus optimized experiments are shown in Figure 4A,B and Figure S8. The composite fragment maps automatically generated by Proteoform Studio allow for rapid visual confirmation of increased sequence coverage. For the Lc subunit, coverage increased from 55.2% to 74.5% for the preliminary versus optimized data sets, respectively, when requiring a minimum number of fragment observations of 3. For the Fd subunit, coverage increased from 55.8% to 69.2% for the preliminary versus optimized data sets, respectively, when requiring a minimum number of fragment observations of 3. The increased coverage appears to arise in part from the observation of certain larger fragment ions for the optimized experiment (for example, considering residues 124–137 of the Lc subunit and residues 93–100, 113–120, and 133–140 of the Fd). While distributions of fragment ion masses were not significantly different for preliminary versus optimized settings (unpaired nonparametric Kolmogorov–Smirnov test, 2-tailed *p*-value 0.41 [Lc] and 0.95 [Fd]), qualitative inspection of fragment ion mass distributions indicates that additional fragment ions with masses between ∼8,000 and 10 000 Da were observed for the Lc subunit and near 10 000, 13 000, and 15 000 Da for the Fd subunit when using the optimized settings (Figure S9).

**Figure 4:**
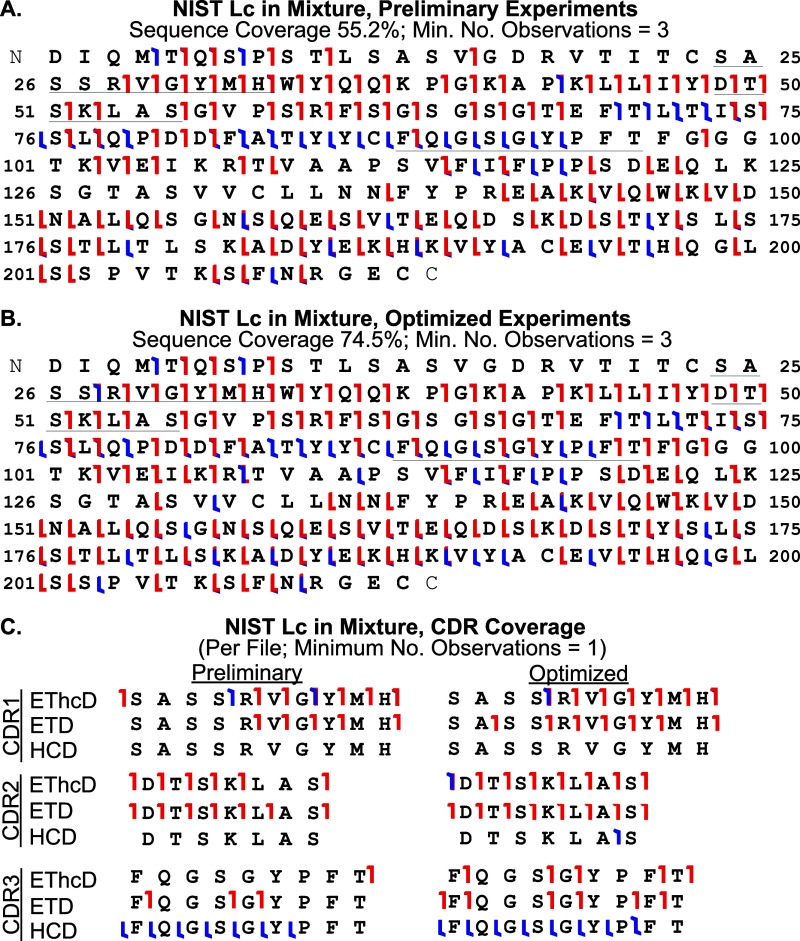
Aggregate fragment maps, preliminary and optimized experiments, and NIST Lc subunit. Fragment support for NIST Lc subunit spiked into a simple antibody mixture was evaluated for EThcD, ETD, and HCD fragmentation using two different sets of parameters. Preliminary experiments used a 6 ms ETD reaction time for ETD and EThcD and 30% NCE for HCD for analysis of NIST Lc (A). Optimized experiments used estimated higher on-column load as well as 5 ms ETD reaction time and higher charge state targets for ETD and EThcD, as well as 35% NCE for HCD for analysis of NIST Lc (B). Complementarity-determining regions (CDRs) are underlined.^52^ The increased coverage observed from combining results from different fragmentation techniques is apparent from considering fragment support for the CDR regions from each technique evaluated separately (C).

While the increase in coverage observed for the optimized versus preliminary MS2 experiments for the overall Lc and Fd sequences did not necessarily extend to the complementarity determining regions (CDRs), comparison of fragment support for each technique separately confirms the improved characterization provided by aggregation of different techniques (Figure 4C; Figure S8C). For the Lc, EThcD fragmentation using the optimized settings provided complete coverage of CDR2 as well as C-terminal residues of CDR1 yet sparse support for CDR3. However, although only a single fragment was matched to CDR2 using optimized HCD settings, HCD afforded nearly complete coverage of CDR3. A similar trend was observed for the Fd in that optimized EThcD settings supported coverage of the CDR2 region, whereas HCD fragmentation supported complete coverage of CDR3.^52^ Taken together, this specific example supports the idea that, even for potentially suboptimal fragmentation settings, as in the preliminary experiments, rapid and automatic aggregation of results from multiple files/multiple fragmentation techniques efficiently improves proteoform characterization.

### Effect of Sliding Window for Fragmentation Analysis

To evaluate the effect of implementing sliding window deconvolution for fragmentation data, as was done for the previous analyses, the analysis shown in Figure 3A,B was repeated using window mode “all” in the peak mode of Proteoform Studio. In this case, rather than considering multiple spectra per proteoform (generated from averaging spectra within each of a series of overlapping windows across the proteoform chromatographic elution peak), all spectra across the peak are averaged to generate one spectrum for analysis. Unlike with the sliding window mode, a single file analyzed in window mode “all” with a single averaged spectrum per proteoform (in peaks mode) contributes at most one fragment observation. In contrast, in sliding window mode, multiple observations of a given fragment are possible from a single file, assuming that fragment is observed in multiple windows. This difference is reflected in the results summarized in Figure S10, which depicts the average composite sequence coverage for each of the 3 replicate sets of 5 files, processed as per Figure 3A,B, for both sliding window deconvolution (window width 0.10 min; time to slide 0.05 min) and window mode all. Average coverage is comparable for a minimum number of observations of 1 but decreases dramatically for a minimum number of observations of 3, likely due to the reasons outlined above. Supporting data are summarized in Tables S4 and S6.

### Impact of Using a Minimum Number of Observations

To further assess the effect of window mode and number of observations on coverage and detection of false positives, the effect of increasing the minimum number of observations on the −log_10_(composite *P*-score) calculated by Proteoform Studio was evaluated by considering all files (i.e., all fragmentation techniques for all replicates). For window mode “all”, −log_10_(composite *P*-score) increases when the minimum number of observations is increased from 1 to 2 but decreases upon further increasing the minimum number of observations to 3. In contrast, −log_10_(composite *P*-score) consistently increases (i.e., composite *P*-score consistently decreases) as the minimum number of observations is increased from 1 to 3 for sliding window mode. Next, the analysis of Figure 3A,B was repeated except, rather than considering each replicate separately, in the initial step, all 3 EThcD files were processed, followed by adding all 3 ETD files and then all 3 HCD, CID, and UVPD files in turn. When considering all replicates for each fragmentation technique in each aggregation step, sequence coverage was relatively similar for each window mode, especially upon aggregation of results from multiple fragmentation techniques. Results are summarized in Figure S11 and Tables S7 and S8.

Results were consistent with the idea that combining sliding window mode with a requirement for multiple observations of each fragment may contribute to reducing false positive fragment assignments while retaining “true” assignments, since the *P*-score takes into account both matching fragments and total number of fragments. However, further scrutiny of fragment ion assignment suggests that, for the data set and settings used here, the key advantage of sliding window mode is increased sensitivity in matching fragment ions. In contrast, the window mode “all” appeared to provide slightly higher accuracy at the cost of omitting certain “true” assignments. Specifically, when ion assignments from manual validation were defined as ground truth, sliding window mode yielded a total of 333 ions for the NIST Lc with at least 3 observations, 90.4% of which were also accepted during manual validation, whereas window mode “all” yielded a total of 308 ions with 93.5% also accepted during manual validation. For the NIST Fd, sliding window mode yielded a total of 357 ions with at least 3 observations, 88.2% of which were also accepted during manual validation, whereas window mode “all” yielded a total of 316 ions with 93.0% also accepted during manual validation. Further, for the NIST Lc, when setting the minimum number of observations to 3, the sliding window mode retained 45 ions not retained by window mode “all”, 28 of which were also retained for manual validation. For the NIST Fd, sliding window mode retained 55 ions not retained by window mode “all”, 34 of which were also retained for manual validation. Overlap in ion assignment considered ion name (i.e., backbone cleavage location without regard to charge); though in the case of UVPD analysis, ions with and without hydrogen transfer were treated as distinct. Considering results of the ion overlap analysis, the improved composite *P*-score observed for sliding window mode versus window mode “all” when setting the minimum number of observations to 3 may reflect higher sensitivity in terms of retaining “true” assignments for sliding window mode (Figure S11A). With a cost to sensitivity, though, an increase in accuracy may be achieved with window mode “all”, which may be rationalized by the more stringent requirement that an ion be observed in 3 files (rather than, for example, 3 different subsets of spectra for a single file) when setting minimum observations to 3 in Proteoform Studio for this mode.

### Assessing Internal Ions to Increase Sequence Coverage

Matching internal ions in middle-down and top-down data analysis has been shown to significantly increase sequence coverage.^53,54^ Proteoform Studio allows for the assignment of internal ions by generating theoretical isotopic distributions of all possible internal ions. The software also has two sets of isotopic fitting parameters, one for terminal ions and one for internal ions, such that internal ion assessments can be performed with a higher stringency to limit false positives. We briefly looked at the impact of including internal ions during the analysis of the HCD data set (see the Internal Fragment Analysis section of the Supporting Information, including Figures S12–S15 and Tables S10–S13, for a more detailed analysis). Through the use of the minimum number of observations concept, we were able to include only those internal ions occurring across all 3 replicates (Figures S16 and S17). The data show compelling evidence of internal ions filling in gaps in terminal ion coverage, such as the 10 internal ions filling in N-terminal cleavage sites of NIST Fd between residues 25 and 50 that did not have associated terminal ion cleavages. Internal ions could be used in future studies to supplement terminal fragment ions to localize sites of modifications or as diagnostic ions for proteoforms with poorly characterized regions. However, given the potential for ambiguous fragment ion assignments and increased chance of false positive matches, especially for techniques that generate a wider array of fragment types, we recommend exercising caution when including internal fragment assignments.

### Characterizing Subunits in a Complex Matrix

Software and acquisition method settings informed by initial analyses of NIST subunits in a simple antibody mixture were next applied to the targeted analysis of NIST Lc and Fd spiked into a more complex plasma IgG background. As noted, not accounting for loss during sample preparation and desalting, ∼0.1 μg of NIST subunits spiked into ∼2.3 μg of plasma IgG was loaded on the column (ratio of IgG background to NIST of ∼20). Bondt et al. observed that the 30 most abundant IgG1 clones in human plasma corresponded to a median of 71.8% of calculated total IgG1.^55^ Assuming a plasma IgG1 concentration of 5.9 μg/μL (taking serum IgG concentration of 9.0 μg/μL as a reasonable estimate and taking IgG1 as ∼65% of this value), the total expected concentration of the 30 most abundant clones is ∼4.2 μg/μL or 0.14 μg/μL per clone (assuming equal concentrations, for purposes of this calculation).^56^ The expected ratio of IgG plasma background to an endogenous high abundance clone is then ∼64 (9.0 μg/μL/0.14 μg/μL). For the purposes of this pilot analysis, the estimated concentration of the NIST spike-in was ∼3-fold higher than expected physiologically relevant levels.

This experiment nonetheless provides a first step toward the application of the workflow to targeted IgG detection in complex samples. A reference elution profile for NIST Lc and Fd subunits alone, as well as ion chromatograms of NIST spiked into the plasma IgG background, are shown in Figure S18. Given the high concentration of the NIST spike-in, evidence for the NIST Lc and Fd subunits is observable even from the total ion chromatogram of NIST spiked into plasma and further supported by extracted ion chromatograms generated with the *m*/*z* values used for targeted analysis of the Lc and Fd. As shown in Figure S18B, isotope distributions are also qualitatively consistent for NIST subunits analyzed alone and spiked into plasma. Figure S18C,D shows EThcD fragment mass spectra (averaged across the peak elution profile) for the NIST Lc and Fd, respectively, spiked into the plasma IgG background acquired via a single MS2 injection. Optimized EThcD fragmentation settings discussed previously were selected based on the generally higher sequence coverage observed with these settings.

Though the amount of injected NIST relative to plasma IgG background is higher than physiologically relevant concentrations, the estimated on-column load of NIST subunits (∼0.1 μg) was still 2.5- to 5-fold lower than that used for analysis of either NIST alone (∼0.5 μg) or NIST spiked into a simple antibody mixture (∼0.25 μg of NIST subunits, preliminary experiments; ∼0.33 μg of NIST subunits, optimized experiments). Automated data processing via the targeted proteoform workflow in Proteoform Studio using the same settings as the initial experiments with a minimum isotope fit score of 0.68 and S/N threshold of 10 for fragment ions provided 68.9% and 50.4% sequence coverage for the Lc and Fd, respectively, for a single EThcD injection (Figure 5). Agreement between automated and manual approaches was within 6%, with higher coverage observed for the manual approach for the Fd (56.2% with manual compared to 50.4% with automated) versus lower coverage observed in the manual approach for the Lc (63.2% with manual compared to 68.9% with automated). Increasing the minimum number of observations from 1 to 2 yielded a greater reduction in false positive identifications (defined here as ions assigned in the automated analysis by Proteoform Studio that were not accepted during manual validation) compared to increasing the minimum isotope fit score from 0.68 to 0.70 (keeping the minimum number of observations set to 1). However, increasing the fit score provided slightly better consistency in overall percent sequence coverage between manual and automated results (66% and 46.9% for the Lc and Fd, respectively, upon increasing the minimum score versus 59.9% and 39.7% for the Lc and Fd, respectively, upon increasing the minimum number of observations).

**Figure 5:**
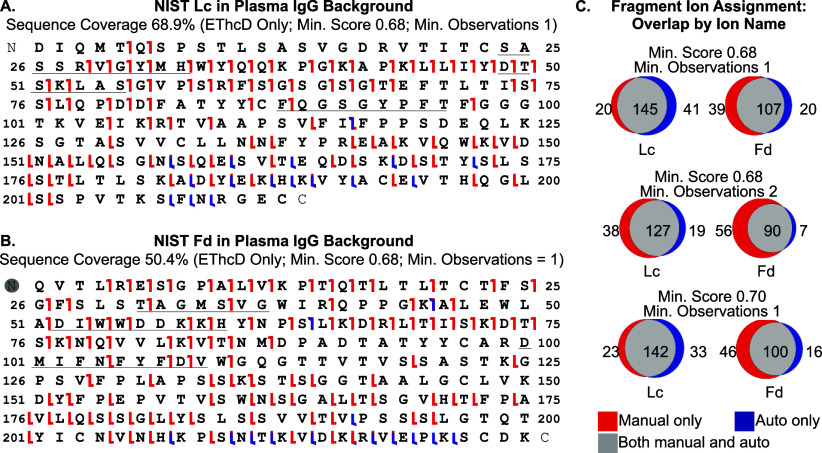
NIST mAb subunits spiked into plasma, MS2 fragment maps. Sequence coverage from targeted analysis (EThcD fragmentation) performed with Proteoform Studio (minimum isotope fit score of 0.68) for NIST subunits spiked into plasma IgG background for (A) NIST Lc and (B) NIST Fd. CDRs are underlined.^52^ (C) Overlap in fragment ion assignment between manual evaluation using TDValidator (“Manual”) and automated processing in Proteoform Studio (“Auto”). Proteoform Studio settings used a window width of 0.1 min and time to slide of 0.05 min for fragmentation spectra averaging and minimum S/N = 10.0 for fragments. Overlap in ion assignment considered the ion name (i.e., ion type and backbone cleavage location without regard to charge).

A key feature of Proteoform Studio for proteoform characterization is the ability to aggregate evidence from multiple files and multiple fragmentation modes and increase the stringency of fragment ion assignment by, as noted, increasing the minimum number of fragment observations or minimum isotope fit score. While these features are thus most relevant when considering multiple files and multiple fragmentation techniques for proteoform characterization, consideration of the single EThcD injection provides an example of the effect of each setting for tuning the stringency of ion assignment.^42^

### Improving Subunit Coverage with PTCR

Though previous analyses included MS2 data only, Proteoform Studio is also capable of handling PTCR MS3 data. By spreading out overlapping ion clusters in *m*/*z* space, PTCR has been shown to be advantageous for increasing the depth of coverage of complex mixtures of proteoforms and, in targeted experiments, for increasing the confidence of fragment ion assignment and localization of post-translational modifications as well as improving sequence coverage.^36,41,57–59^ Results from targeted analysis of the NIST mAb standard digested with IdeS and reduced via PTCR MS3 are summarized in the Supporting Information (Figure S19; Table S14). PTCR experiments used an increased on-column load (1.5 μg versus 0.5 μg) and increased source voltage (3800 V versus 3400 V) compared to the MS2 experiments to offset the expected effect of signal dilution into multiple lower charge state channels after PTCR. For PTCR MS3 data, the present analysis supports the use of a lower score threshold when using sliding window mode (0.62) versus window mode “all” (0.65) for sequence coverage results consistent with manual validation. This observation appears to contradict the expectation based on analysis of the MS2 data that sliding window mode increases the sensitivity of fragment ion assignment. However, as noted above, a possible rationale is that implementation of PTCR without ion parking is expected to result in signal dilution across multiple lower charge states for each product ion. Since window mode “all” averages all spectra across the proteoform elution peak, whereas sliding window mode averages multiple smaller subsets of spectra, the improved ion statistics and S/N obtained by averaging a higher number of spectra may be especially valuable for fragment ion assignment in PTCR data. As shown by Figure S19, using sliding window mode but with a wider averaging window (0.25 min) appears to provide a more balanced result compared to a narrow sliding window of 0.10 min, which may have resulted in too few spectra being averaged per window, and window mode “all”, which may have resulted in some loss of sensitivity by averaging too many scans, as observed for the MS2 data. For the wider window width, score thresholds of 0.65 or 0.68 (the latter judged to be most appropriate for the automated processing of MS2 data) are generally consistent with manual validation results in terms of percent sequence coverage. Overall, we recommend that the same general workflow steps for targeted proteoform analysis in Proteoform Studio (shown in Figure S6) can be used for both MS2 and PTCR MS3 data.

## Conclusion

Current available software solutions for top-down mass spectrometry proteoform sequencing have not been tailored to provide all of the necessary analysis steps for targeted characterization in a single software. As such, top-down experimentation remains difficult and time-consuming with researchers often relying on manual steps or home-built software solutions. Overall, these difficulties have led to much lower usage rates of top-down workflows compared to intact mass and peptide mapping workflows, despite the inherently information-rich data that can be obtained by top-down.

Here, we present a new software platform, Proteoform Studio, that provides a comprehensive informatics solution for automating targeted top-down and middle-down proteoform characterization. The intact mass deconvolution and targeted proteoform analysis provided in Proteoform Studio allowed for rapid automated intact mass profiling, proteoform fragmentation analysis, and data-guided optimization of a targeted method for characterization of NIST mAb Lc and Fd subunits spiked into a simple antibody mixture and a more complex plasma IgG background. As supported by the present analyses, Proteoform Studio’s ability to aggregate results from multiple files and fragmentation modes provides an important advance for proteoform characterization via top- and middle-down workflows. When results from multiple complementary fragmentation techniques are combined, significant improvements can be made in the overall proteoform characterization. Additionally, the data aggregation feature allows for composite sequence coverage calculations and fragment map creation to be automated and simplified. Furthermore, fragmentation parameters can be optimized on standard proteins and then applied again successfully in more complex sample settings. Tuning of the isotope fit score allowed for consistency between automated and manual results, providing confidence in results without the need for time-consuming manual validation. For larger data sets, the file aggregation feature of Proteoform Studio provides an additional control of false positives when aggregating many spectra and/or files together by allowing for filtering out of inconsistently observed fragment ions. Pilot analyses of NIST spiked into a plasma IgG background indicate that the identification of intact mass components and robust targeted proteoform characterization via Proteoform Studio may be directly applied to more complex workflows relevant for clinical proteomics and biopharmaceutical development. Overall, Proteoform Studio’s automated analysis streamlines top-down and middle-down workflows, paving the way for more accessible and comprehensive proteoform sequencing and characterization.

## Supplementary Material





## Data Availability

Raw data files are available at MassIVE under the repository number MSV000098545. Proteinaceous will provide free 60-day demo licenses of Proteoform Studio upon request through their Web site proteinaceous.net.

## Notes

Sample preparation and LC-MS/MS protocol details; supplementary figures, including screenshots of software workflows implemented, chromatographic elution profiles of antibody subunits, effect of software settings on composite sequence coverage, and PTCR MS3 sequence coverage; supplementary analysis of internal fragment ion assignment (DOCX)
Supplementary tables, including deconvolution results and supporting data tables for analysis of antibody subunit sequence coverage (XLSX)
