# Supplementary material for: Automating Middle-Down Mass Spectrometry Analysis for Extensive Antibody Characterization
Source: Anal Chem. 2026 Apr 13;98(16):11707–19. doi: 10.1021/acs.analchem.5c06408 (PMC13107428; doi:10.1021/acs.analchem.5c06408)
Supplement: Supplementary file 1 [file ac5c06408_si_001.docx]

**SUPPORTING INFORMATION**

**Automating Middle-Down Mass Spectrometry Analysis for Extensive Antibody Characterization**

Amy K. Carfagno^1^, Linda B. Lieu^2^, Jake T. Kline^1^, Luca Fornelli^1,2^*, Kenneth R. Durbin^3^*

*^1^School of Biological Sciences, University of Oklahoma, Norman, OK 73019, United States*

*^2^Department of Chemistry and Biochemistry, University of Oklahoma, Norman, OK 73019, United States*

*^3^Proteinaceous, Inc., Evanston, IL 60204, United States*

*To whom correspondence should be addressed:

Luca Fornelli, School of Biological Sciences, Richards Hall 411B, 730 Van Vleet Oval, Norman, OK 73019, United States. Phone: 405-325-1483; Fax: 405-325-6202; Email: luca.fornelli@ou.edu

Kenneth Durbin, Proteinaceous, Inc., P.O. Box 1839, Evanston, IL 60204, United States. Email: kdurbin@proteinaceous.net

**Abstract**

Supplementary information contained herein includes details of sample preparation and LC-MS/MS methods and screenshots of software workflows implemented for the analyses reported in the manuscript. Additional figures showing chromatographic elution profiles of antibody subunits, effect of software settings on composite sequence coverage, and PTCR MS3 sequence coverage results are also included. Finally, a detailed evaluation of internal fragment ions is provided to supplement the discussion in the main text regarding Proteoform Studio support for internal fragment analysis.

Table of Contents

[Supplementary Methods. 3](#_Toc226386392)

[Figure S1. Target Selection. 6](#_Toc226386393)

[Figure S2. Total Ion Chromatogram (TIC), NIST mAb Reduced and Digested with GingisKHAN. 7](#_Toc226386394)

[Figure S3. Elution Profiles of Antibody Standards. 8](#_Toc226386395)

[Figure S4. Manual Validation Examples and Associated Rationale. 9](#_Toc226386396)

[Figure S5. Comparison of Sequence Coverage before and after Manual Validation. 11](#_Toc226386397)

[Figure S6. Proteoform Studio Workflow Used for Comparison of Automated versus Manual Settings. 15](#_Toc226386398)

[Figure S7. Proteoform Studio Workflow Used for Cumulative Sequence Coverage Analysis Based on Aggregation of Multiple Files/Fragmentation Techniques. 17](#_Toc226386399)

[Figure S8. Aggregate Fragment Maps, Preliminary and Optimized Experiments, NIST Fd Subunit. 18](#_Toc226386400)

[Figure S9. Scatterplots of Assigned Fragment Ion Monoisotopic Masses for the Preliminary versus Optimized Datasets. 19](#_Toc226386401)

[Figure S10. Targeted Proteoform Analysis: Average Composite Sequence Coverage for Sliding Window versus “All” Window Mode. 20](#_Toc226386402)

[Figure S11. Composite P-Score and Sequence Coverage Comparison, Sliding Window versus “All” Window Mode, NIST in Mixture. 21](#_Toc226386403)

[Internal Fragment Analysis 22](#_Toc226386404)

[Table S9. Internal Ions Assigned and Accepted, Manual Validation 23](#_Toc226386405)

[Figure S12. Additional Cleavage Locations Identified Based on Manually Validated EThcD Internal Fragments. 23](#_Toc226386406)

[Figure S13. Additional Cleavage Locations Identified Based on Manually Validated HCD Internal Fragments. 24](#_Toc226386407)

[Table S10. Internal Ions Assigned, Proteoform Studio. 25](#_Toc226386408)

[Table S11. Internal Fragment Ion Contribution to Sequence Coverage, Manual Validation. 26](#_Toc226386409)

[Table S12. Internal Fragment Ion Contribution to Sequence Coverage, Automated Analysis in Proteoform Studio. 26](#_Toc226386410)

[Table S13. Internal Fragment Ion Contribution to Sequence Coverage, Automated Analysis in Proteoform Studio and Manual Curation Based on Mass Similarity. 26](#_Toc226386411)

[Figure S14. Internal Fragment Ion Assignment Evaluation in Proteoform Studio. 29](#_Toc226386412)

[Figure S15. Internal Fragment Ion Visualization in Proteoform Studio. 32](#_Toc226386413)

[Figure S16. Inclusion of Internal Ions for Analysis of HCD Fragmentation Data for NIST Lc Subunit. 33](#_Toc226386414)

[Figure S17. Inclusion of Internal Ions for Analysis of HCD Fragmentation Data for NIST Fd Subunit. 35](#_Toc226386415)

[Figure S18. NIST mAb Subunits Spiked into Plasma. 37](#_Toc226386416)

[Figure S19. Sequence Coverage Comparison, Automated Analysis in Proteoform Studio versus Manual Validation, Proton Transfer Charge Reduction (PTCR) MS3. 38](#_Toc226386417)

# Supplementary Methods.

*Antibody sample preparation.* Monoclonal antibody standards (trastuzumab, Millipore Sigma; SILu Lite MSQC4, Millipore Sigma; NIST SRM 8671, National Institute of Standards and Technology) were digested with GingisKHAN (Genovis, Kävlinge, Sweden) using a ratio of 2 units enzyme/μg antibody in 0.1 M Tris, pH 8, in the presence of 1 mM L-cysteine for 1 h at 37 °C on a thermal mixer set to 1000 rpm.^1^ Digestion was quenched by addition of 0.5% TFA (final concentration ~0.08% v/v) prior to performing reduction in 6.57 M guanidinium chloride (Thermo Scientific, Waltham, MA), 100 mM dithiothreitol (Fisher Scientific, Waltham, MA), and 100 mM tris(2-carboxyethyl)phosphine hydrochloride (GoldBio, St. Louis, MO) for 45 min at 50 °C. Desalting and buffer exchange was performed using either Zeba spin desalting columns (7K molecular weight cutoff [MWCO], Thermo Scientific) (preliminary experiments) or Amicon ultra centrifugal filters (10 kDa MWCO, Millipore Sigma, Burlington, MA) (optimized experiments). Sample preparation was performed immediately prior to analysis via liquid chromatography-tandem mass spectrometry (LC-MS/MS). For targeted analysis of NIST Lc and Fd subunits in the simple antibody mixture, equal amounts (assuming equivalent recovery during sample processing and desalting) of each of the NIST, trastuzumab, and SILu Lite preparations were combined. For targeted analysis of NIST Lc and Fd subunits in the plasma IgG background, 83.3 μL Protein G magnetic beads (Pierce, Thermo Scientific) in Tris-buffered saline (TBS; 25 mM TrisCl, 150 mM NaCl, pH 7.5) were used for IgG capture from 5 μL commercial human plasma (BioIVT, Westbury, NY) via mixing at room temperature for 1 h. After washing with TBS, captured IgGs were eluted with 0.1 M glycine (pH 2) and immediately neutralized to pH ~7-8 with 1 M Tris (pH 8.5), prior to performing digestion with 50 units FabRICATOR (IdeS; Genovis) for 0.5 h at 37 °C. The digestion was quenched and F(ab’)_2_ and Fc subunits were reduced using the same conditions as described for the antibody standard preparations prior to desalting and buffer exchange using Amicon ultra centrifugal filters (10 kDa). For proton transfer charge reduction (PTCR) MS3 experiments, NIST monoclonal antibody standard was digested with IdeS (FabRICATOR; Genovis, Kävlinge, Sweden) at a ratio of 1 unit enzyme/μg antibody in 50 mM Tris (pH 6.8) for 0.5 h at 37 °C on a thermal mixer set to 450 rpm. Digestion was quenched with 0.5% TFA and the sample reduced and desalted with a Zeba column as described for antibody sample preparation for the MS2 experiments.^2^

*LC-MS/MS analysis of antibody subunits.* Samples were separated using a MAbPac column (1 mm i.d. by 150 mm length, Thermo Scientific) with a Thermo Scientific Ultimate 3000 UHPLC system at a flow rate of 120 μL/min and column temperature of 70 °C. Mobile phase A (MPA) was 0.2% (v/v) formic acid (FA) in water; mobile phase B (MPB) was 0.2% (v/v) FA in acetonitrile. The 20-minute chromatographic method involved holding at 5% B for 2 min prior to rapidly ramping to 27% B over 0.5 min. The two-segment gradient involved increasing to 29% B over 4.5 min prior to increasing to 45% B over 6.5 min, prior to washing the column at 85% B and re-equilibrating at 5% B. Estimated load on column (not accounting for sample loss during preparation and desalting) was ~0.5 μg for analysis of NIST subunits alone; ~0.75 μg and ~1 μg total for analysis of NIST in simple antibody mixture for preliminary versus optimized experiments, respectively; and ~0.1 μg NIST in ~2.3 μg plasma IgG background. A Thermo Scientific Orbitrap Eclipse Tribrid was used for mass spectrometry analyses in intact protein mode (N_2_ pressure in ion routing multipole of 0.003 Torr) and source fragmentation of 15 V. To verify subunit intact mass and define targeted methods, broadband mass spectra (MS1) were acquired in the Orbitrap using resolution of 240,000 (at *m/z* 200) over a 500-4000 *m/z* window, with a maximum injection time of 500 ms, automatic gain control (AGC) target of 8e5 charges, and 5 microscans/mass spectrum. Targeted tandem mass spectrometry (MS2) experiments used quadrupole isolation (window width: 3 *m/z* units), with detection in the Orbitrap at 240,000 resolution (at *m/z* 200) over a 400-2000 *m/z* window using 500 ms maximum injection time, AGC target of 1e6, and 3 microscans/mass spectrum. For NIST subunits alone and NIST subunits in a simple antibody mixture, two sets of experiments (here referred to as “preliminary” and “optimized”) were used. Preliminary experiments included fragmentation via electron transfer dissociation (ETD) with 6 ms reaction time, electron transfer dissociation–higher-energy collisional dissociation (EThcD) using 6 ms ETD reaction time and 15 V supplemental activation energy, HCD with normalized collision energy (NCE]) set at 30%, 213 nm ultraviolet photodissociation (UVPD) with 25 ms activation time, and collision-induced dissociation (CID) with NCE set at 35%.^2, 3^ In the second set of experiments, fragmentation was performed via ETD (5 ms reaction time), EThcD (5 ms reaction time; 15 V supplemental activation energy), and HCD (NCE=35%), and higher charge state precursors were targeted for ETD and EThcD (for the Lc, +26 versus +25 in the “optimized” versus “preliminary” method; for the Fd, +25 versus +22 for the “optimized” versus “preliminary” method). Based on the higher sequence coverage observed for EThcD, EThcD settings from the optimized experiments were used for targeted analysis of NIST Lc and Fd subunits spiked into plasma. MS2 data was acquired in full profile mode. For evaluation of automated analysis of PTCR MS3 data via Proteoform Studio, targeted LC-MS/MS experiments of NIST Lc and Fd’ subunits were performed using EThcD, ETD, and HCD fragmentation followed by subjecting product ions to PTCR. The LC method and MS source settings were the same as those used for the MS2 experiments, except estimated on-column load was increased to 1.5 μg and source voltage was increased to 3800 V. Targeted PTCR MS3 experiments used quadrupole isolation (window width: 3 *m/z* units), with detection in the Orbitrap at 240,000 resolution (at *m/z* 200) over a 500-8000 *m/z* window using 500 ms maximum injection time, AGC target of 1e6, and 2 microscans/mass spectrum. Fragmentation was performed via ETD with 4 ms reaction time, EThcD using 4 ms reaction time and 15% supplemental activation energy, and HCD with NCE set at 35%. In each case, product ions were subsequently subjected to PTCR (reagent target 1e6) with 30 ms reaction time. For ETD and EThcD, the +26 charge state of both the Lc and the Fd’ were targeted, whereas for HCD, the +19 charge state of the Lc and +21 charge state of the Fd’ were targeted. PTCR MS3 data were acquired in full profile mode.^2^

**A.**


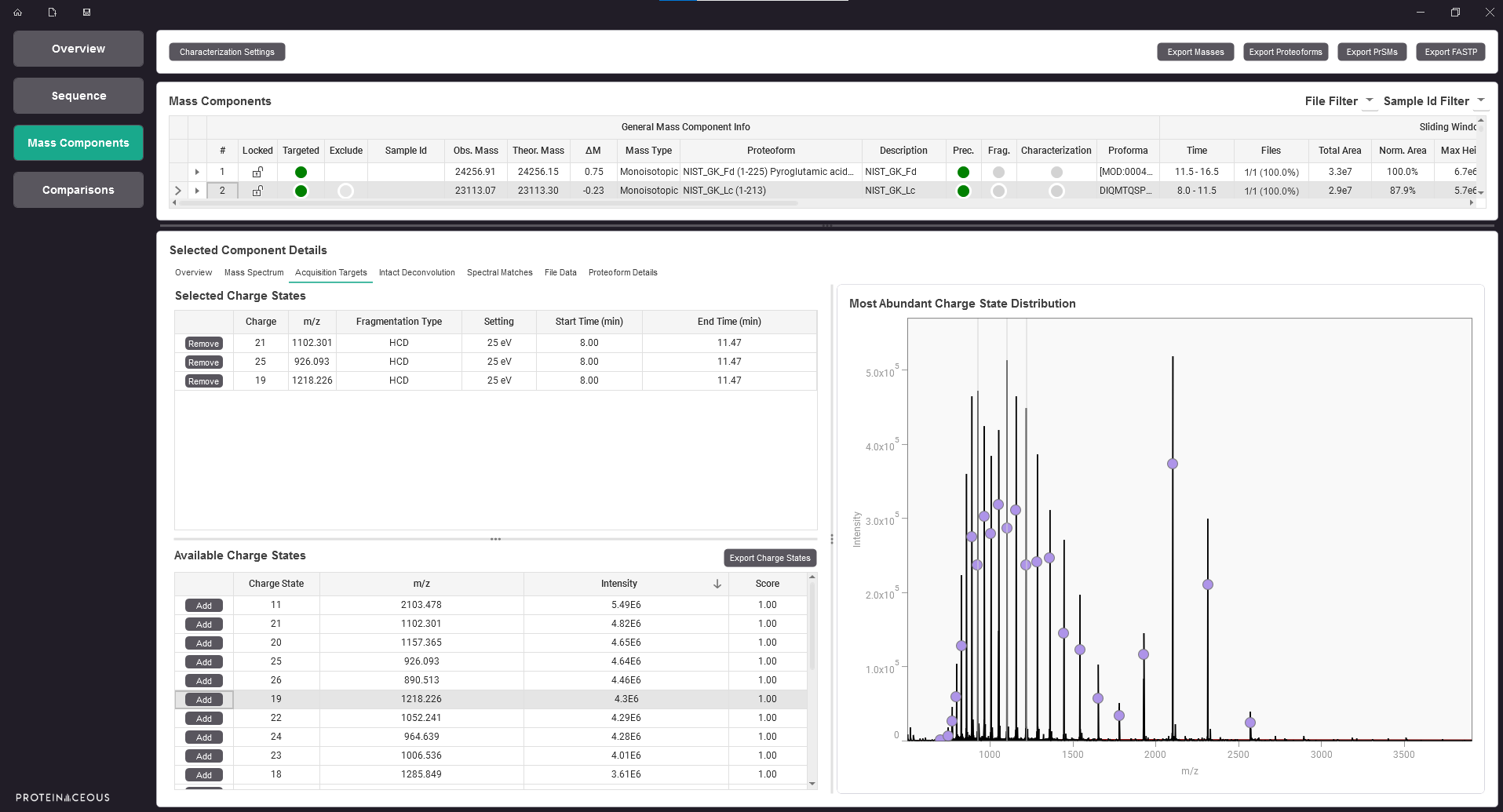


**B.**


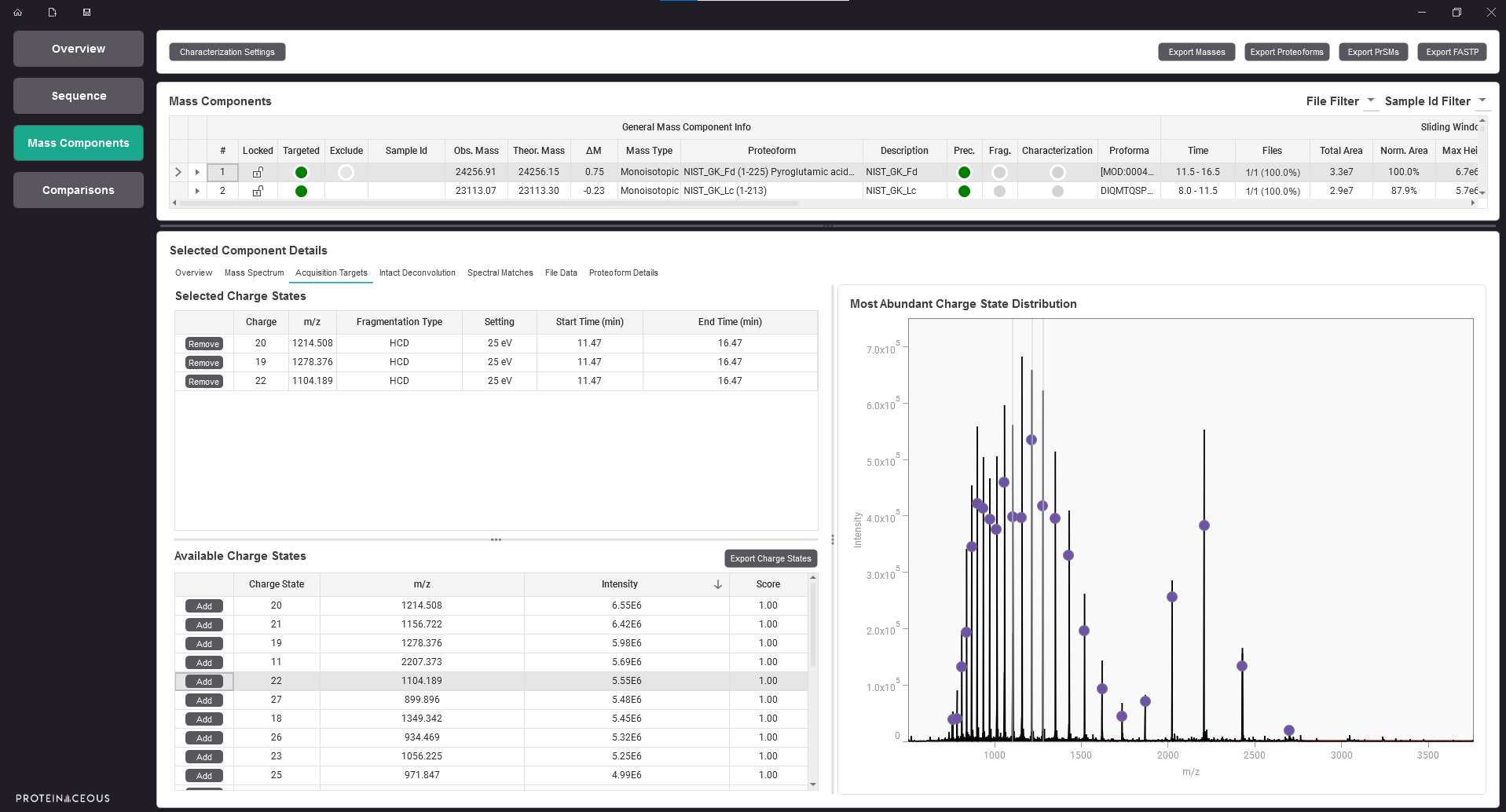


# Figure S1. Target Selection.

Proteoform Studio screenshots depicting target selection for NIST mAb Lc (A) and Fd (B) subunits. Scheduling was refined based on review of elution profiles in raw data.


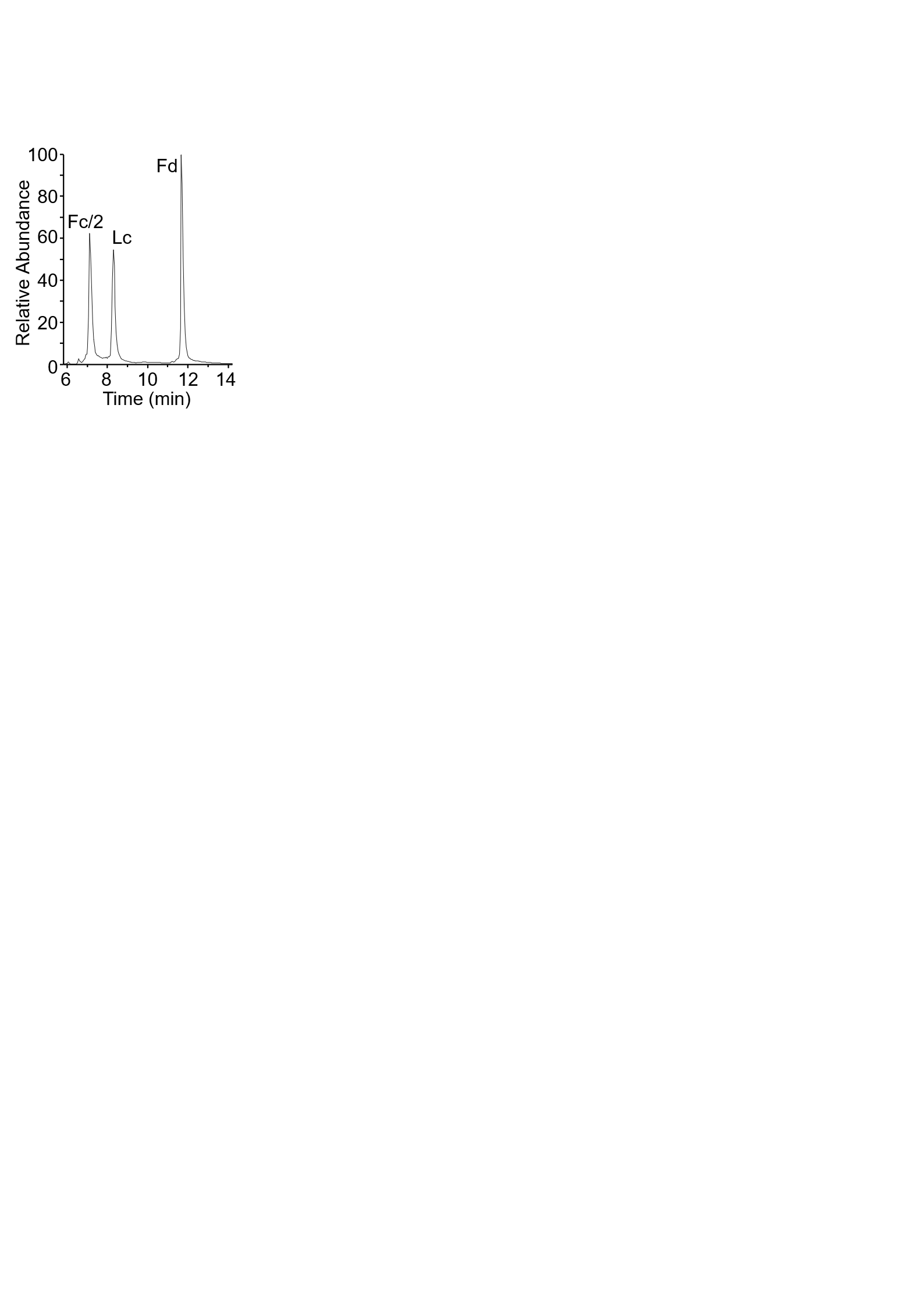


# Figure S2. Total Ion Chromatogram (TIC), NIST mAb Reduced and Digested with GingisKHAN.

TIC from MS1 acquisition (resolution 240,000 at *m/z* 200).

**
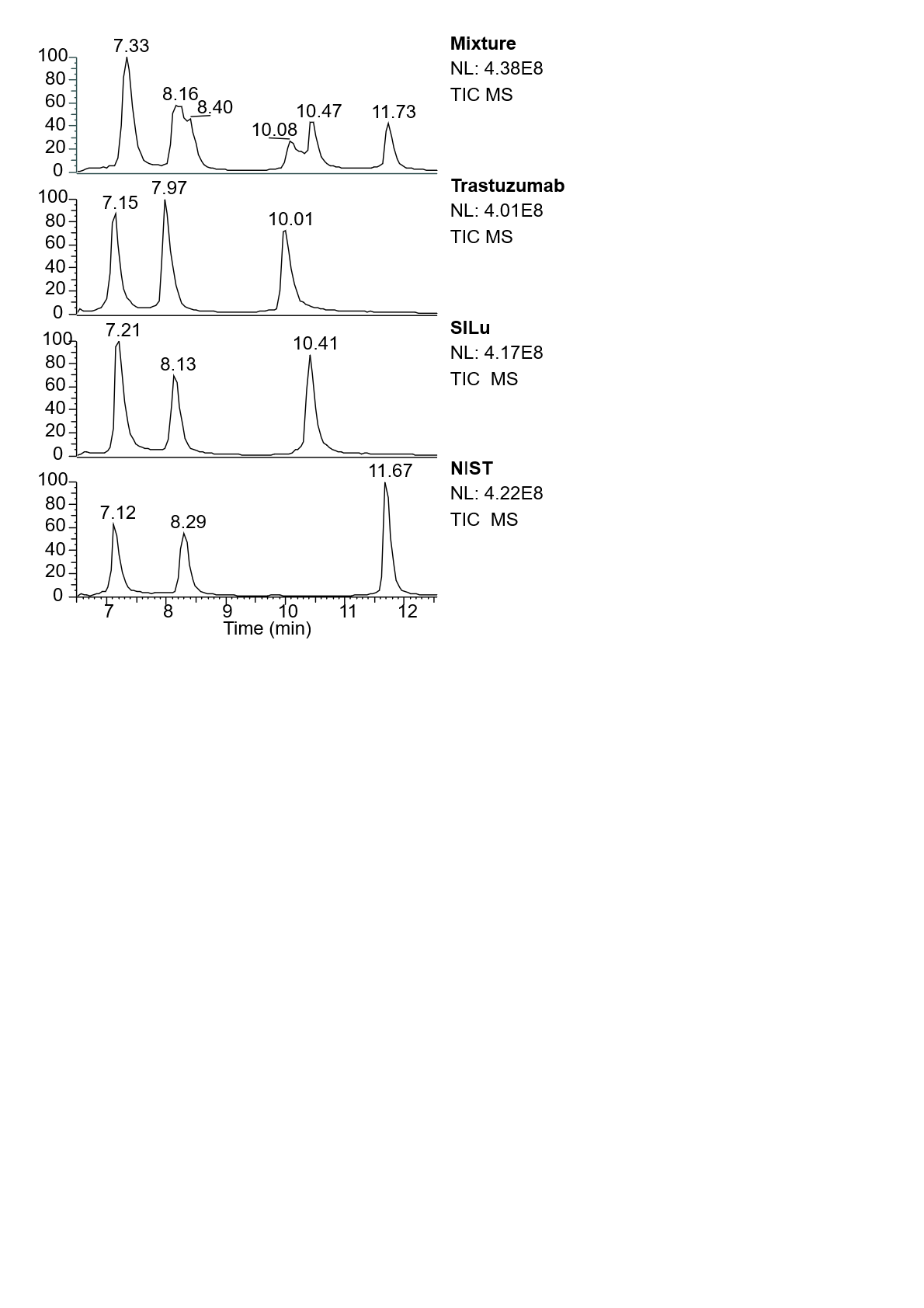
**

# Figure S3. Elution Profiles of Antibody Standards.

Elution profiles of mixture of digested and reduced antibody standards, as well as of each standard analyzed separately, showing overlap in Lc and Fd elution.


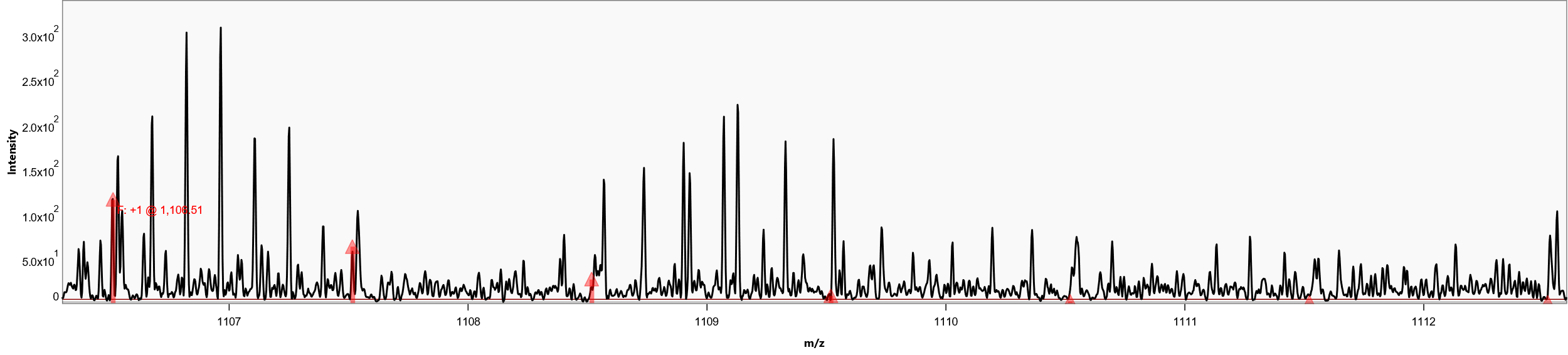
**A.**

**B.**

**
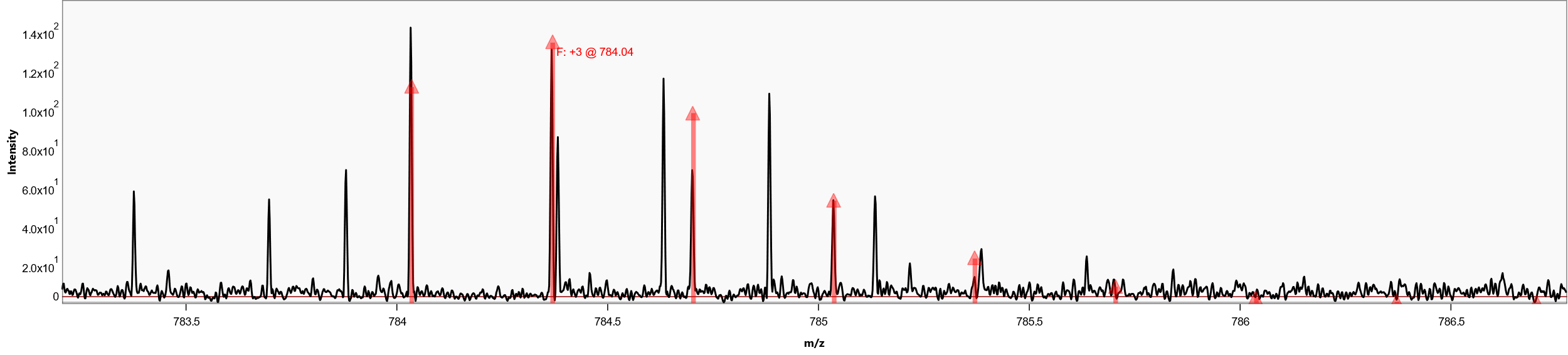
**

**C.**

**
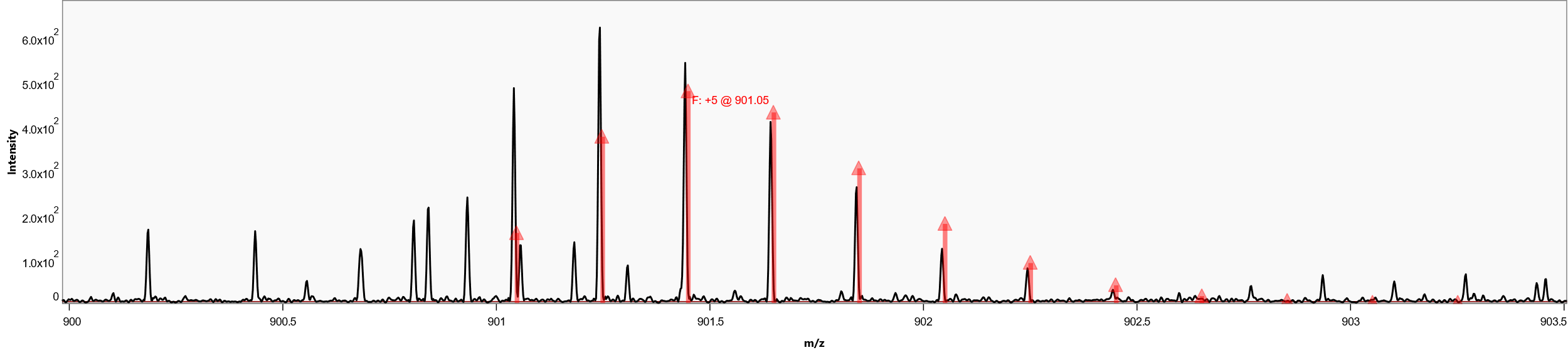
**

|  | **Active** | **Name** | **Theoretical Mass** | **ppm Error** | **Charge** | **Mono. *m/z*** | **S/N** | **Intensity** | **Score** |
| --- | --- | --- | --- | --- | --- | --- | --- | --- | --- |
| **A.** | False | C10 | 1,105.51 | -2.03 | 1 | 1,106.51 | 39.3 | 214 | 0.81 |
| **B.** | False | Y22 | 2,349.08 | -3.11 | 3 | 784.04 | 47.1 | 430 | 0.72 |
| **C.** | False | Y41 | 4,500.21 | -8.31 | 5 | 901.05 | 166.8 | 2,072 | 0.70 |

# Figure S4. Manual Validation Examples and Associated Rationale.

Selected fragment ion matches, NIST Lc, EThcD replicate 1, preliminary dataset. Manual validation performed using TDValidator in ProSight Native (v1.0.25108). Visualization of matches between theoretical and observed isotopologue distributions (A-C) exported as .XPS from TDValidator and converted to .SVG using CloudConvert (https://cloudconvert.com/xps-to-svg). Rationale for acceptance versus rejection of each match was as follows:

(A) Evidence supporting the validity of the match includes the high fit score (0.81), mass error (-2.03 ppm) consistent with average error for accepted fragments (-2.26 ppm), and presence of signal consistent with the 3 most intense theoretical isotopologue peaks. Evidence against the validity of the match includes the relatively lower S/N (29.3), low intensity (214), and similar intensity of local (putative) noise peaks. Using a relatively conservative approach for manual validation, the fragment was rejected.

(B) Evidence supporting the validity of the match includes the high fit score (0.72), mass error (-3.11) within 5 ppm of the average error for accepted fragments (-2.26 ppm) and observed signal consistent with the 4 most intense theoretical isotopologue peaks. Evidence against the validity of the match is the presence of a peak at *m/z* 783.6969 that differs from the peak at *m/z* 784.0329 by 0.3359 (determined using Thermo FreeStyle) and the inconsistency in the relative intensity of the two lowest *m/z* theoretical peaks versus the corresponding experimentally observed peaks, which may indicate misassignment of the signal as a Y22 ion. Using a relatively conservative approach for manual validation, the fragment was rejected.

(C) Evidence supporting the validity of the match includes the high fit score (0.70), high S/N (166.8), and high intensity (2,072). Evidence against the validity of the match is the high mass error (-8.31 ppm), which is more than 5 ppm from the average mass error for accepted fragments (-2.26 ppm). Further, the left-hand side of the observed signal appears to extend beyond the theoretical isotope distribution. Based on this inconsistency between theoretical and observed isotopologue distributions, along with the high mass error, the fragment was rejected.


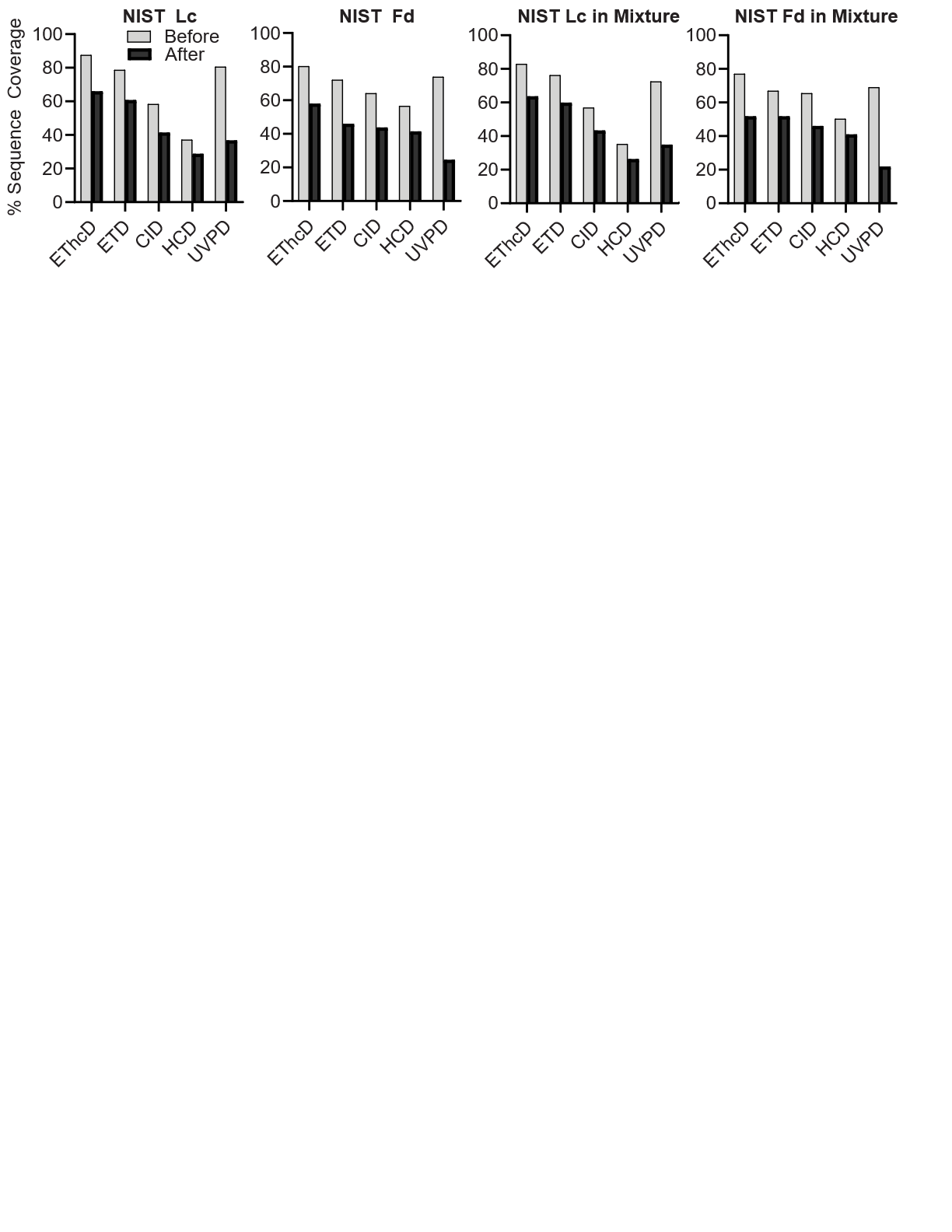


# Figure S5. Comparison of Sequence Coverage before and after Manual Validation.

Sequence coverage was evaluated manually using TDValidator, initially considering all fragment ion assignments with minimum S/N=10.0 and minimum fit score=0.50 (“Before”). Coverage after manual validation (“After”) is the same data as shown in the bar plot in **Figure 1A** and reflects ions accepted after review of factors including fit score, visual qualitative consistency between theoretical and observed isotopologue distributions, S/N, and mass error.

**A.**


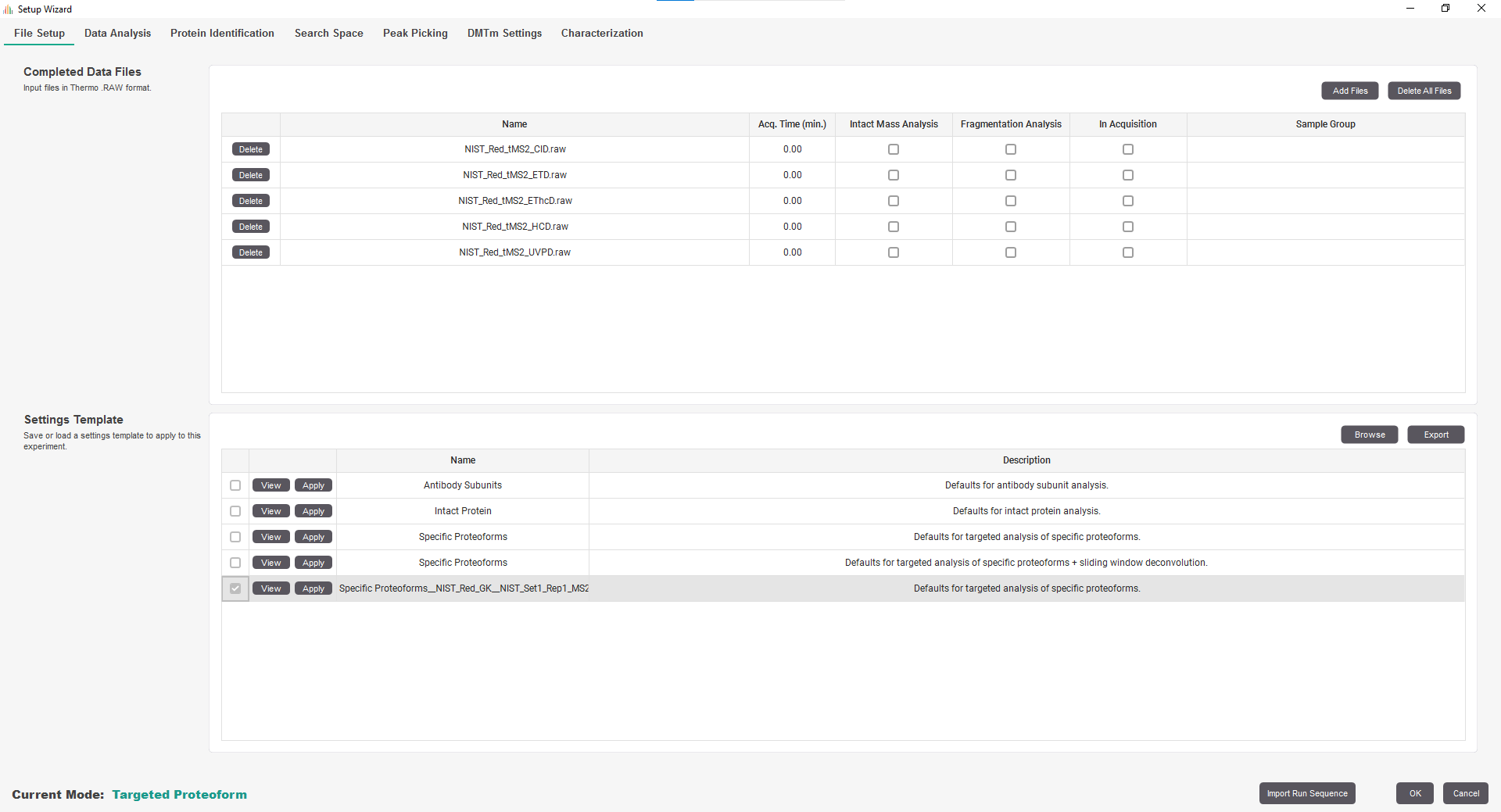


**B.**


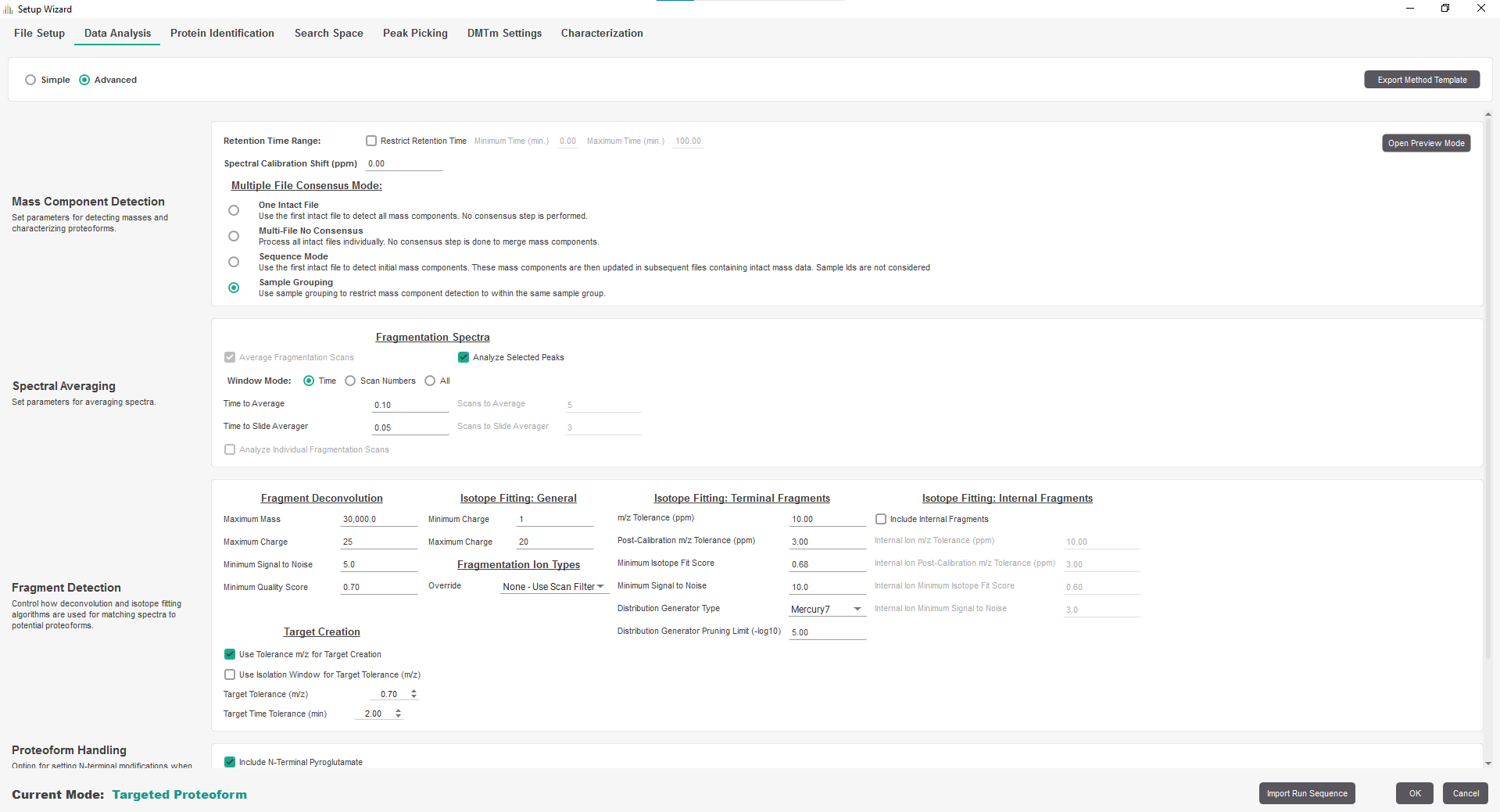


**C.**


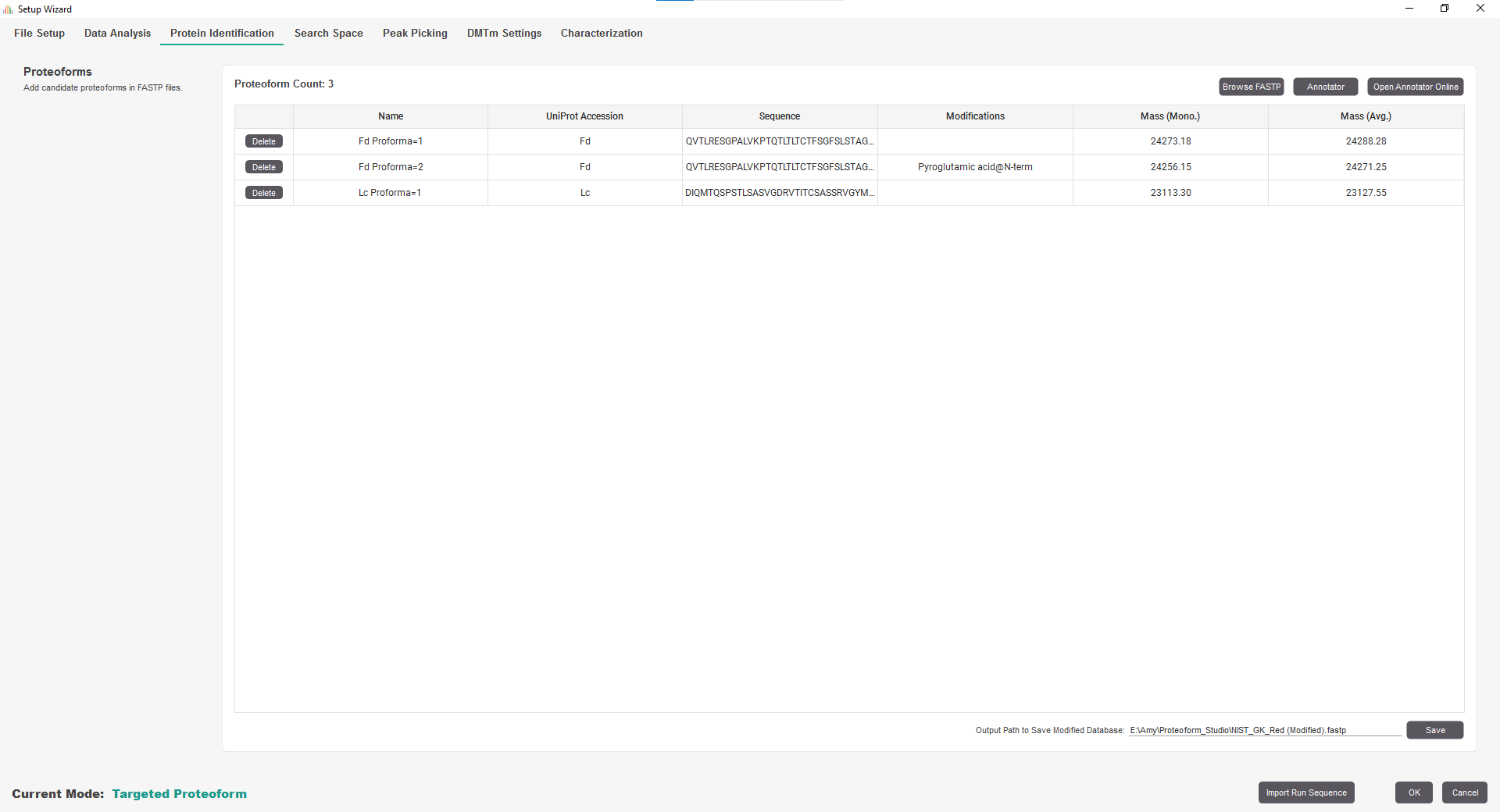


**D.**


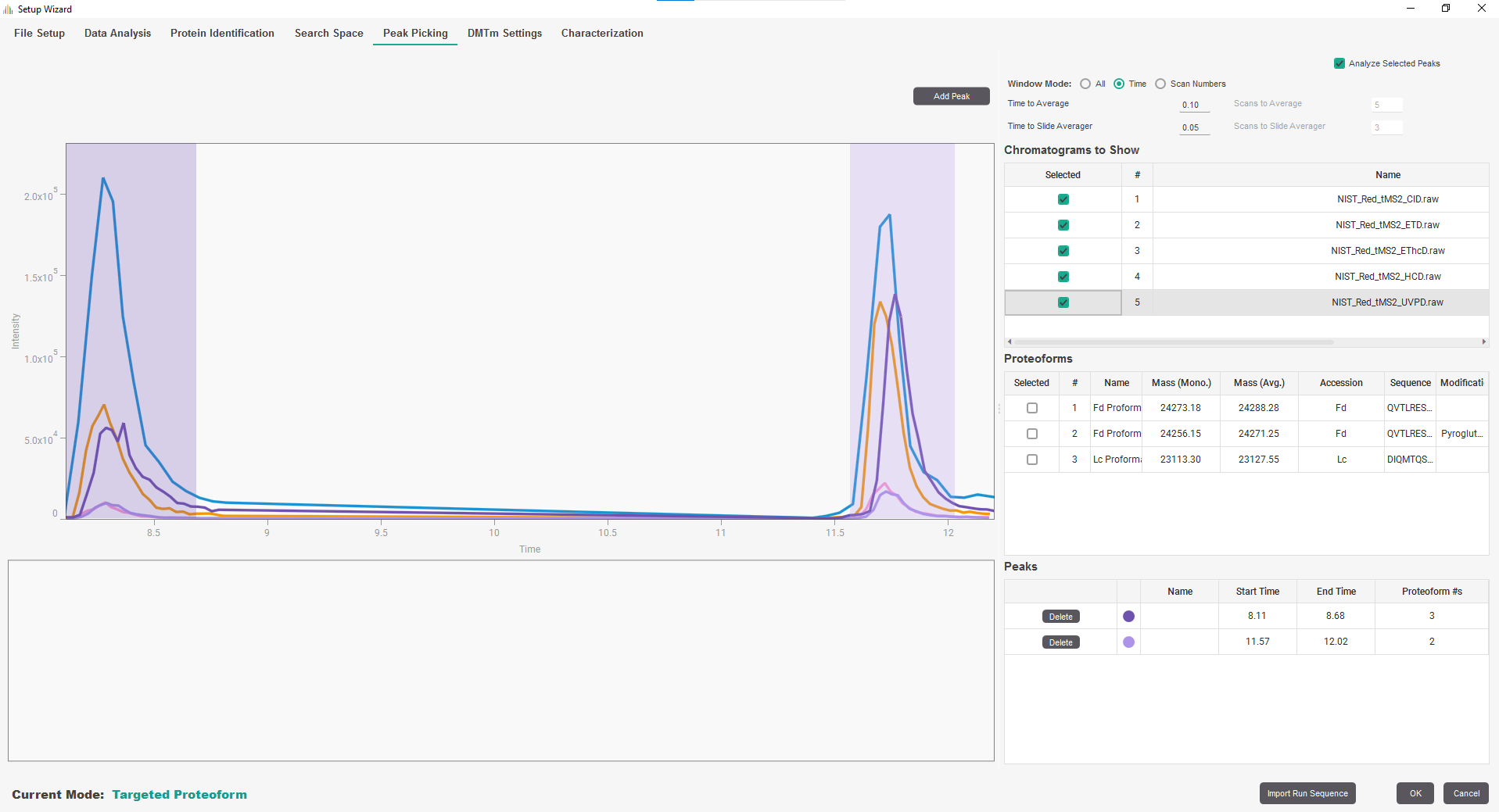


**E.**


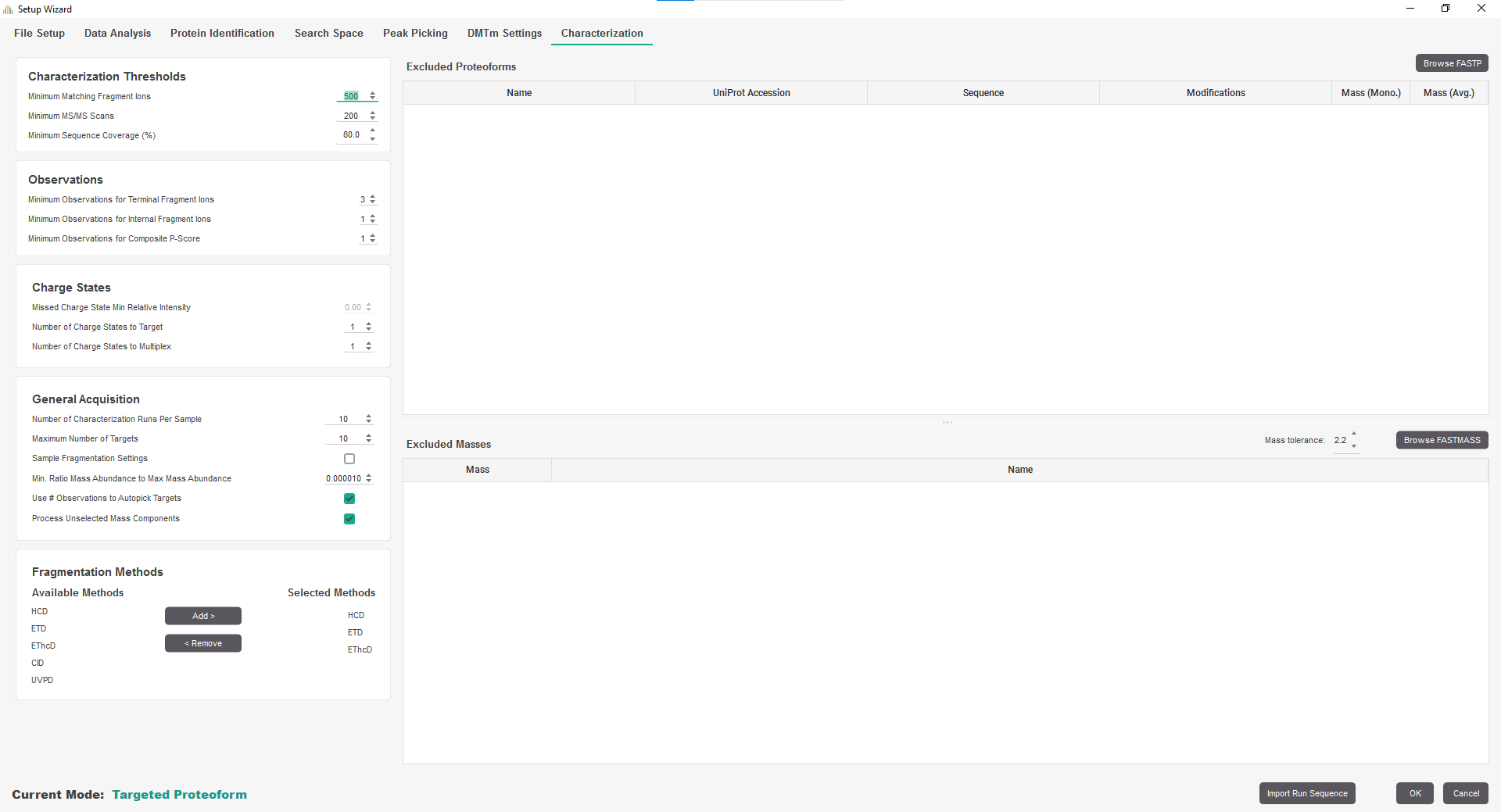


**F.**


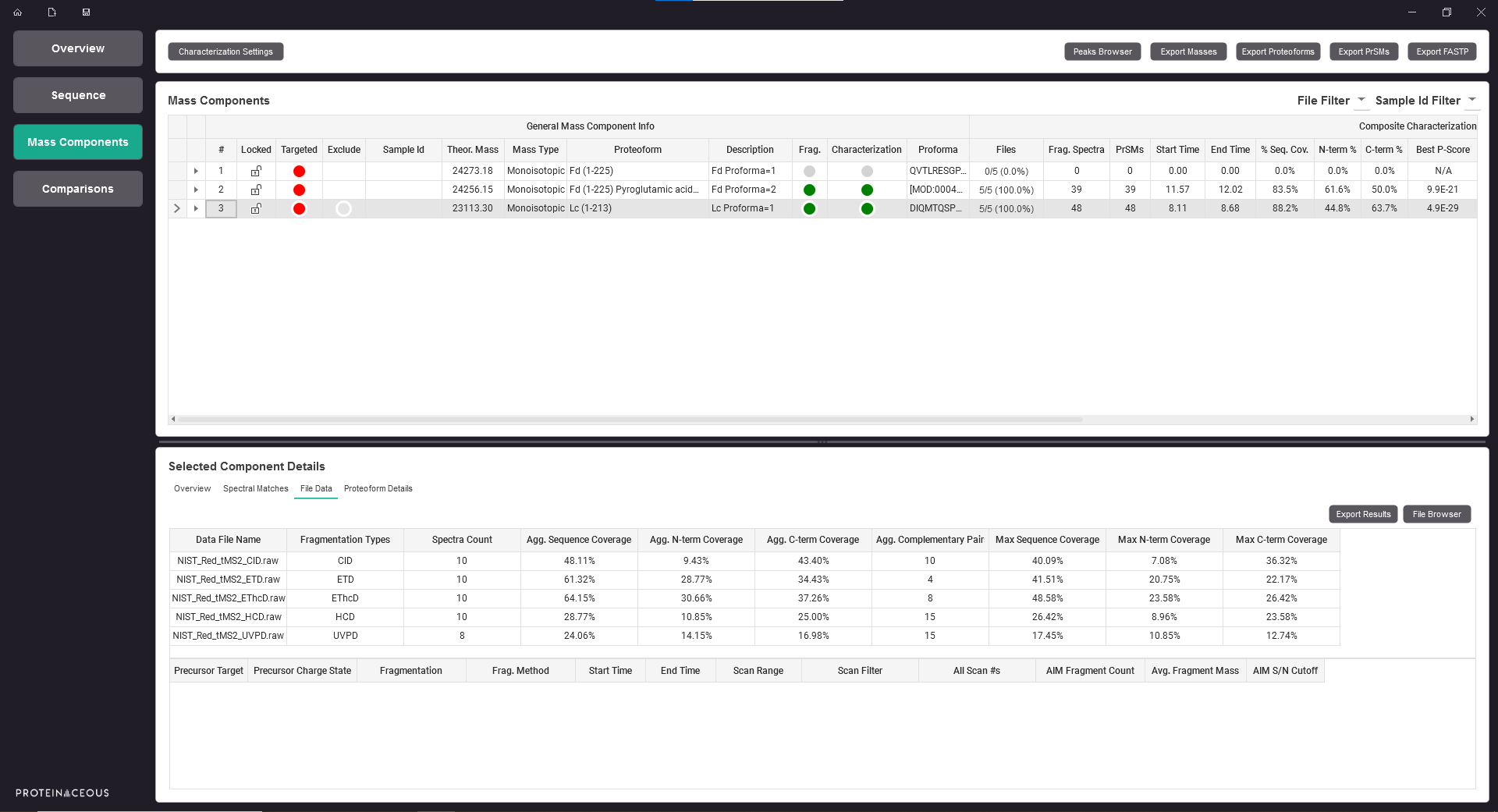


**G.**


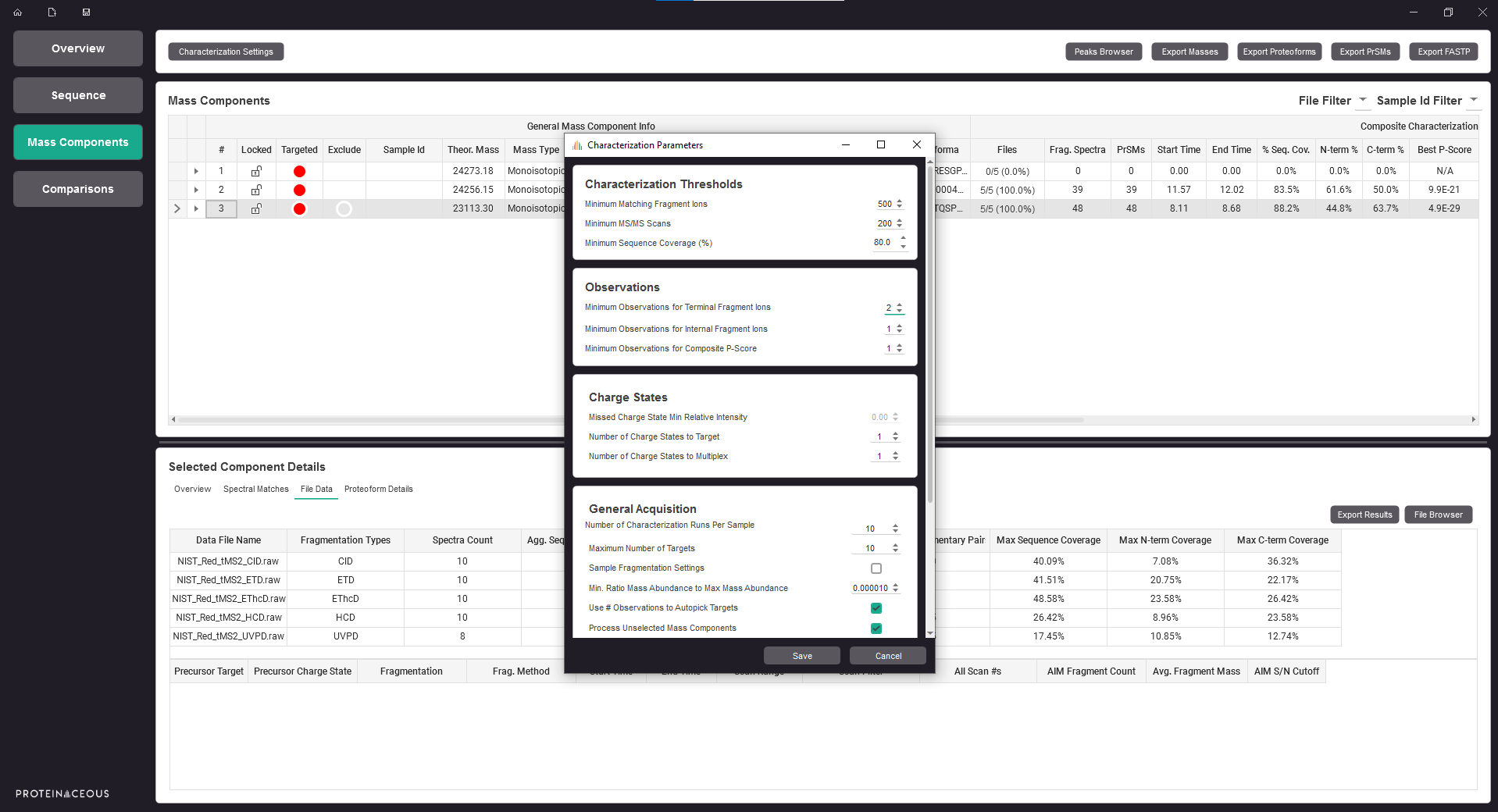


# Figure S6. Proteoform Studio Workflow Used for Comparison of Automated versus Manual Settings.

(A) File Setup tab: process 5 files from each dataset (first replicate per fragmentation mode, NIST mAb, targeted MS2) as a separate batch.

(B) Data Analysis tab: use Peaks mode for file analysis by selecting “Analyze Selected Peaks” in “Spectral Averaging” section. Also, check “Average Fragmentation Scans.” Based on peak width in total ion chromatogram from targeted injections, set Time to Average window width to 0.10 min (less than peak full width at half maximum). Set Time to Slide Averager to 0.05 min. For Fragment Detection, increase Minimum Signal to Noise for Isotope Fitting: Terminal Fragments to 10. Compare results from setting Minimum Isotope Fit Score to 0.65 or 0.68. Finally, check “Use Tolerance *m/z* for Target Creation” and, under Proteoform Handling, check “Include N-Terminal Pyroglutamate.”

(C) Protein Identification tab: Use Annotator button to define proteoforms to be used as basis for matching observed fragment ions.

(D) Peak Picking tab: Select all files. Select the proteoform corresponding to the Lc; right click and drag across the peak set near 8 min; click “Add Peak.” Repeat for the Fd subunit, selecting the peak set near 12 min and the proteoform corresponding to the Fd with N-terminal pyroglutamate.

(E) Characterization tab: In “Observation” section, Minimum Observations set to 1 here, but this may be increased to reduce false positives. The number of observations should also be based on the number of sliding windows across a peak.

(F) Results from processed files: Sequence coverage reported for each fragmentation type recorded and compared to manual results.

(G) Characterization settings: Minimum Observations may also be adjusted after files are processed, as was done for this analysis.

**A.**


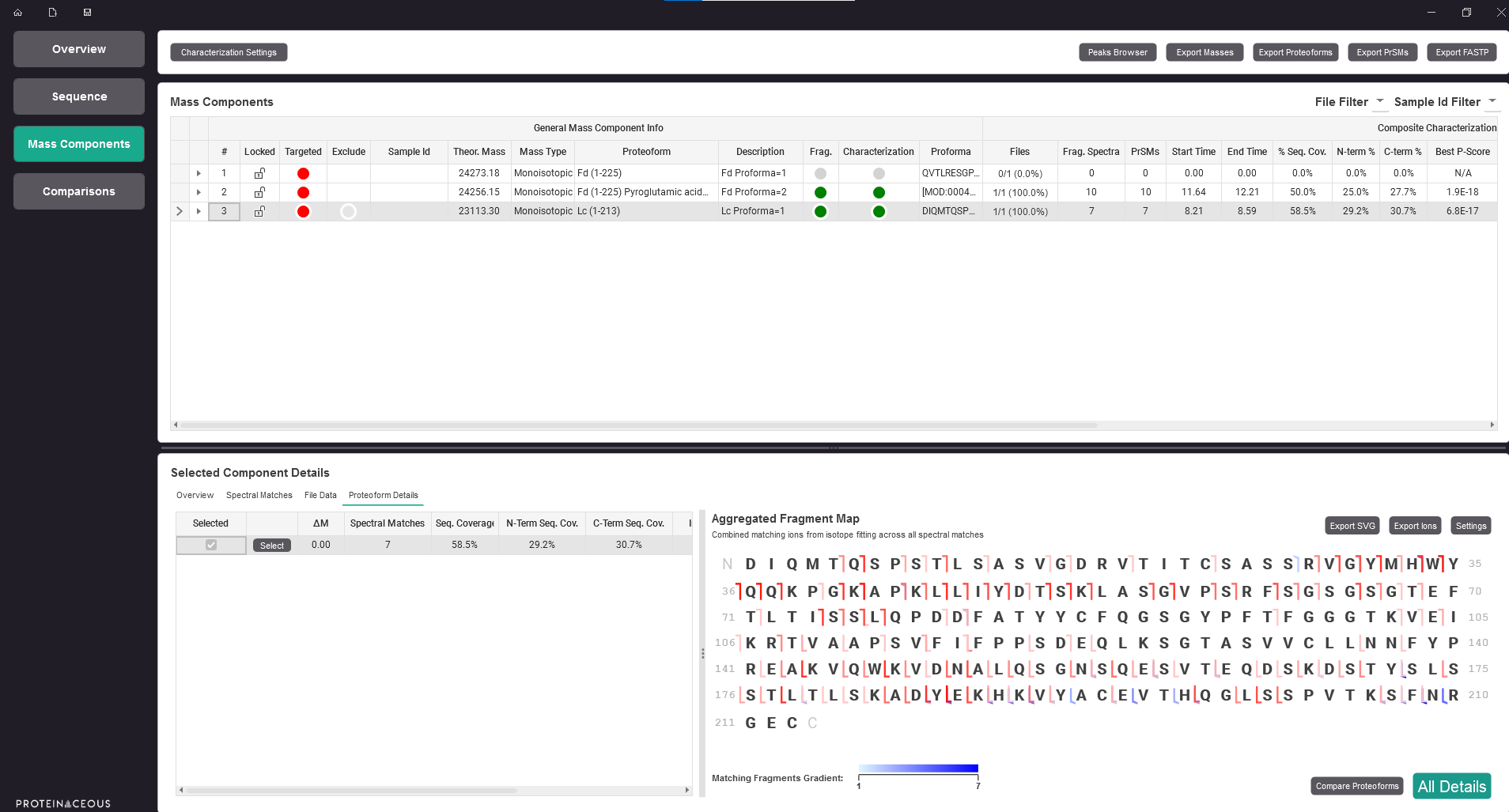


**B.**


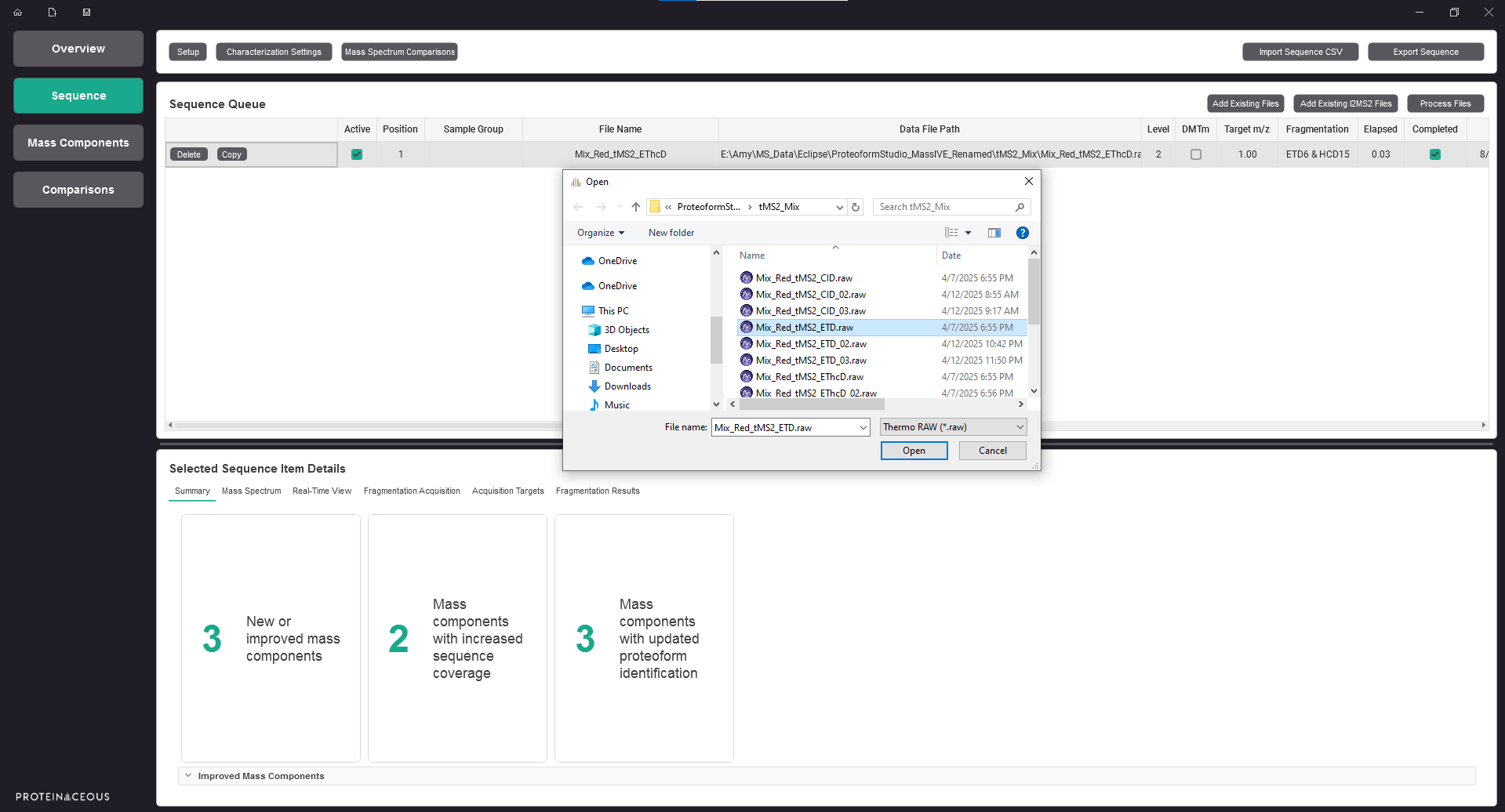


**C.**


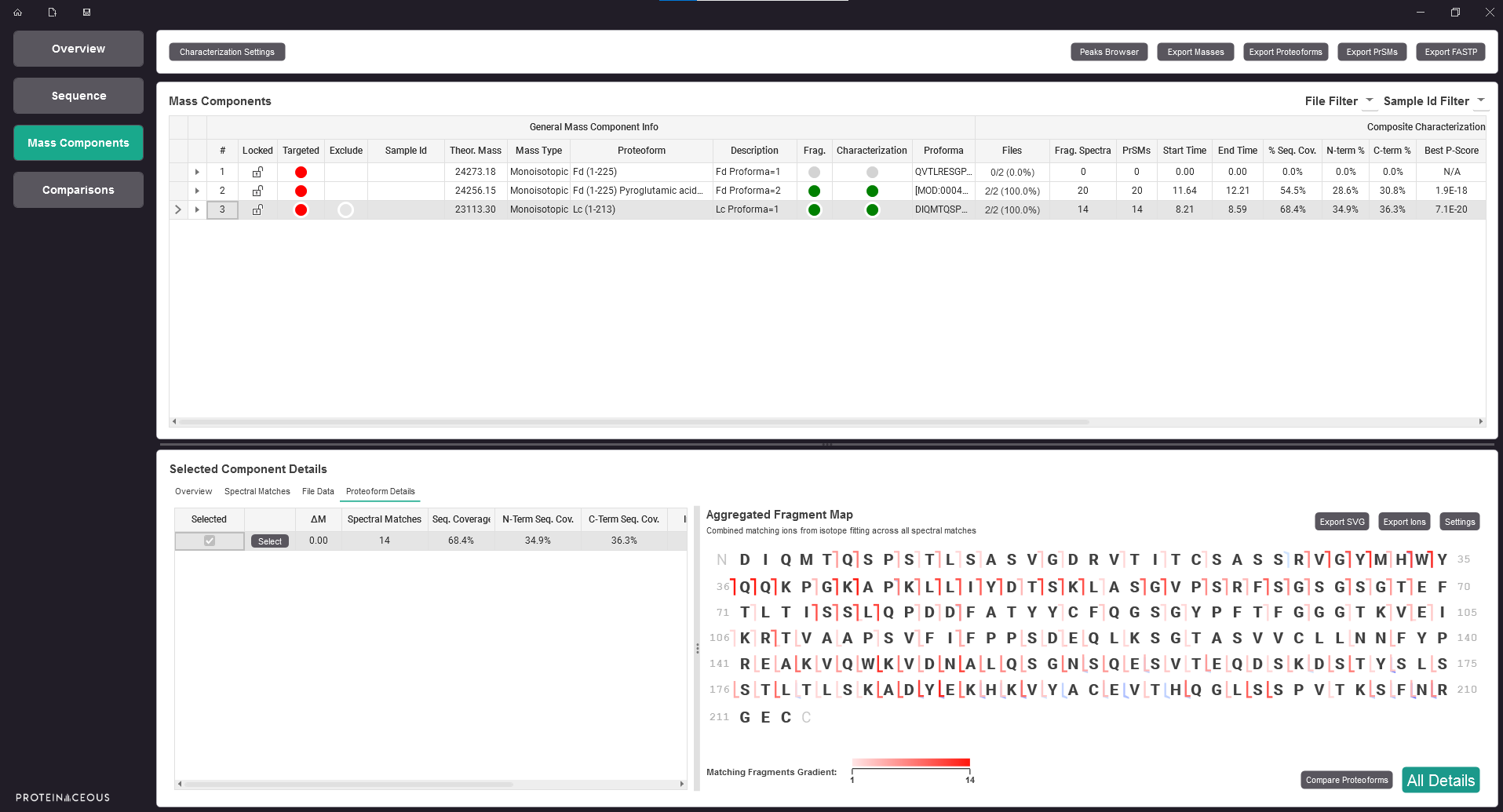


# Figure S7. Proteoform Studio Workflow Used for Cumulative Sequence Coverage Analysis Based on Aggregation of Multiple Files/Fragmentation Techniques.

Initially, only a single file was processed (in this case, MS2 EThcD fragmentation file for NIST subunits in mixture), following a similar approach as shown in Figure S3.

(A) After initial processing of file, proteform coverage recorded.

(B) Second file added; both files reprocessed (same settings).

(C) Proteoform coverage from aggregation of both files recorded. Process is then iterated using the remaining 3 files in the dataset.


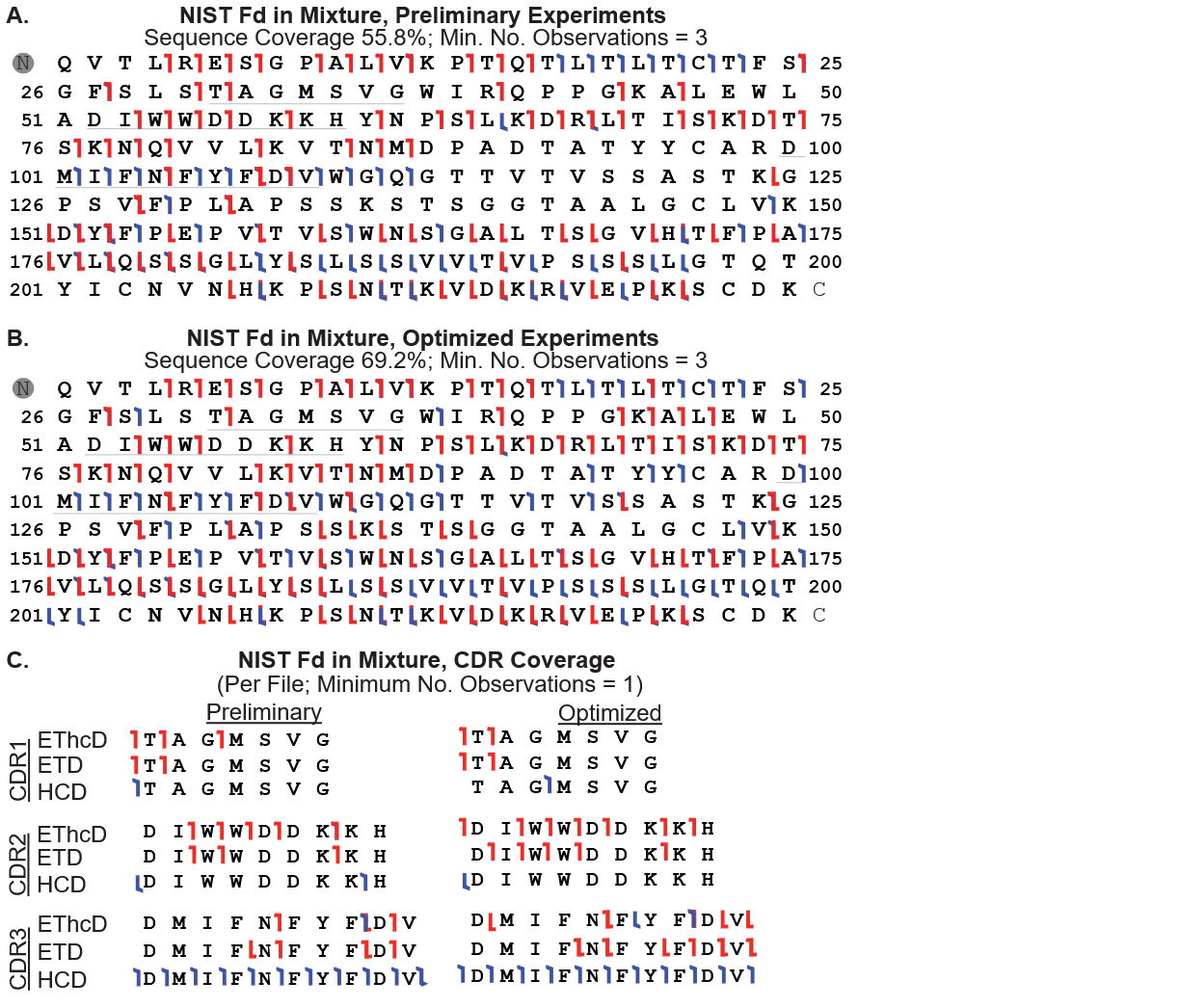


# Figure S8. Aggregate Fragment Maps, Preliminary and Optimized Experiments, NIST Fd Subunit.

Fragment support for NIST Fd subunit spiked into a simple antibody mixture was evaluated for EThcD, ETD, and HCD fragmentation using two different sets of parameters. Preliminary experiments used 6 ms ETD reaction time for ETD and EThcD and 30% NCE for HCD for analysis of NIST Fd (A.). Optimized experiments used estimated higher on-column load as well as 5 ms ETD reaction time and higher charge state targets for ETD and EThcD, as well as 35% NCE for HCD for analysis of NIST Fd (B.). Complementarity-determining regions (CDRs) are underlined.^4^ The increased coverage observed from combining results from different fragmentation techniques is apparent from considering fragment support for the CDR regions from each technique evaluated separately (C.).


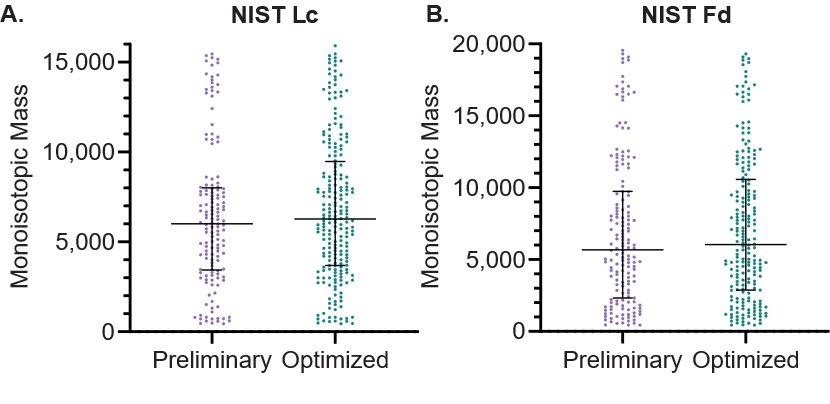


# Figure S9. Scatterplots of Assigned Fragment Ion Monoisotopic Masses for the Preliminary versus Optimized Datasets.

Results reflect composite of EThcD, ETD, and HCD fragmentation, with minimum number of observations set to 3. Lines show median and interquartile range.


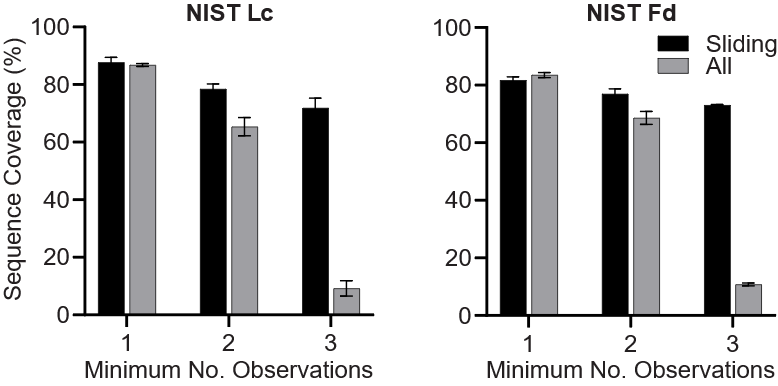


# Figure S10. Targeted Proteoform Analysis: Average Composite Sequence Coverage for Sliding Window versus “All” Window Mode.

Sequence coverage observed from Proteoform Studio targeted analysis workflow using a sliding window width of 0.1 min and time to slide 0.05 min (“Sliding”) or window mode all (“All”). Analysis was performed for each of 3 replicate file sets by first analyzing an EThcD file individually (including defining retention time bounds of the Lc and Fd peaks), followed by determining composite coverage from combining results with ETD, then combining HCD, CID, and UVPD. Bars show average sequence coverage for aggregation of 5 fragmentation techniques for the 3 replicate file sets (error bars indicate standard deviation) for minimum number of observations of 1, 2, and 3 for each subunit.


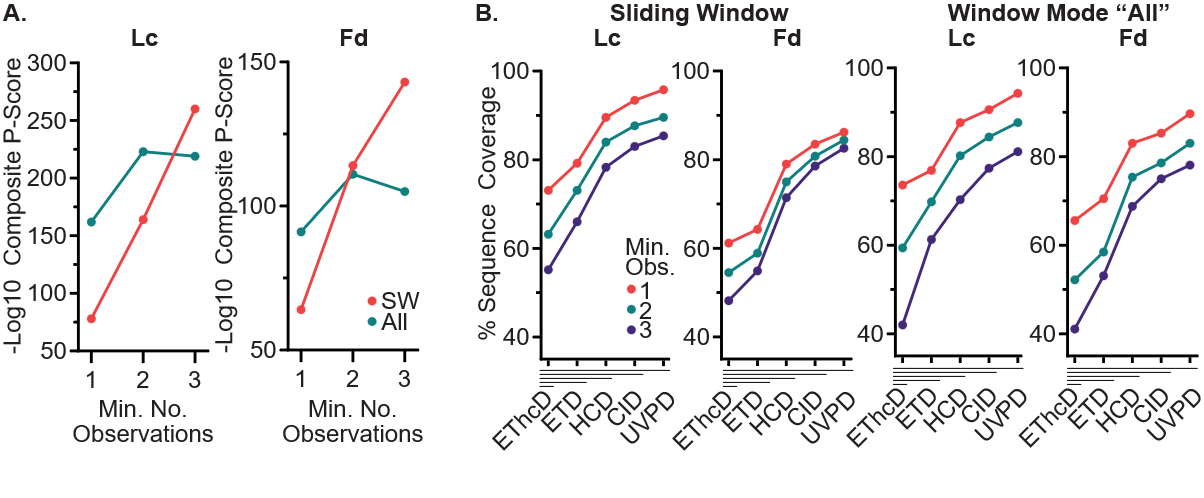


# Figure S11. Composite P-Score and Sequence Coverage Comparison, Sliding Window versus “All” Window Mode, NIST in Mixture.

(A.) -Log_10_(composite P-score) calculated by Proteoform Studio for the NIST Lc and Fd subunits based on considering all 5 fragmentation techniques for all 3 replicates (15 files total). Analysis was performed using a sliding window width of 0.1 min and time to slide 0.05 min (sliding window, “SW”) or window mode all (“All”) requiring 1, 2, and 3 minimum fragment observations. Here, rather than defining retention time bounds for peaks corresponding to the NIST Lc and Fd proteoforms using the EThcD files only, all 15 files were used to define these bounds during the peak picking step of the workflow.

(B.) Sequence coverage observed from Proteoform Studio targeted analysis workflow by first aggregating all 3 EThcD replicates, using a sliding window width of 0.1 min and time to slide 0.05 min (“Sliding Window”) or window mode “all”, followed by the composite coverage from combining results with all 3 ETD replicates, then combining all HCD, CID, and UVPD replicates. Coverage for 1, 2, and 3 minimum fragment observations is plotted for each subunit.

# Internal Fragment Analysis

EThcD and HCD data for NIST Lc and Fd subunits in a simple antibody mixture from the “optimized” dataset were used to evaluate inclusion of internal fragments. Analysis was first performed using manual validation of fragment assignment (TDValidator in ProSight Native, v1.0.25108). Next, automated processing using Proteoform Studio (v1.0.25206) was performed, varying error tolerance and S/N and score thresholds for internal fragment ion assignment. For purposes of this analysis, the focus was on assessing the extent to which accepted internal fragment ions contributed to increasing sequence coverage for two different fragmentation techniques, as well as on whether Proteoform Studio settings could be tuned such that changes in sequence coverage based on inclusion of internal fragments reflected that observed from the manual validation.

Specifically, for the manual validation, settings were the same as those used previously for validation of terminal ions (fragment ppm tolerance 10 ppm; S/N cutoff 10.0, minimum score 0.50 for terminal ions), except “Include Internal Fragments” was checked; a minimum score of 0.70 (default setting) for internal ions was used. Terminal ions removed during the previous manual validation were removed from consideration. The total number of internal ions assigned for each subunit for each mode is summarized in **Table S9.** Internal ions were rejected if any of the following applied:

1. internal ion isobaric with another terminal or internal ion assigned, regardless of whether the other ion was accepted after manual validation
2. internal ion assigned to same set of peaks, or an overlapping set of peaks, as another terminal or internal ion, regardless of whether the other ion was accepted after manual validation
3. internal ion mass error > 3 ppm from average mass error of accepted terminal ions
4. internal ion length < 5 amino acids
5. internal ion signal not distinct from noise
6. qualitative judgement that observed signal inconsistent with theoretical distribution, or that observed signal insufficiently intense to distinguish from surrounding signal

Mass error and length used for criteria iii and iv are based on Wei et al.^5^ TDValidator allows for exporting a list of fragment assignments by ion name and charge; in general, approximately half of such internal fragment assignments were eliminated based on mass error alone (criterion iii above). For the remaining internal fragment assignments, the presence of multiple assignments for the same set of peaks (criteria i and ii) and insufficient S/N (criterion v) accounted for the majority of manual rejections. Multiple assignments for the same peaks was more prevalent for EThcD than HCD, as expected based on the greater number of fragment types considered. As a result, only a minimal number of internal fragments were accepted for EThcD data in the case of both the Lc and the Fd subunit.

# Table S9. Internal Ions Assigned and Accepted, Manual Validation

| Mode | Subunit | Total No. Internal^a^ | No. Internal Accepted^a^ |
| --- | --- | --- | --- |
| EThcD | Lc | 434 | 2 |
| EThcD | Fd | 384 | 3 |
| HCD | Lc | 598 | 49 |
| HCD | Fd | 683 | 62 |

^a^Totals by ion name (multiple charge states for the same ion, where present, collapsed into one count).

The limited number of internal fragment ions confidently assigned for the EThcD data considered here is consistent with a recent study evaluating internal fragmentation for electron capture dissociation (ECD) fragmentation.^6^ However, as shown by **Figure S16**, an internal fragment assignment that was accepted for the NIST Lc (C-terminal to A111) corresponds to a sequence region with sparse coverage based on terminal fragment assignment alone.


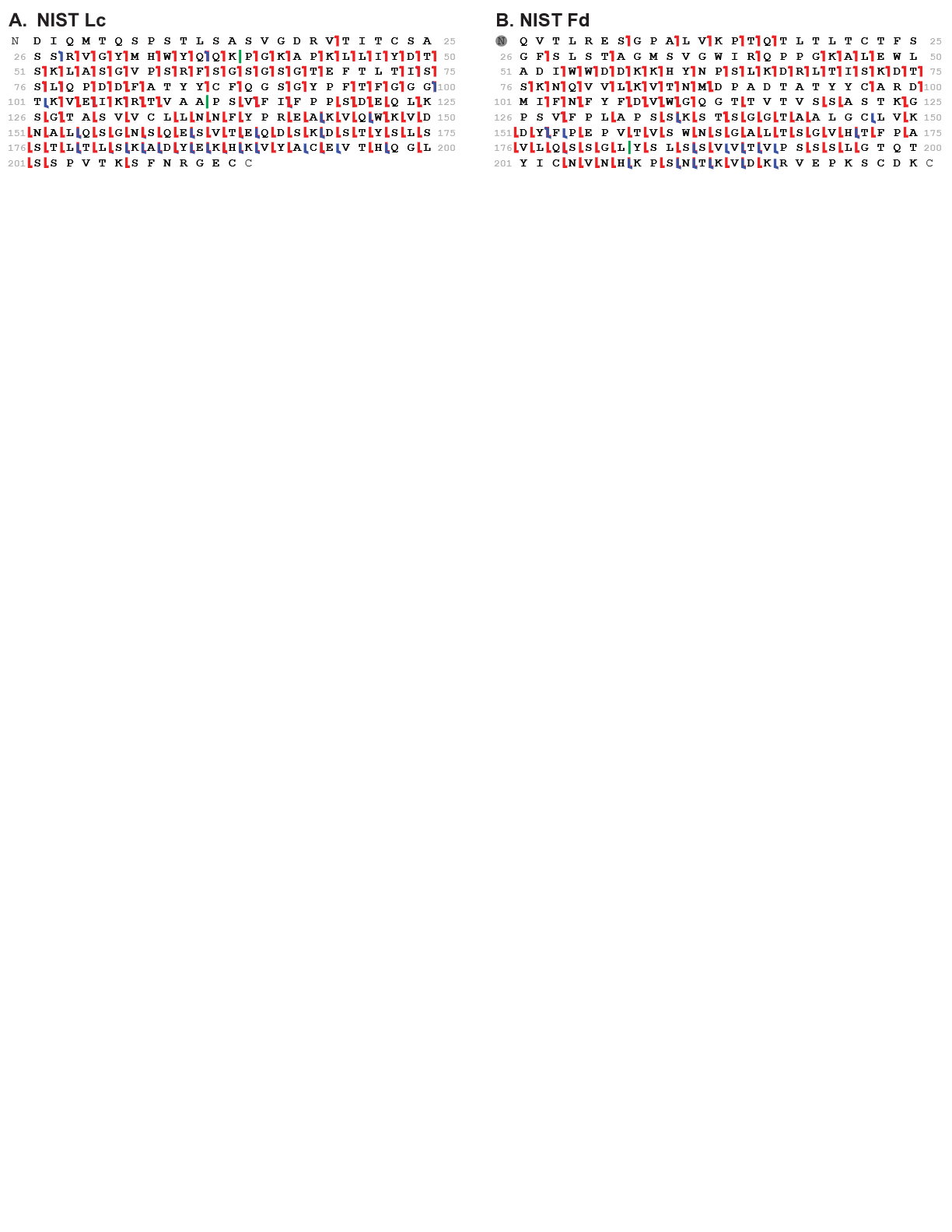


# Figure S12. Additional Cleavage Locations Identified Based on Manually Validated EThcD Internal Fragments.

Fragment maps, NIST Lc (A) and Fd (B) subunits, EThcD fragmentation using “optimized” settings. Additional cleavage locations identified based on internal fragment ion assignments are indicated in green, with terminal fragments shown in blue (b/y) and red (c/z).

A greater number of internal fragment assignments were accepted for the HCD data, with the benefit in terms of improved characterization most apparent for the terminal regions of each subunit (**Figure S17**).


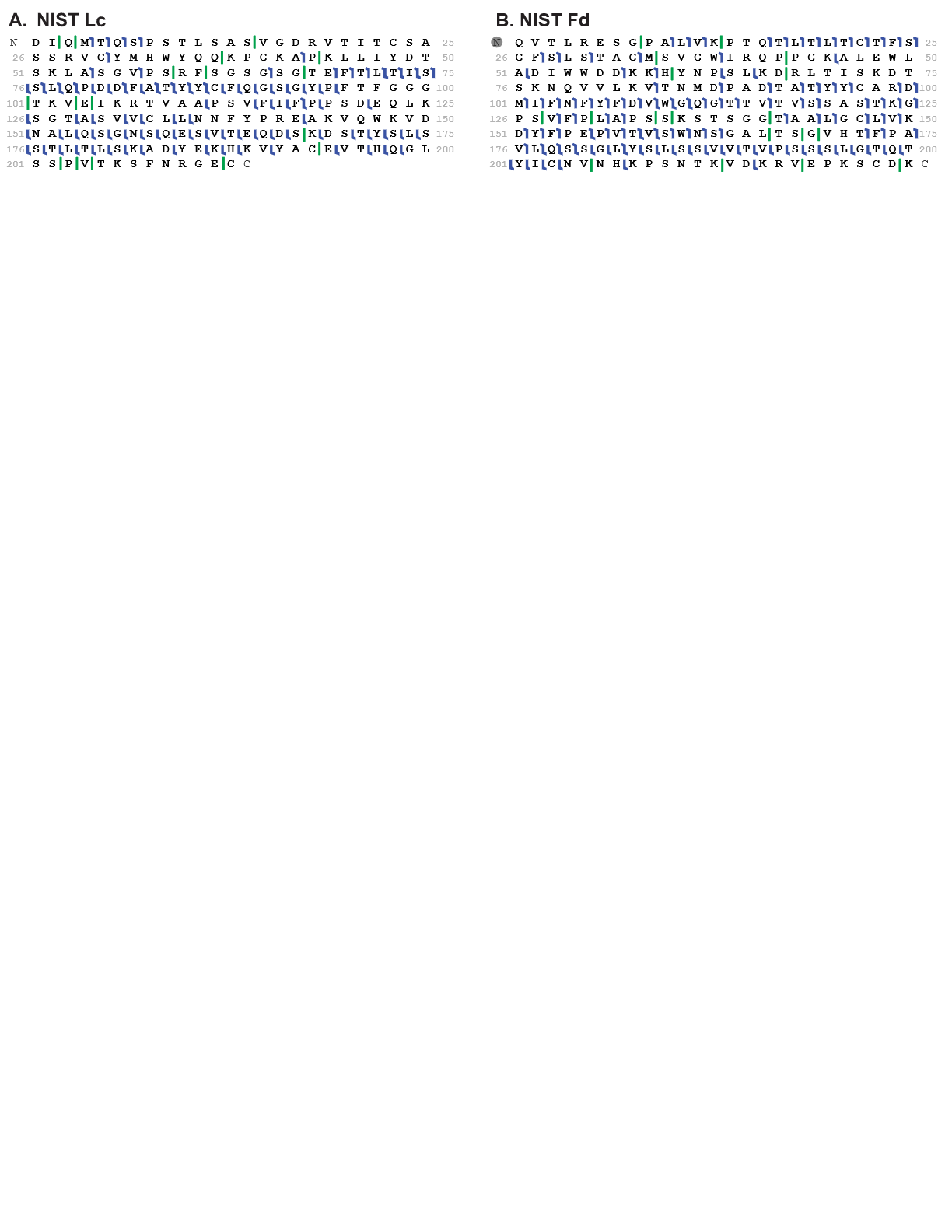


# Figure S13. Additional Cleavage Locations Identified Based on Manually Validated HCD Internal Fragments.

Fragment maps, NIST Lc (A) and Fd (B) subunits, HCD fragmentation using “optimized” settings. Additional cleavage locations identified based on internal fragment ion assignments are indicated in green, with terminal fragments shown in blue (b/y).

Results support the potential value of incorporating internal fragment information for improving antibody subunit characterization.^3, 5^ However, especially in the case of EThcD data, the manual analysis indicates that additional scrutiny of internal assignments may be warranted. Proteoform Studio allows for consideration of internal fragment ions, and, unlike TDValidator, permits different mass error tolerances and S/N thresholds to be defined for terminal versus internal ions, supporting the increased mass stringency that has been applied for internal ion analysis.^3, 5, 7^

As a first step, internal fragment parameters were adjusted to evaluate if automated analysis results approximating those observed for the manual analysis were observed. The same files subjected to manual validation were analyzed via Proteoform Studio, first by using the default internal fragment ion settings, with mass tolerance of 10 ppm, post-calibration mass tolerance of 3 ppm, minimum S/N 10, and minimum score 0.70. (Other settings were kept consistent with those used for previous automated processing in Proteoform Studio that included terminal ions only; specifically, the default settings for fragmentation spectra averaging were used [window mode: time; time to average, 0.50 min; time to slide average: 0.25 min], with terminal fragment detection performed using 10 ppm *m/z* tolerance, minimum S/N 10, and minimum fit score 0.68. Minimum number of observations was set to 1.) Next, more stringent mass tolerances were used (3 ppm, mass tolerance and post-calibration mass tolerance; 3 ppm mass tolerance and 1 ppm post-calibration mass tolerance), with values informed by Wei et al. and Schmitt et al.^5, 7^ The analysis was also performed with more stringent S/N (minimum 30) and score (minimum 0.72). These values were based on consideration of all internal fragment assignments accepted for the four manual validations performed; 88% of accepted fragments exhibited S/N at least 30, and 92% exhibited score of at least 0.72 (different charge states of the same fragment were considered separately for this calculation). As shown by **Table S10**, in general, even when applying stringent settings in Proteoform Studio, the number of internal fragment ions assigned is greater than the number accepted in the manual analysis.

# Table S10. Internal Ions Assigned, Proteoform Studio.

|  |  | Total No. Internal Ions | | | | | No. Internal Ions after Manual Elimination of Ambiguous Assignments^a^ | |
| --- | --- | --- | --- | --- | --- | --- | --- | --- |
| Mode | Subunit | Error: 10 ppm, 3 ppm post-calibration; Score: 0.70; S/N: 10.0 | Error: 3 ppm, 3 ppm post-calibration; Score: 0.70; S/N: 10.0 | Error: 3 ppm, 1 ppm post-calibration; Score: 0.70; S/N: 10.0 | Error: 3 ppm, 1 ppm post-calibration; Score: 0.70; S/N: 30.0 | Error: 3 ppm, 1 ppm post-calibration; Score: 0.72; S/N: 30.0 | Error: 10 ppm, 3 ppm post-calibration; Score: 0.70; S/N: 10.0 | Error: 3 ppm, 1 ppm post-calibration; Score: 0.72; S/N: 30.0 |
| EThcD | Lc | 381 | 97 | 29 | 18 | 15 | 238 | 4 |
| EThcD | Fd | 544 | 162 | 50 | 41 | 34 | 228 | 12 |
| HCD | Lc | 725 | 307 | 203 | 168 | 144 | 357 | 78 |
| HCD | Fd | 812 | 269 | 126 | 107 | 87 | 389 | 36 |

^a^Assignment considered ambiguous if monoisotopic mass was within 10 ppm of another fragment ion.

This is further reflected in the substantially higher sequence coverage observed when incorporating internal fragment ions identified based on automated analysis in Proteoform Studio as compared to the manual analysis (**Tables S11** and **S12**). Proteoform Studio reports all assigned internal fragment ions based on the defined error tolerance, S/N, and score criteria. Sequence coverage and new cleavage sites provided by internal ion assignments were recalculated after manually removing internal fragment ions with monoisotopic mass within 10 ppm of another fragment ion, for both the most stringent and least stringent internal fragment ion settings used here. In this case, the overall sequence coverage observed for the most stringent Proteoform Studio settings considered is more consistent with that observed for the manual analysis (**Table S13**). However, limited overlap was observed between internal ions assigned in the manual versus automated analyses. Specifically, for the combined 116 ions accepted during the manual analysis (here, collapsing multiple charge states for the same ion into one ion assignment), only 25 were included in the Proteoform Studio results, with all but 4 corresponding to HCD fragments for the Lc subunit.

In general, the lack of overlap may be rationalized by the different stringency and reference point for mass error calculations in the manual versus automated analysis. In the manual analysis, as noted, the accepted mass error range was + 3 ppm of the average mass error of accepted terminal fragments (rather than -3 to 3 ppm as used for the automated analysis). Of the 91 internal ions accepted during manual analyses that were not included in the Proteoform Studio results, 46 exhibit a mass error less than -3 ppm, despite being within 3 ppm of the average internal fragment error; an additional 9 ions are removed from those assigned by Proteoform Studio when reducing the mass tolerance from 10 to 3 ppm, and an additional 7 are removed when reducing the post-calibration error tolerance from 3 to 1 ppm. On the other hand, 110 of the 116 internal ions assigned during manual analysis were also assigned by Proteoform Studio when using the default internal fragment ion settings combined with manual removal of internal ions with monoisotopic mass within 10 ppm of another assigned ion.

# Table S11. Internal Fragment Ion Contribution to Sequence Coverage, Manual Validation.

| Mode | Subunit | % Coverage, Terminal Only | New Backbone Cleavage Sites from Internal | % Coverage, Terminal + Internal^a^ |
| --- | --- | --- | --- | --- |
| EThcD | Lc | 67.0 | 2 | 67.9 |
| EThcD | Fd | 55.8 | 1 | 56.2 |
| HCD | Lc | 42.0 | 17 | 50.0 |
| HCD | Fd | 48.7 | 18 | 56.7 |

^a^Error: initially set to 10 ppm, and rejected internal fragments if more than 3 ppm from average error of accepted terminal fragments; score threshold: 0.70; minimum S/N: 10. Coverage from terminal fragment ions only reflects results from previous manual validation excluding internal fragment ions.

# Table S12. Internal Fragment Ion Contribution to Sequence Coverage, Automated Analysis in Proteoform Studio.

| Mode | Subunit | % Coverage, Terminal Only | Error: 10 ppm,  3 ppm post-calibration;  Score: 0.70;  S/N: 10.0 | | Error: 3 ppm,  3 ppm post-calibration;  Score: 0.70;  S/N: 10.0 | | Error: 3 ppm,  1 ppm post-calibration;  Score: 0.70;  S/N: 10.0 | | Error: 3 ppm,  1 ppm post-calibration;  Score: 0.70;  S/N: 30.0 | | Error: 3 ppm,  1 ppm post-calibration;  Score: 0.72;  S/N: 30.0 | |
| --- | --- | --- | --- | --- | --- | --- | --- | --- | --- | --- | --- | --- |
|  |  |  | **Sites**^a^ | **%** | **Sites** | **%** | **Sites** | **%** | **Sites** | **%** | **Sites** | **%** |
| EThcD | Lc | 69.8 | 62 | 99.1 | 37 | 87.3 | 14 | 76.4 | 13 | 75.9 | 11 | 75.0 |
| EThcD | Fd | 58.5 | 92 | 99.6 | 63 | 86.6 | 31 | 72.3 | 24 | 69.2 | 18 | 66.5 |
| HCD | Lc | 40.1 | 125 | 99.1 | 97 | 85.8 | 74 | 75.0 | 68 | 72.2 | 58 | 67.5 |
| HCD | Fd | 48.7 | 109 | 97.3 | 93 | 90.2 | 59 | 75.0 | 52 | 71.9 | 46 | 69.2 |

^a^Sites: new backbone cleavage sites from internal; %: % sequence coverage, terminal plus internal ions.

# Table S13. Internal Fragment Ion Contribution to Sequence Coverage, Automated Analysis in Proteoform Studio and Manual Curation Based on Mass Similarity.

| Mode | Subunit | % Coverage, Terminal Only | Error Tol.: 10 ppm,  3 ppm post-calibration;  Score: 0.70;  S/N: 10.0 | | Error Tol.: 3 ppm,  1 ppm post-calibration;  Score: 0.72;  S/N: 30.0 | |
| --- | --- | --- | --- | --- | --- | --- |
|  |  |  | **New Fragmentation Sites from Internal** | **% Coverage, Terminal + Internal** | **New Fragmentation Sites from Internal** | **% Coverage, Terminal + Internal** |
| EThcD | Lc | 69.8 | 56 | 96.2 | 2 | 70.8 |
| EThcD | Fd | 58.5 | 74 | 91.5 | 5 | 60.7 |
| HCD | Lc | 40.1 | 111 | 92.5 | 37 | 57.5 |
| HCD | Fd | 48.7 | 97 | 92.0 | 26 | 60.3 |

In sum, the present analysis supports the value of considering internal fragment ions for antibody subunit characterization, and this analysis is supported by Proteoform Studio features that allow for more stringent mass tolerance, S/N, and score settings for internal fragments. However, given the potential for ambiguous assignments, especially for fragmentation techniques such as EThcD that may generate a greater number of fragment ion types, the present analysis indicates that fully automated assignment of internal fragments should be approached with caution. Additional visualization features and analysis tools incorporated in Proteoform Studio provide support for manual evaluation of internal fragment ion assignments. For example, as shown in **Figure S14**, fragment ion assignments for each proteoform spectral match may be checked visually in the TDValidator tool incorporated in Proteoform Studio (**Figure S14B**); ion assignments may then be removed from the list of matching fragment ions **Figure S14C**), and the effect on the overall sequence coverage visualized in a fragment map (**Figure S14D**). Internal fragment ion assignments may also be aggregated and visualized at the proteoform level, as shown in **Figure S15**. In addition to decreasing the mass error tolerance and increasing the minimum S/N and score, the stringency of internal fragment ion assignments may also be controlled by increasing the required minimum number of observations of an internal ion for a given dataset.

**A.**


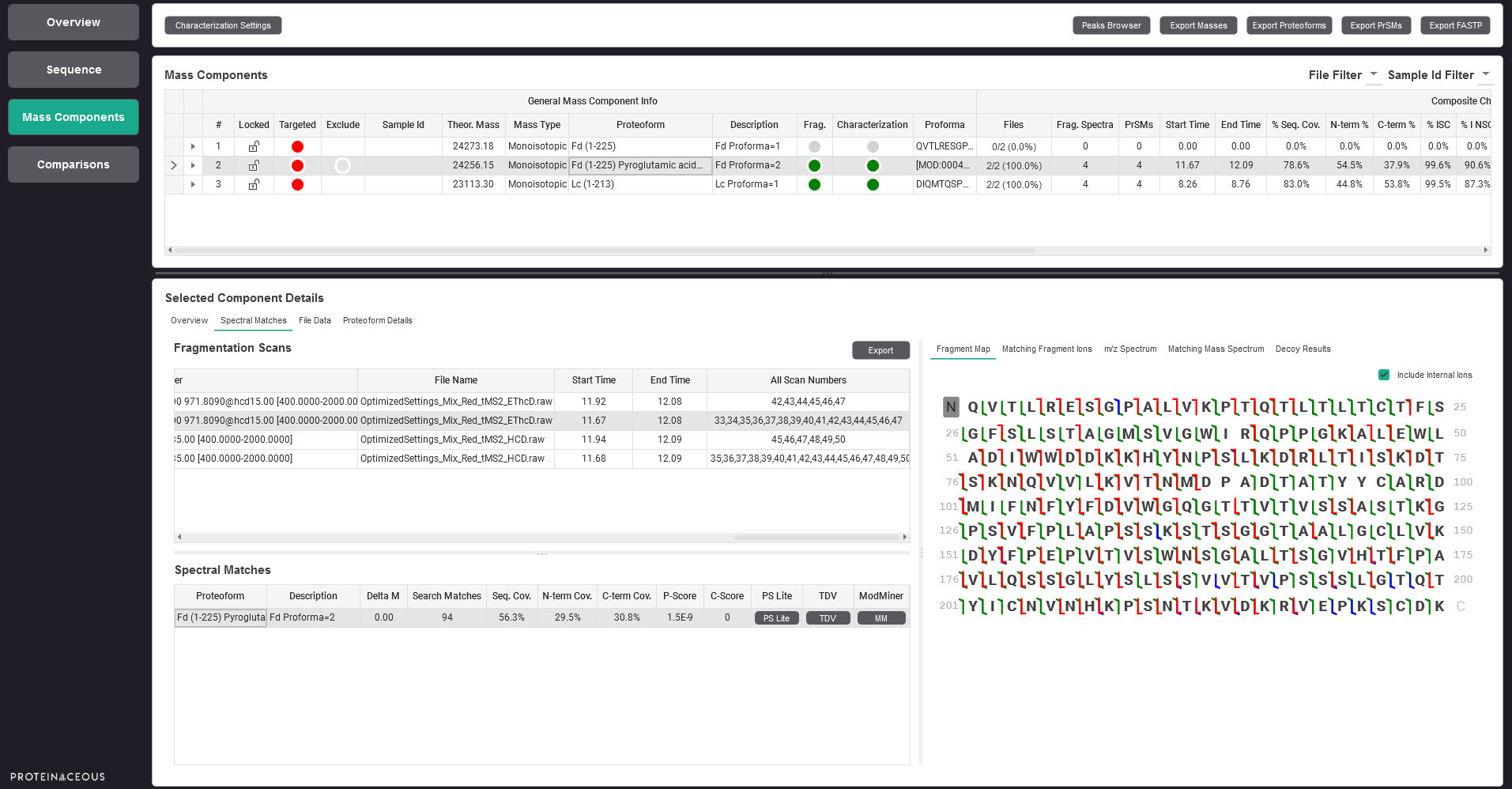


**B.**


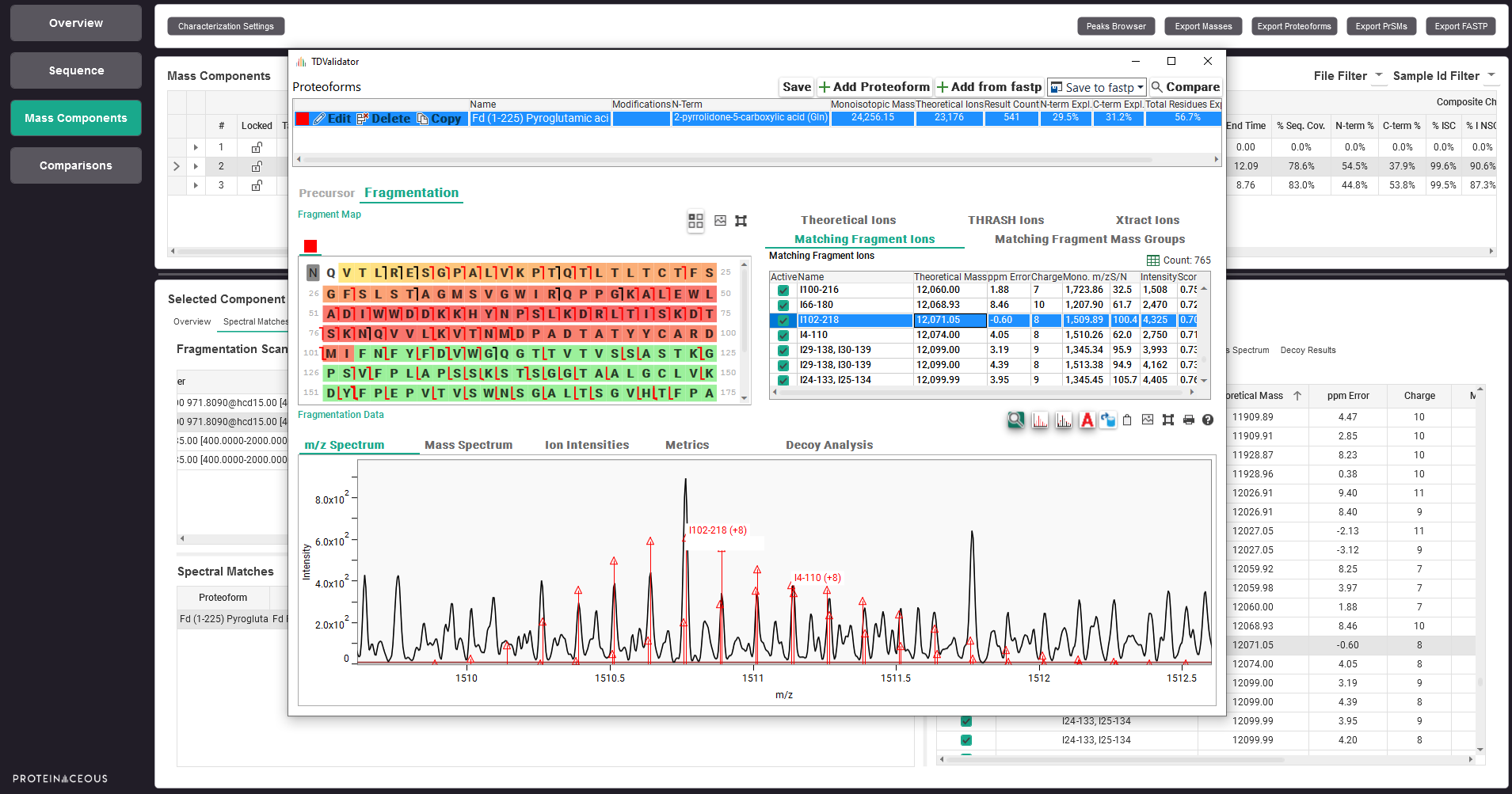


**C.**

**
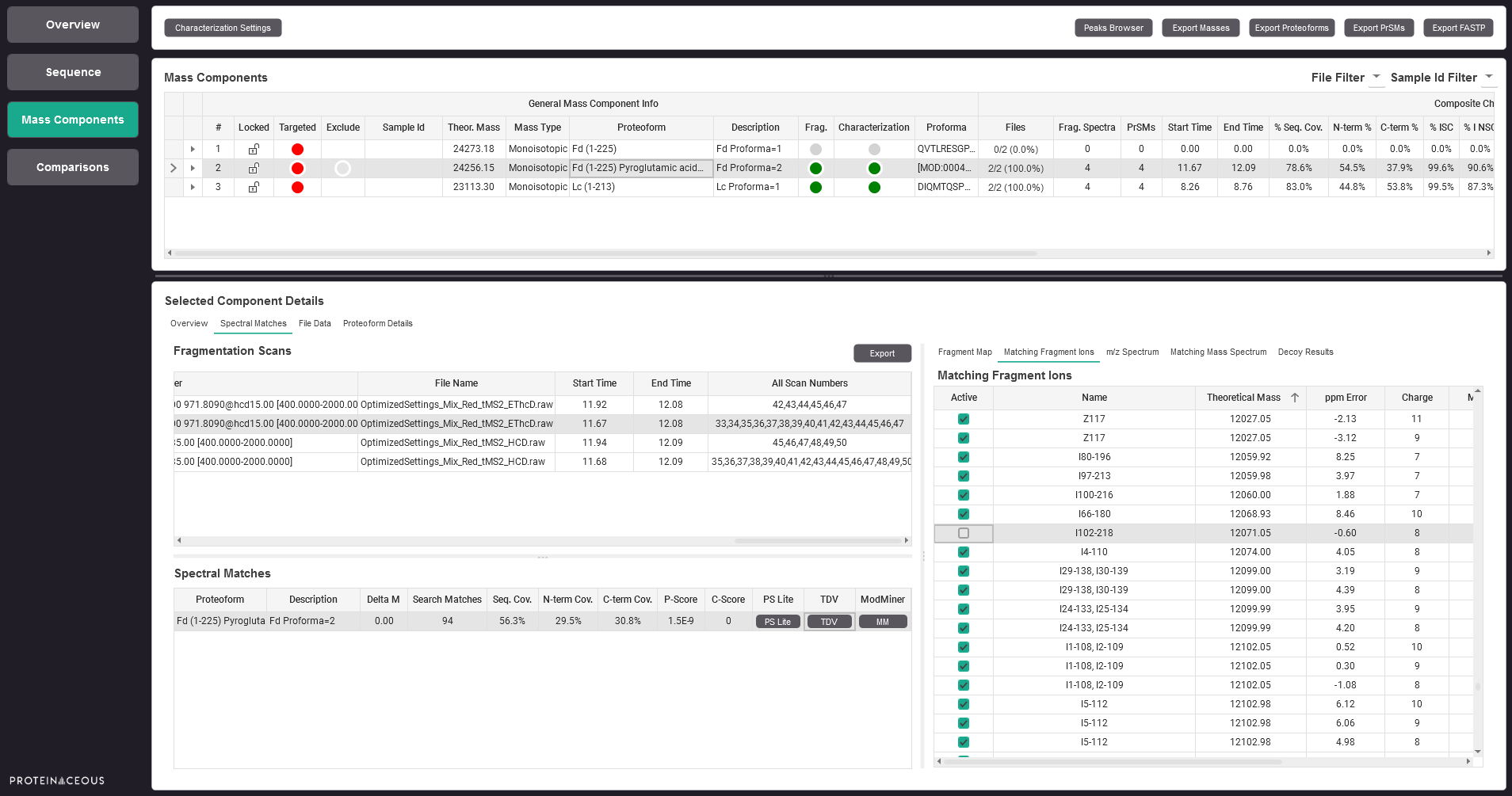
**

**D.**


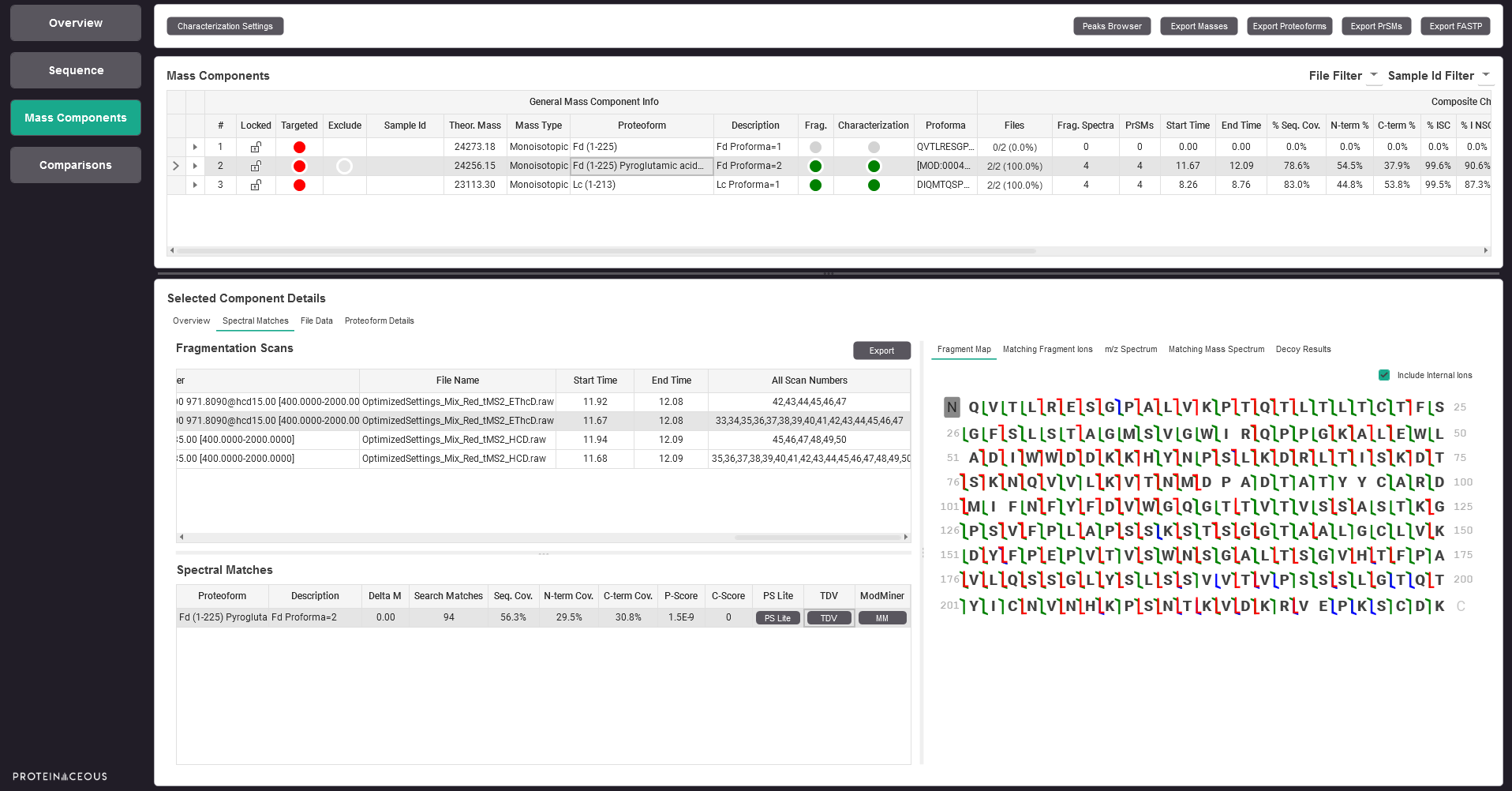


# Figure S14. Internal Fragment Ion Assignment Evaluation in Proteoform Studio.

Default internal fragment ion settings were used here (mass tolerance 10 ppm; post-calibration tolerance 3 ppm; minimum S/N 10; minimum score 0.70).

**A.** Fragment ion assignments may be evaluated for each spectral match. (Here, a spectral match refers to a set of averaged spectra within a given window for a given file, since fragmentation spectra averaging using sliding window mode was enabled for this analysis.)

**B.** Specific ion assignments may be evaluated using the built-in TDValidator tool in Proteoform Studio.

**C.** Ion assignments may be removed from the Matching Fragment ions list.

**D.** The resulting effect on overall sequence coverage may be visualized based on the updated fragment map.

**A.**


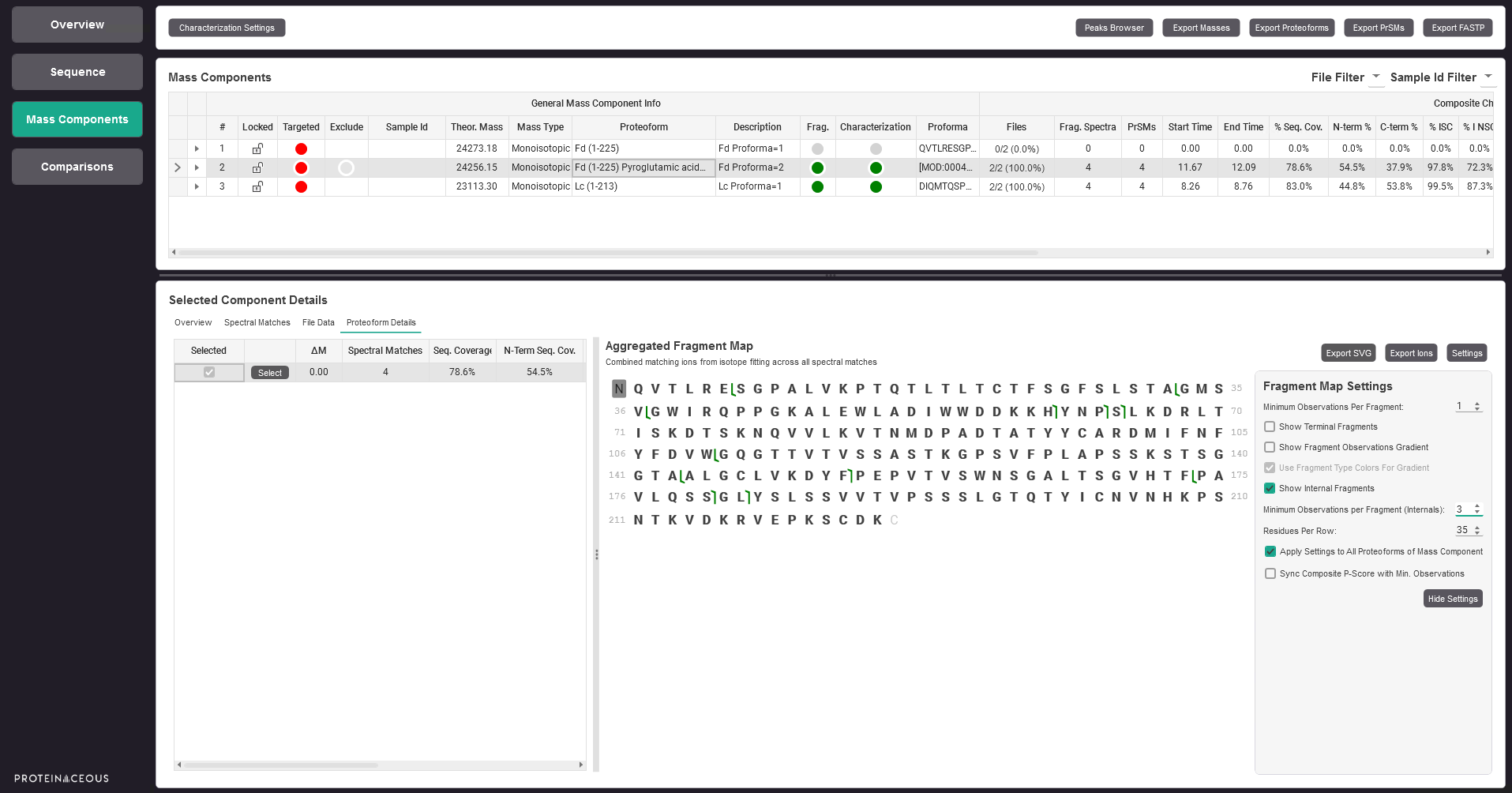


**B.**


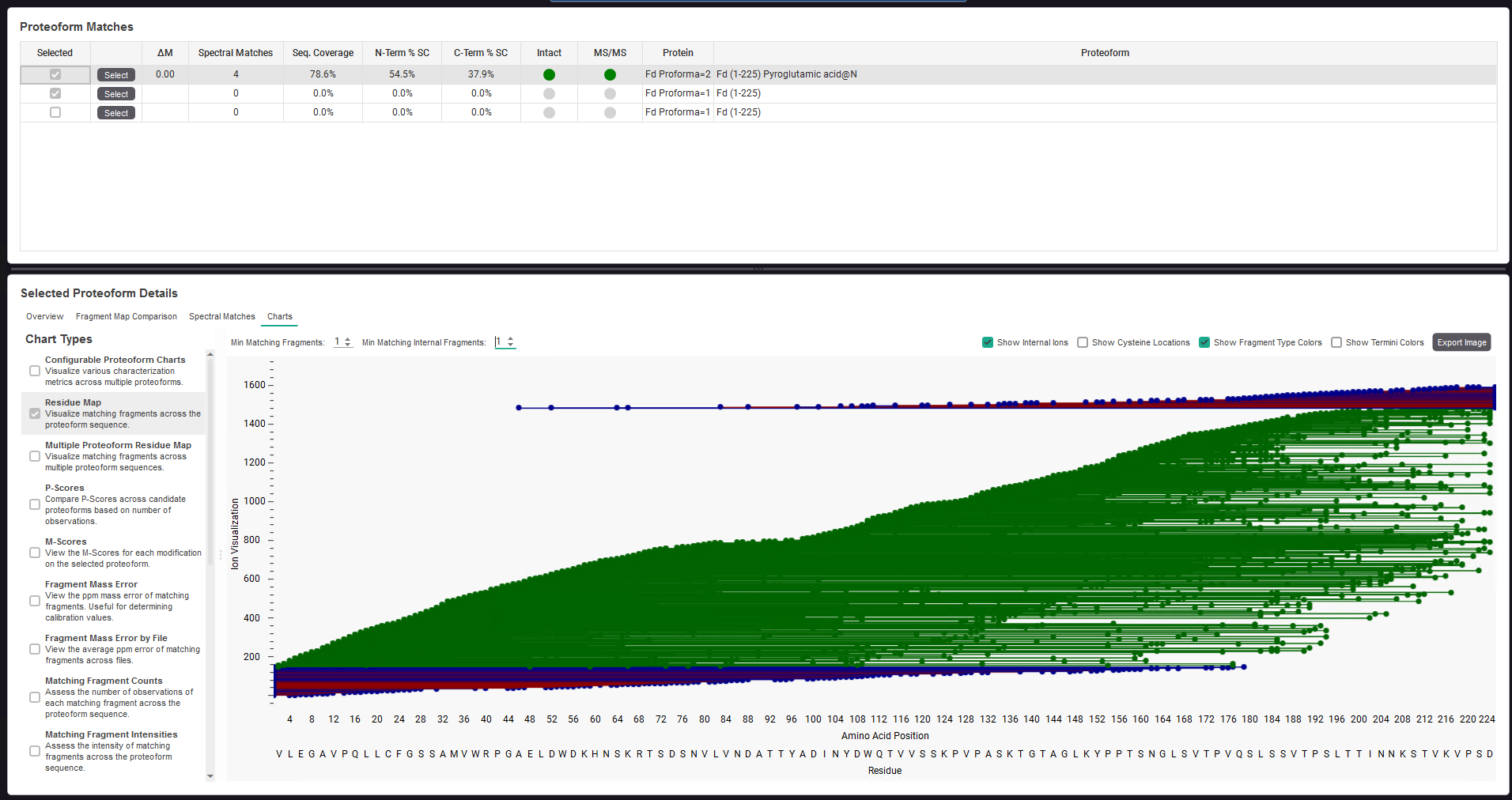


**C.**


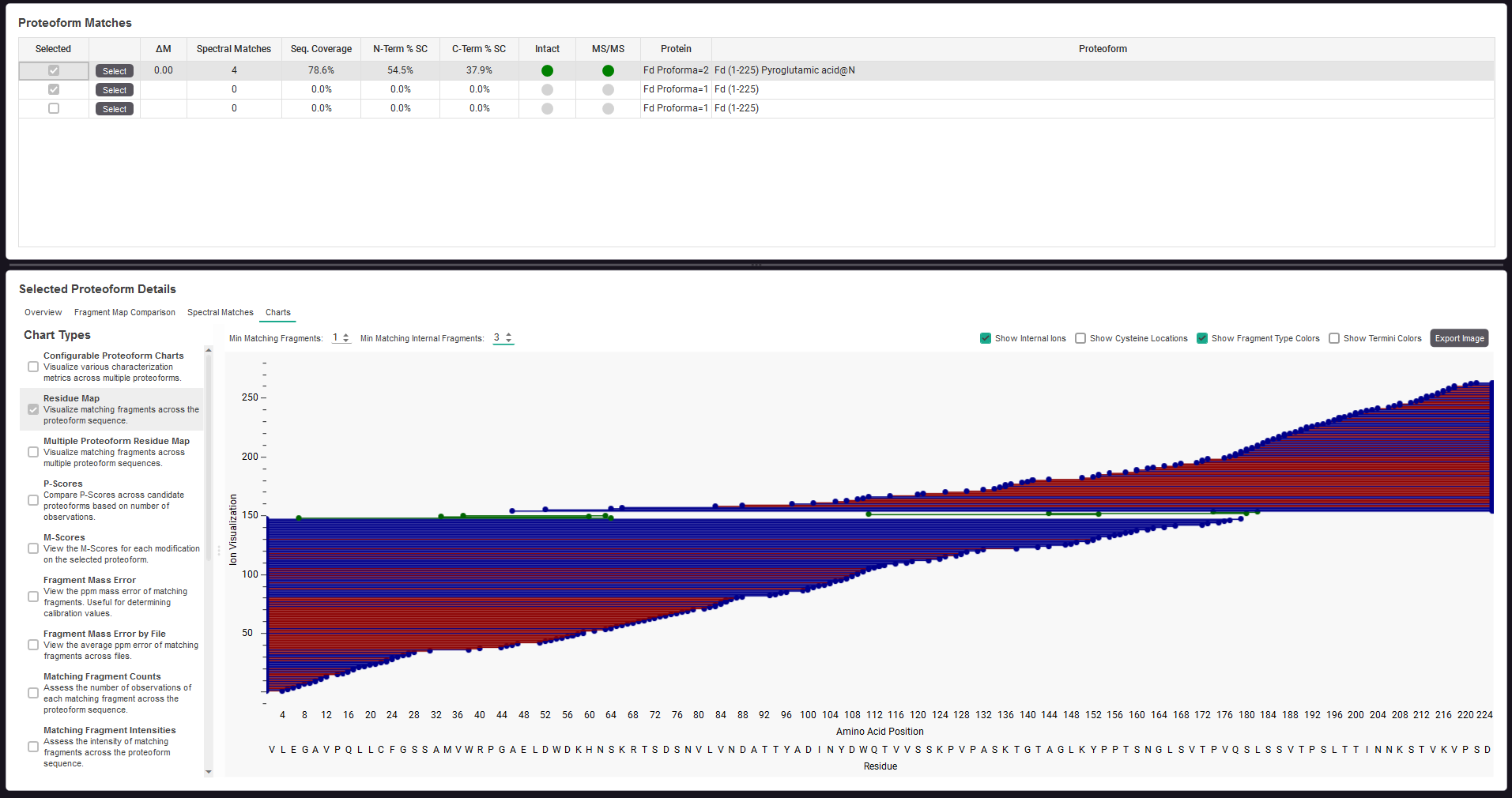


# Figure S15. Internal Fragment Ion Visualization in Proteoform Studio.

As for terminal ion assignments, internal fragment ion assignments from all files analyzed may be viewed in an aggregated fragment ion map (**A**); minimum number of observations for internal ions may be similarly controlled. Ion assignments may also be visualized using residue maps, which in this case show a substantial difference in retained internal fragment ions when minimum matching number of internal fragments is increased from 1 (**B**) to 3 (**C**) for the small dataset considered here (1 EThcD and 1 HCD file).


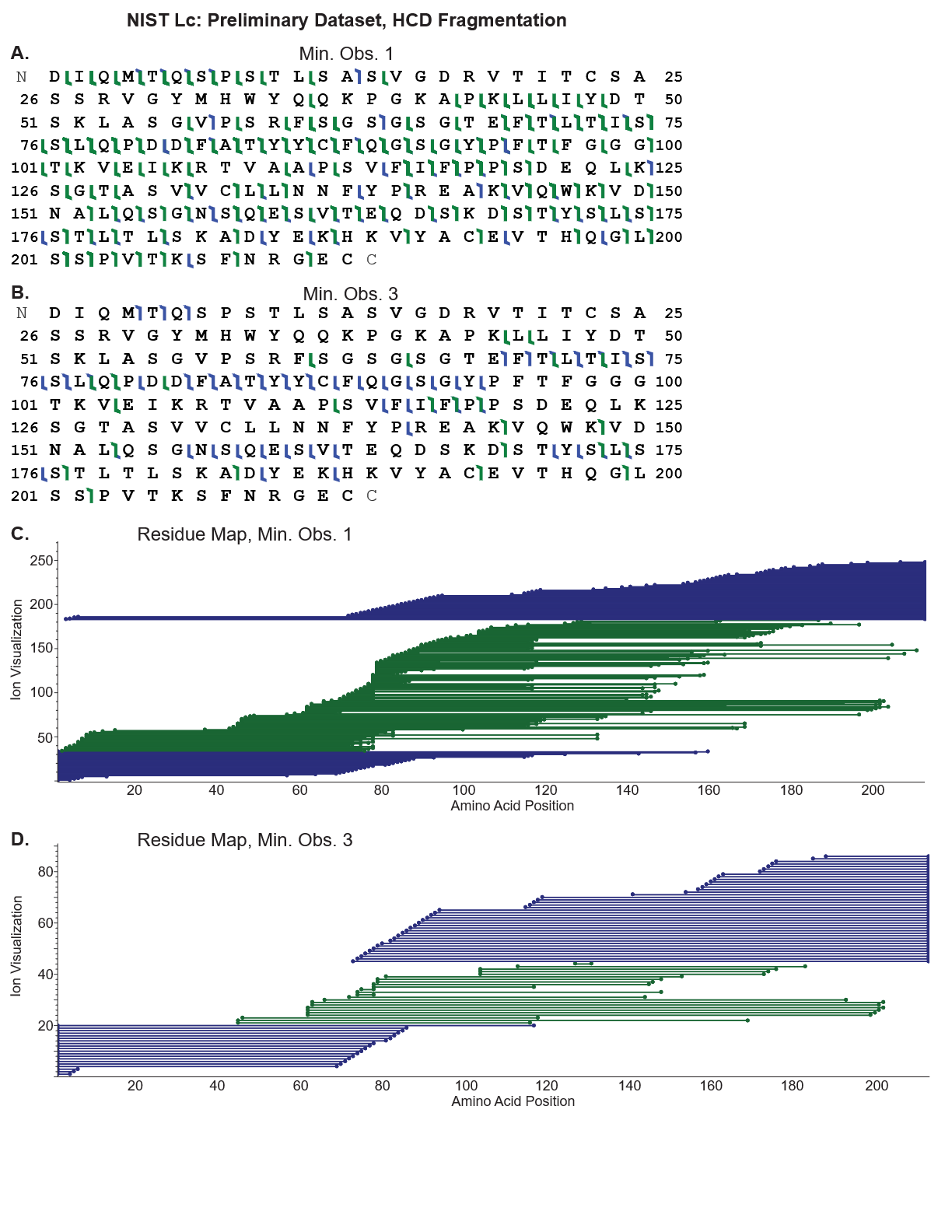


# Figure S16. Inclusion of Internal Ions for Analysis of HCD Fragmentation Data for NIST Lc Subunit.

The 3 HCD fragmentation replicates from the preliminary dataset were reanalyzed in Proteoform Studio with consideration of internal fragments included. Window mode “All” was used for spectral averaging. Terminal fragment detection used an *m/z* tolerance of 10 ppm, post-calibration *m/z* tolerance of 3 ppm, minimum isotope fit score of 0.68, and minimum S/N of 10.0. Internal fragment detection used an *m/z* tolerance of 3 ppm, post-calibration *m/z* tolerance of 1 ppm, minimum isotope fit score of 0.72, and minimum S/N of 30.0. Sequence coverage was visualized using fragment maps for a minimum number of observations for terminal and internal ions of 1 (A) and 3 (B). Additional visualization of sequence coverage and overlap between detected fragment ions are depicted in residue maps for terminal and internal ions with 1 (C) and 3 (B) minimum observations. Terminal b/y fragments are shown in blue, and internal fragments are shown in green.


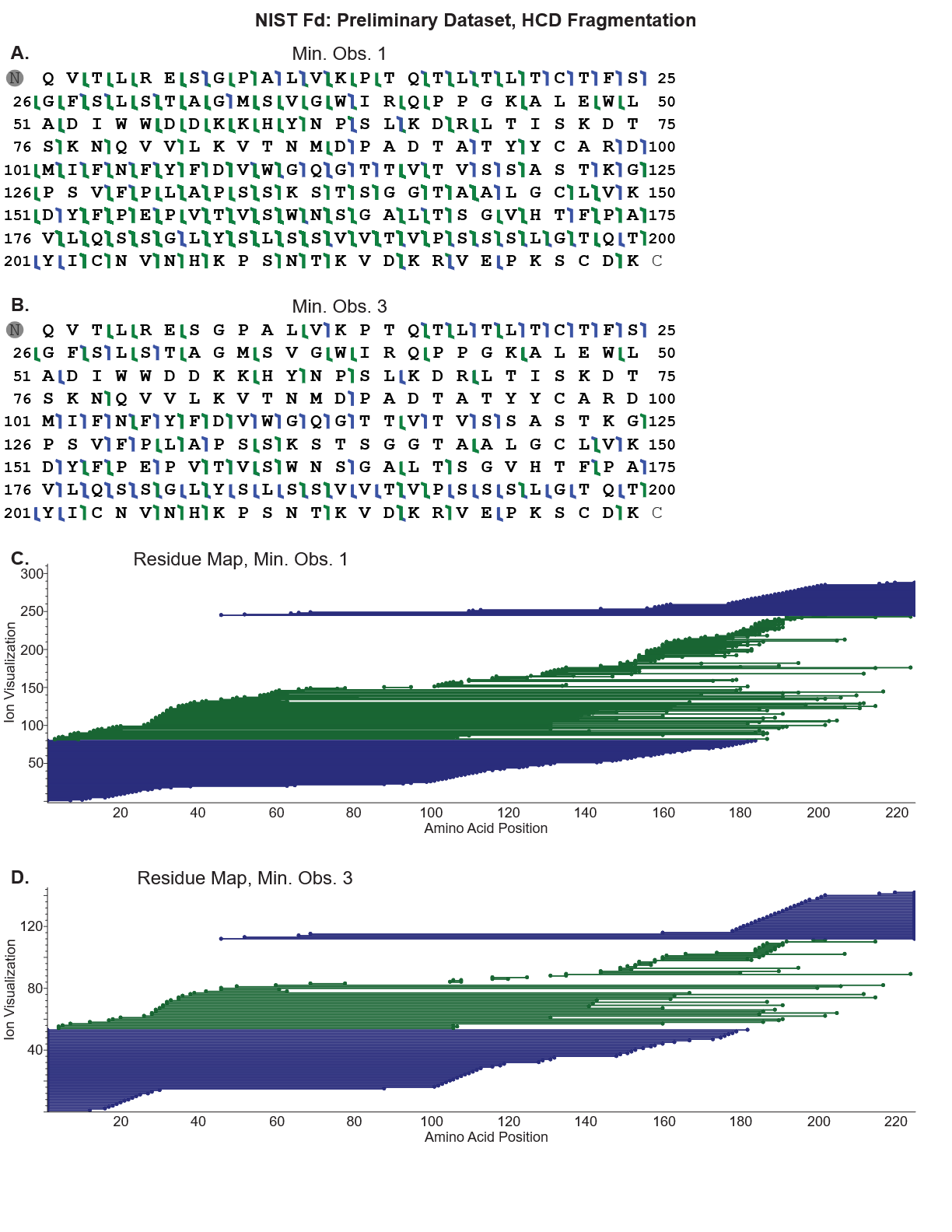


# Figure S17. Inclusion of Internal Ions for Analysis of HCD Fragmentation Data for NIST Fd Subunit.

The 3 HCD fragmentation replicates from the preliminary dataset were reanalyzed in Proteoform Studio with consideration of internal fragments included. Window mode “All” was used for spectral averaging. Terminal fragment detection used an *m/z* tolerance of 10 ppm, post-calibration *m/z* tolerance of 3 ppm, minimum isotope fit score of 0.68, and minimum S/N of 10.0. Internal fragment detection used an *m/z* tolerance of 3 ppm, post-calibration *m/z* tolerance of 1 ppm, minimum isotope fit score of 0.72, and minimum S/N of 30.0. Sequence coverage was visualized using fragment maps for a minimum number of observations for terminal and internal ions of 1 (A) and 3 (B). Additional visualization of sequence coverage and overlap between detected fragment ions are depicted in residue maps for terminal and internal ions with 1 (C) and 3 (B) minimum observations. Terminal b/y fragments are shown in blue, and internal fragments are shown in green.


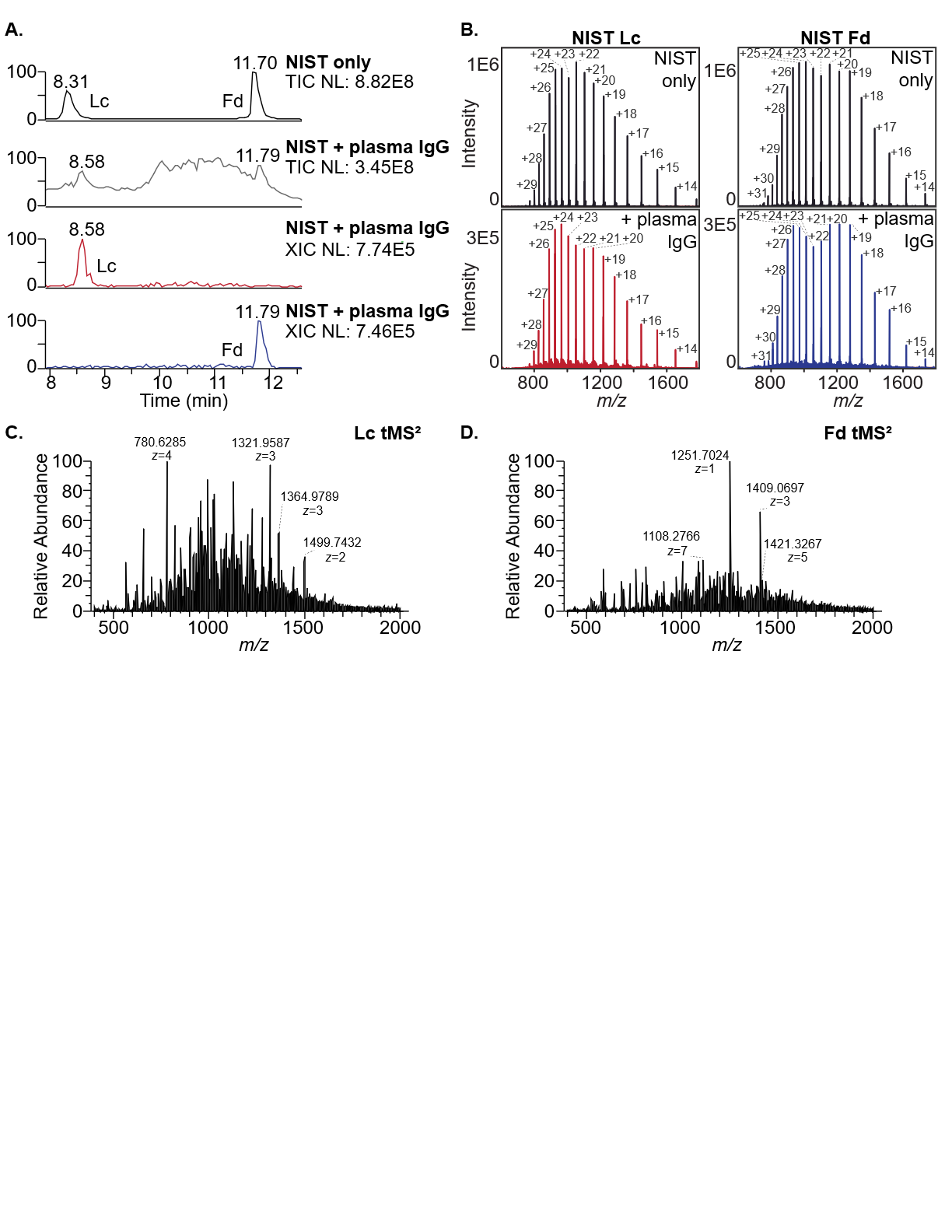


# Figure S18. NIST mAb Subunits Spiked into Plasma.

(A.) Ion Chromatograms including (top to bottom) total ion current (TIC) for sample of NIST only; TIC for NIST subunits spiked into plasma IgG background; extracted ion chromatogram (XIC), NIST Lc spiked into plasma IgG background; and XIC for NIST Fd spiked into plasma IgG background. Thermo FreeStyle was used to generate XICs using *m/z* values targeted in MS2 method (*m/z* 890.515 and *m/z* 971.809 for the Lc and Fd, respectively) with 20 ppm mass tolerance. (B.) Charge state distributions of NIST Lc and Fd for sample of NIST only (top panels) and NIST subunits spiked into plasma IgG background (bottom panels). Charge state distributions were generated using kDecon in the Interactive Deconvolution workflow in ProSight Native v1.0.25254. Averaged EThcD spectra from MS2 analysis of NIST subunits spiked into plasma IgG background are also shown for (C.) NIST Lc and (D.) NIST Fd.


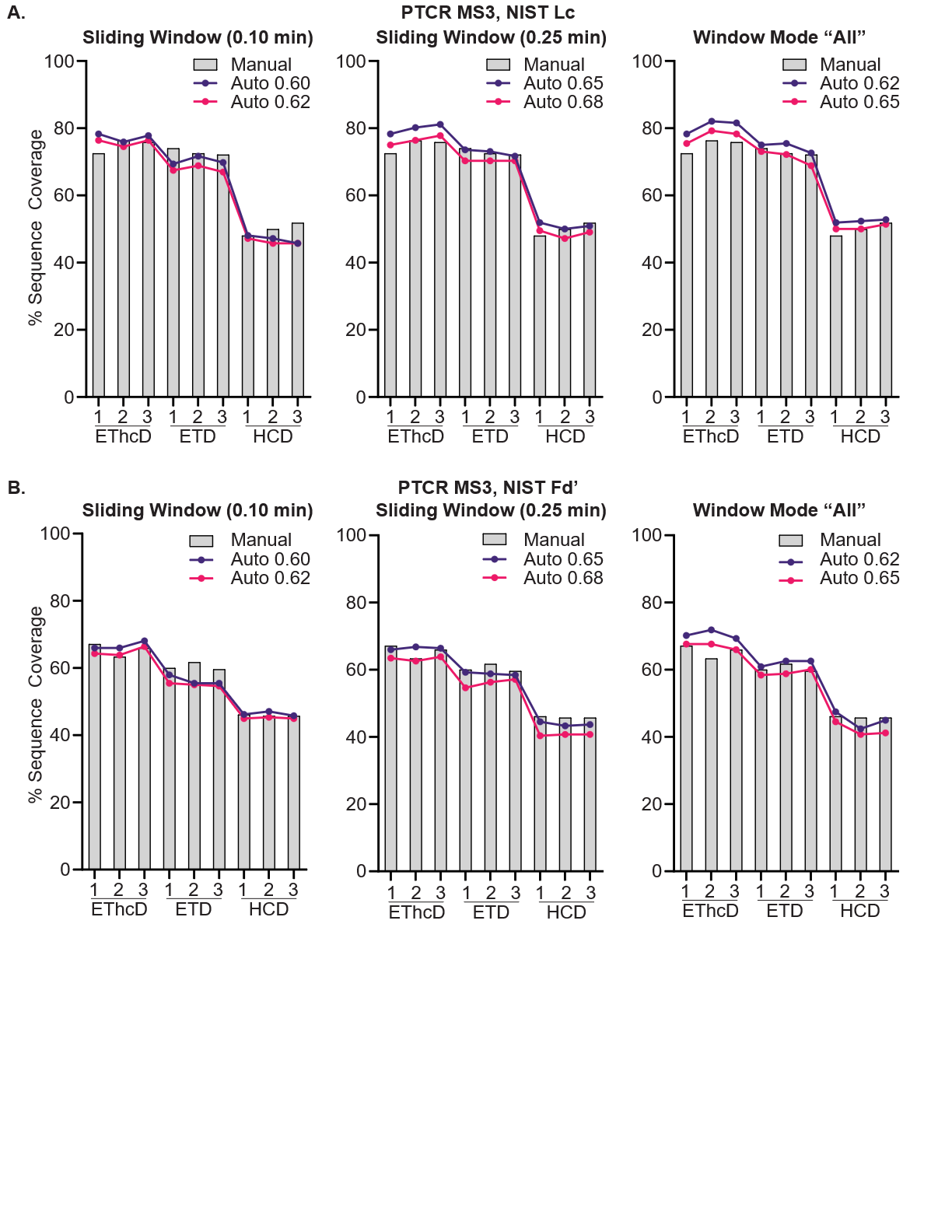


# Figure S19. Sequence Coverage Comparison, Automated Analysis in Proteoform Studio versus Manual Validation, Proton Transfer Charge Reduction (PTCR) MS3.

Subunits from below-hinge digestion and reduction of NIST mAb standard were analyzed by performing either EThcD, ETD, or HCD fragmentation prior to subjecting product ions to PTCR. PTCR experiments used an increased on-column load (1.5 μg versus 0.5 μg) and increased source voltage (3800 V versus 3400 V) compared to the MS2 experiments to offset the expected effect of signal dilution into multiple lower charge state channels after PTCR. Sequence coverage was evaluated manually using TDValidator in ProSight Native (version 1.0.25254) (“Manual”) and compared to results from automated processing in Proteoform Studio (“Auto”) using either a window width of 0.10 min and time to slide of 0.05 min (“Sliding Window [0.10 min]”), window width of 0.25 min and time to slide of 0.10 min (“Sliding Window [0.25 min]”), or window mode “all”, for fragmentation spectra averaging. Minimum S/N=10.0 and minimum number of observations of 1 for fragment ions were used in all cases. Results from 3 replicate injections for each fragmentation mode (EThcD, ETD, and HCD) are shown for (A) Lc subunit, and (B) Fd’ subunit. Minimum isotope fit score was adjusted to evaluate the setting providing consistency with manual validation results.

**References**

(1) Srzentic, K.; Nagornov, K. O.; Fornelli, L.; Lobas, A. A.; Ayoub, D.; Kozhinov, A. N.; Gasilova, N.; Menin, L.; Beck, A.; Gorshkov, M. V.; et al. Multiplexed Middle-Down Mass Spectrometry as a Method for Revealing Light and Heavy Chain Connectivity in a Monoclonal Antibody. *Anal Chem* **2018**, *90* (21), 12527-12535. DOI: 10.1021/acs.analchem.8b02398.

(2) Oates, R. N.; Lieu, L. B.; Kline, J. T.; Mullen, C.; Srzentic, K.; Huguet, R.; McAlister, G. C.; Huang, J.; Bergen, D.; Melani, R. D.; et al. Towards a universal method for middle-down analysis of antibodies via proton transfer charge reduction-Orbitrap mass spectrometry. *Anal Bioanal Chem* **2024**, *416* (28), 6463-6472. DOI: 10.1007/s00216-024-05534-z.

(3) Beaumal, C.; Desligniere, E.; Diemer, H.; Carapito, C.; Cianferani, S.; Hernandez-Alba, O. Improved characterization of trastuzumab deruxtecan with PTCR and internal fragments implemented in middle-down MS workflows. *Anal Bioanal Chem* **2024**, *416* (2), 519-532. DOI: 10.1007/s00216-023-05059-x.

(4) Formolo, T.; Ly, M.; Levy, M.; Kilpatrick, L.; Lute, S.; Phinney, K.; Marzilli, L.; Brorson, K.; Boyne, M.; Davis, D.; et al. Determination of the NISTmAb Primary Structure. In *State-of-the-Art and Emerging Technologies for Therapeutic Monoclonal Antibody Characterization Volume 2. Biopharmaceutical Characterization: The NISTmAb Case Study*, ACS Symposium Series, Vol. 1201; American Chemical Society, 2015; pp 1-62.

(5) Wei, B.; Lantz, C.; Ogorzalek Loo, R. R.; Campuzano, I. D. G.; Loo, J. A. Internal Fragments Enhance Middle-Down Mass Spectrometry Structural Characterization of Monoclonal Antibodies and Antibody-Drug Conjugates. *Analytical Chemistry* **2024**, *96* (6), 2491-2499. DOI: 10.1021/acs.analchem.3c04526.

(6) Mikawy, N. N.; Rojas Ramírez, C.; Defiglia, S. A.; Szot, C. W.; Le, J.; Lantz, C.; Wei, B.; Zenaidee, M. A.; Blakney, G. T.; Nesvizhskii, A. I.; et al. Are Internal Fragments Observable in Electron Based Top-Down Mass Spectrometry? *Molecular & Cellular Proteomics* **2024**, *23* (9), 100814. DOI: 10.1016/j.mcpro.2024.100814.

(7) Schmitt, N. D.; Berger, J. M.; Conway, J. B.; Agar, J. N. Increasing Top-Down Mass Spectrometry Sequence Coverage by an Order of Magnitude through Optimized Internal Fragment Generation and Assignment. *Analytical Chemistry* **2021**, *93* (16), 6355-6362. DOI: 10.1021/acs.analchem.0c04670.
